# Outcomes in pediatric studies of medium-chain acyl-coA dehydrogenase (MCAD) deficiency and phenylketonuria (PKU): a review

**DOI:** 10.1186/s13023-019-1276-1

**Published:** 2020-01-14

**Authors:** Michael Pugliese, Kylie Tingley, Andrea Chow, Nicole Pallone, Maureen Smith, Alvi Rahman, Pranesh Chakraborty, Michael T. Geraghty, Julie Irwin, Laure Tessier, Stuart G. Nicholls, Martin Offringa, Nancy J. Butcher, Ryan Iverson, Tammy J. Clifford, Sylvia Stockler, Brian Hutton, Karen Paik, Jessica Tao, Becky Skidmore, Doug Coyle, Kathleen Duddy, Sarah Dyack, Cheryl R. Greenberg, Shailly Jain Ghai, Natalya Karp, Lawrence Korngut, Jonathan Kronick, Alex MacKenzie, Jennifer MacKenzie, Bruno Maranda, John J. Mitchell, Murray Potter, Chitra Prasad, Andreas Schulze, Rebecca Sparkes, Monica Taljaard, Yannis Trakadis, Jagdeep Walia, Beth K. Potter, Canadian Inherited Metabolic Diseases Research Network

**Affiliations:** 1 School of Epidemiology and Public Health, University of Ottawa, Ottawa, Ontario Canada; 2 Canadian PKU & Allied Disorders Inc., Sparwood, Canada; 3 Canadian Organization for Rare Disorders, Ottawa, Canada; 4 Newborn Screening Ontario, Children’s Hospital of Eastern Ontario, Ottawa, Canada; 5 Division of Metabolics and Newborn Screening, Pediatrics, Children’s Hospital of Eastern Ontario and University of Ottawa, Ottawa, Canada; 6 Ambulatory Care, Children’s Hospital of Eastern Ontario, Ottawa, Canada; 7 Clinical Epidemiology Program, Ottawa Hospital Research Institute, Ottawa, Canada; 8 Department of Pediatrics, University of Toronto, Toronto, Canada; 9 Child Health Evaluative Sciences, The Hospital for Sick Children Research Institute, Toronto, Canada; 10 Biochemical Diseases, BC Children’s Hospital, Vancouver, Canada; 11 Faculty of Medicine, University of Ottawa, Ottawa, Canada; 12 Division of Medical Genetics, IWK Health Centre, Halifax, Canada; 13 Department of Pediatrics and Child Health, University of Manitoba, Winnipeg, Canada; 14 Department of Medical Genetics, University of Alberta, Edmonton, Canada; 15 Department of Pediatrics, Western University, London, Canada; 16 Department of Clinical Neurosciences, University of Calgary, Calgary, Canada; 17 Clinical and Metabolic Genetics, The Hospital for Sick Children, Toronto, Canada; 18 Children’s Hospital of Eastern Ontario Research Institute, Ottawa, Canada; 19 Department of Pediatrics, McMaster University, Hamilton, Canada; 20 Department of Pediatrics, Université de Sherbrooke, Sherbrooke, Canada; 21 Human Genetics and Pediatrics, McGill University, Montreal, Canada; 22 Pathology and Molecular Medicine, McMaster University, Hamilton, Canada; 23 Medical Genetics and Pediatrics, University of Calgary, Calgary, Canada; 24 Human Genetics and Medical Genetics, McGill University Health Centre, Montreal, Canada; 25 Department of Pediatrics, Queen’s University, Kingston, Canada

**Keywords:** PKU, MCAD deficiency, Core outcome sets, Rare diseases, Patient-oriented outcomes

## Abstract

**Background:**

Inherited metabolic diseases (IMDs) are a group of individually rare single-gene diseases. For many IMDs, there is a paucity of high-quality evidence that evaluates the effectiveness of clinical interventions. Clinical effectiveness trials of IMD interventions could be supported through the development of core outcome sets (COSs), a recommended minimum set of standardized, high-quality outcomes and associated outcome measurement instruments to be incorporated by all trials in an area of study. We began the process of establishing pediatric COSs for two IMDs, medium-chain acyl-CoA dehydrogenase (MCAD) deficiency and phenylketonuria (PKU), by reviewing published literature to describe outcomes reported by authors, identify heterogeneity in outcomes across studies, and assemble a candidate list of outcomes.

**Methods:**

We used a comprehensive search strategy to identify primary studies and guidelines relevant to children with MCAD deficiency and PKU, extracting study characteristics and outcome information from eligible studies including outcome measurement instruments for select outcomes. Informed by an established framework and a previously published pediatric COS, outcomes were grouped into five, mutually-exclusive, a priori core areas: growth and development, life impact, pathophysiological manifestations, resource use, and death.

**Results:**

For MCAD deficiency, we identified 83 outcomes from 52 articles. The most frequently represented core area was pathophysiological manifestations, with 33 outcomes reported in 29/52 articles (56%). Death was the most frequently reported outcome. One-third of outcomes were reported by a single study. The most diversely measured outcome was cognition and intelligence/IQ for which eight unique measurement instruments were reported among 14 articles. For PKU, we identified 97 outcomes from 343 articles. The most frequently represented core area was pathophysiological manifestations with 31 outcomes reported in 281/343 articles (82%). Phenylalanine concentration was the most frequently reported outcome. Sixteen percent of outcomes were reported by a single study. Similar to MCAD deficiency, the most diversely measured PKU outcome was cognition and intelligence/IQ with 39 different instruments reported among 82 articles.

**Conclusions:**

Heterogeneity of reported outcomes and outcome measurement instruments across published studies for both MCAD deficiency and PKU highlights the need for COSs for these diseases, to promote the use of meaningful outcomes and facilitate comparisons across studies.

## Background

Inherited metabolic diseases (IMD) are a large group of single-gene diseases that are individually rare but when aggregated have an estimated global birth prevalence of 50.9 in 100,000 live births [1]. These diseases are typically diagnosed early in life, often involve complex and resource-intensive medical care [2], and are frequently associated with intense home management and caregiving needs [3]. Providing effective treatment can be difficult due to a scarcity of evidence supporting current therapies [4]. When properly conducted, randomized controlled trials are considered the ‘gold standard’ primary study design for evaluating interventions [5]. However, trials have not always focused on those outcomes that are most relevant to patients diagnosed with the diseases being studied [6], and different trials within a single area of research have often incorporated disparate outcomes and outcome measurement instruments, thus impeding comparisons among studies and limiting capacity for data synthesis [7]. In response to these challenges, the Core Outcome Measures in Effectiveness Trials (COMET) Initiative [8] has led researchers in many disease areas to develop core outcome sets (COSs) [7]. A COS is a recommended minimum set of standardized, high-quality outcomes and associated outcome measurement instruments to be incorporated by all trials in an area of study [7]. COSs are developed to be relevant to all stakeholders in an area of research, including patients and their families, health care providers, and health policy decision-makers. Development and uptake of COSs aim to ensure that results can be synthesized and compared across studies.

It may be particularly valuable to develop COSs for IMDs and other rare diseases. Trials are less common and more challenging to implement for rare diseases relative to common diseases in part due to the difficulties in assembling a large enough cohort of patients to obtain adequate statistical power [9, 10]. Consequently, there may be great interest in comparing and synthesizing the results of all trials of one or more interventions for a rare disease (for example, using systematic reviews and meta-analyses) when considering evidence to support treatment and policy decisions. Consistent selection of outcomes and outcome measurement instruments across effectiveness trials would facilitate such evidence synthesis and make best use of the limited resources that are available for rigorous evaluative research for rare diseases. This is especially important for IMDs given the current rapid pace of development of new therapies [11], which has resulted in an increasing need for timely evidence regarding the effectiveness and comparative effectiveness of emerging and existing treatments. Given that clinical trial outcomes can determine the evidence considered by patients, clinicians, and policy advisors when making patient care and health policy decisions, the outcomes measured in future trials of interventions for IMDs should be carefully considered.

In the present study we sought to comprehensively review outcomes reported in previously published pediatric studies related to two of the most common IMDs, medium-chain acyl-coA dehydrogenase (MCAD) deficiency and phenylketonuria (PKU). For both MCAD deficiency and PKU, there are no existing COSs, limited evidence is available regarding new treatments, and there is a scarcity of trials reporting patient-oriented outcomes [12–16]. Our specific aims were to: (i) identify the unique outcomes that have been reported or recommended in the literature for these diseases; (ii) understand the scope and variation in outcomes reported and how they are measured; and (iii) create a list of candidate core outcomes to inform the development of pediatric COSs for MCAD deficiency and PKU [7]. We hypothesized that our review would identify substantial variation in the reporting of outcomes and outcome measurement instruments in the published MCAD deficiency and PKU literature.

## Materials and methods

### Protocol and registration

The study protocol was developed in collaboration with patient partners (MS, NP), registered in PROSPERO (CRD42017073524), and published [17]. This review is reported according to the Preferred Reporting Items for Systematic Reviews and Meta-Analyses (PRISMA) guidelines (see Additional file 1) [18].

### Search strategies and information sources

The study team developed separate search strategies to identify publications related to MCAD deficiency and PKU (further details, Additional file 1). Briefly, using the OVID platform, we searched Ovid MEDLINE® and Embase Classic+Embase. We also searched Cochrane Library databases. Language and publication year filters were applied to these searches to screen out non-English language studies, for practical reasons, and studies published before 1990 as the approach to diagnosing and managing these diseases has changed over time. We further searched the MEDLINE and EMBASE databases for publications focusing on long-term follow-up initiatives for the evaluation of newborn screening programs, as MCAD deficiency and PKU are among the most common targets of such programs [19]. Because of the anticipated low sensitivity of the database search for newborn screening evaluation initiatives, we conducted supplemental citation and related articles searches using key articles identified by the study team. Finally, we performed a grey literature search to identify further articles reporting or discussing outcomes for MCAD deficiency or PKU, guided by the Grey Matters tool [20]. Due to resource constraints, we limited the time devoted to grey literature searches to 15 h and prioritized sources that the study team deemed most relevant (see Additional file 1). As a further step in the grey literature search, we searched the COMET database [8] to identify COSs for pediatric conditions other than PKU and MCAD deficiency.

### Eligibility criteria

We screened retrieved citations against a priori eligibility criteria that were developed following the PICOS statement (population, intervention, comparator, outcome, study design). To be eligible for inclusion, articles had to be focused on populations of children (aged 18 years or younger) with MCAD deficiency or PKU. There were no criteria specified for interventions or comparators. Articles had to be primary studies reporting on five or more children; treatment guidelines or recommendations for outcomes to be measured in future studies; or COS studies (from the COMET database) for other pediatric health conditions. The decision to restrict to primary studies or recommendations was added post hoc to exclude reviews of primary studies that would have already been captured by our search strategy. We also made a post hoc decision to include pediatric COS studies identified from the COMET database only if they included potentially relevant outcomes not already captured from other sources. All eligible articles had to report or discuss one or more outcomes in relation to pediatric PKU, MCAD deficiency, and/or the long-term follow-up of newborn screened diseases. We used a modified version of the Outcome Measures in Rheumatology (OMERACT) filter 2.0 definition to define an outcome as a result that is amenable to change due to the effects of a health intervention [21]. We excluded citations that were published abstracts only.

### Study selection

We performed two levels of screening for all articles retrieved by our searches. For the peer-reviewed electronic database searches, level one (title/abstract) screening was conducted by two independent reviewers (among AR, KT, MP). We used a liberal accelerated screening approach, whereby a single reviewer needed to classify a citation as potentially eligible in order for it to advance to the next level but two reviewers had to independently exclude a citation in order for it to be removed. For the supplemental long-term follow-up, grey literature, and COMET database searches, a single author (MP) performed level one screening. For all searches, level two (full text) screening was conducted by two independent reviewers (among AR, KT, MP), who were required to fully agree on the inclusion or exclusion of an article; conflicts were resolved by consensus discussion or with a third team member.

### Data extraction and synthesis

One reviewer extracted outcomes from eligible studies into an electronic spreadsheet and a second reviewer verified the extracted data. Data collection included information about study characteristics, outcome names and definitions exactly as described by the study authors for outcomes meeting the modified OMERACT definition as described above, and outcome measurement instruments when these were specified by the authors.

Through group discussion among a subset of study investigators with clinical and methodological expertise (JI, PC, MTG, BKP), we combined and renamed outcomes that were conceptually similar to arrive at a set of ‘unique’ outcomes [22]. Outcomes representing a sub-concept of a broader outcome were kept separate when the outcome was thought to be particularly clinically important and was also perceived as a plausible trial outcome that could be selected over the broader outcome. Group discussion led to the creation of outcome domains to further group related outcomes based on an overarching concept (for example, physical growth and anthropometry, child life impact, health services use and costs) [7].

### Assignment of outcomes to core areas

We mapped outcome domains onto one of five, mutually-exclusive a priori defined core areas, four of which were described in the OMERACT Filter 2.0 framework, a literature-informed and consensus-developed process for establishing core outcome sets endorsed by COMET: death, life impact, pathophysiological manifestations, and resource use [7, 21]. The fifth core area of growth and development was included due to our focus on children, following the approach used in a previous pediatric COS study [22]. Mapping of domains onto core areas was achieved through group discussion and was based on the alignment of the domain's overarching theme with the description of the core areas from the original sources [21, 22]. The death core area covers general, disease-specific, and intervention-specific causes of death. The life impact core area includes quality of life, patient perceptions of health, psychosocial impact, and secondary impacts on caregivers. Resource use covers the direct and indirect economic impact of the disease on an individual and society. The pathophysiological manifestations core area covers the physiological and biochemical impact of the disease on the body’s organs and function, and also includes disease biomarkers [21]. The growth and development core area incorporates outcomes measuring the impact of the disease on the physical growth and cognitive development of the child [22].

## Results

### Identification of studies

**Fig. 1:**
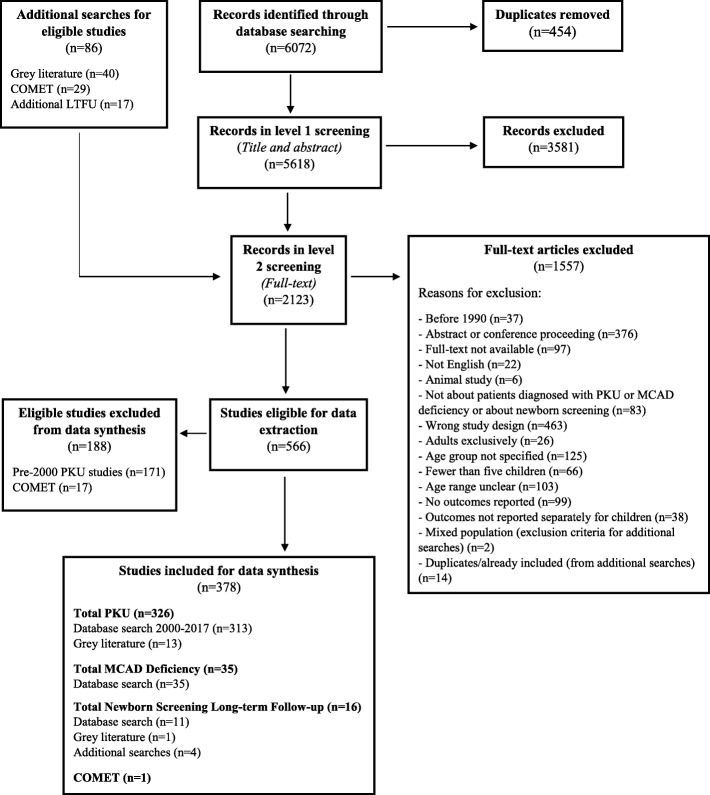
Flow diagram of articles included in data synthesis

The initial MCAD deficiency, PKU, and newborn screening long-term follow-up initiative database search strategies identified 6072 citations (Fig. 1). From these, 566 studies were determined to be eligible for data extraction. Due to time restraints and the large number of studies that were eligible for PKU, we decided to limit the initial data extraction for articles retrieved in the PKU search to only those that were published in the year 2001 or more recently; we then reviewed outcomes from studies from each previous year in turn, starting with 2000, until no new unique outcomes for PKU were identified. We initially extracted data from 308 eligible PKU-relevant articles published between 2001 and 2017. A review of an additional 16 PKU-relevant articles published in 2000 did not identify any additional unique outcomes so that PKU-relevant studies were excluded when published before the year 2000 (see Additional file 2). Only one of the 18 eligible articles from the COMET search reported a new relevant outcome that had not been identified by studies from the other searches and was retained for data synthesis. In total, 378 articles were included in our review (see Additional file 3).

### Outcomes for MCAD deficiency

#### Study characteristics

Our search strategies identified a total of 52 articles relevant to MCAD deficiency: 35 MCAD deficiency studies were identified from our disease-specific database searches, 16 from newborn screening long-term follow-up studies, and one from a previously published pediatric COS. Most articles were published after 2009 (58%) and reported on observational studies (85%) (Table 1). With respect to participant ages in primary studies, the focus was commonly on newborns only (45%) or children (40%), with a minority of studies including children and adults. Articles reported or discussed a median of four unique outcomes (range = 1–28) with most including five or fewer outcomes per article (67%).

**Table 1:** Characteristics of studies included in data synthesis

MCAD deficiency (*n* = 52)	n (%)	PKU (*n* = 343)	n (%)
Publication Year			
1994–1999	5 (10%)	2000–2004	84 (24%)
2000–2009	17 (33%)	2005–2009	67 (20%)
2010–2018	30 (58%)	2010–2014	120 (35%)
		2015–2018	72 (21%)
Country of Origin			
Australia	5 (10%)	Australia	10 (3%)
Germany	7 (13%)	Germany	37 (11%)
Iran	1 (2%)	Iran	11 (3%)
Italy	0 (0%)	Italy	25 (7%)
The Netherlands	5 (10%)	The Netherlands	31 (9%)
Spain	3 (6%)	Spain	26 (8%)
United Kingdom	5 (10%)	United Kingdom	26 (8%)
United States	20 (38%)	United States	68 (20%)
Other country	6 (12%)	Other country	109 (32%)
Study Design			
Core outcome set	1 (2%)	Core outcome set	1 (< 1%)
Guidelines/recommendations	5 (10%)	Guidelines/recommendations	18 (5%)
Observational study	44 (85%)	Observational study	283 (83%)
Qualitative	1 (2%)	Qualitative	2 (1%)
Trial	1 (2%)	Trial	39 (11%)
Median (IQR) Sample Size			
*n* = 45^a^	59 (37–180)	*n* = 315^c^	54 (30–106)
Age Range of Study Participants			
*n* = 47^b^		*n* = 325^d^	
Newborns only	21 (45%)	Newborns only	35 (11%)
Children	19 (40%)	Infants	2 (1%)
Children and adults combined	6 (13%)	Children	181 (56%)
Children and maybe adults	1 (2%)	Children and adults combined	104 (32%)
		Children and maybe adults	3 (1%)
Number of outcomes reported/discussed per article			
1	11 (21%)	1	83 (24%)
2–5	24 (46%)	2–5	180 (52%)
6–10	10 (19%)	6–10	51 (15%)
11 or more	7 (13%)	11 or more	29 (8%)

^a^Excluded five guidelines/recommendations that did not collect samples, one study where the samples size was not clear, and one registered clinical trial where only target sample size was reported

^b^Excluded five guidelines/recommendations that did not include participants

^c^Excluded 18 guidelines/recommendations which did not collect samples, three studies where the samples size was not clear, and seven registered clinical trials where only target sample size was reported

^d^Excluded 18 guidelines/recommendations which did not include participants

#### MCAD deficiency outcomes within domains and core areas

Data extraction initially yielded 230 MCAD deficiency outcomes. The study team combined outcomes covering similar concepts, yielding 83 unique MCAD deficiency outcomes (Table 2). Unique outcomes were grouped into 10 domains within the core areas. There was a relatively even distribution of studies measuring outcomes across the core areas for MCAD deficiency. The most frequently reported and most diverse core area was pathophysiological manifestations, which contained three domains and 33 unique outcomes reported by 29/52 articles (56%).

**Table 2:** Unique MCAD deficiency outcomes reported in studies included for data synthesis

Outcome	# (%) of articles TOTAL (*n* = 52)	# (%) articles pre-2000 (*n* = 5)	# (%) articles 2000–2009 (*n* = 17)	# (%) articles after 2009 (*n* = 30)
1. CORE AREA: GROWTH AND DEVELOPMENT	26 (50%)	4 (80%)	9 (53%)	13 (43%)
Domain: Physical Growth and Anthropometry	11 (21%)	1 (20%)	1 (6%)	9 (30%)
Body mass index	2 (4%)	0 (0%)	1 (6%)	1 (3%)
Growth	5 (10%)	1 (20%)	0 (0%)	4 (13%)
Head circumference	2 (4%)	0 (0%)	0 (0%)	2 (7%)
Height/length	4 (8%)	0 (0%)	1 (6%)	3 (10%)
Weight^10*^	6 (12%)	0 (0%)	1 (6%)	5 (17%)
Domain: Cognition and Development	23 (44%)	4 (80%)	8 (47%)	11 (37%)
Cognition and intelligence/IQ^2*^	14 (27%)	2 (40%)	4 (24%)	8 (27%)
Overall child development^2*^	14 (27%)	3 (60%)	5 (29%)	6 (20%)
Sensorimotor and motor functioning^7*^	7 (13%)	0 (0%)	2 (12%)	5 (17%)
Learning difficulties/disabilities	2 (4%)	0 (0%)	2 (12%)	0 (0%)
School function and placement	5 (10%)	0 (0%)	1 (6%)	4 (13%)
2. CORE AREA: LIFE IMPACT	25 (48%)	3 (60%)	6 (35%)	16 (53%)
Domain: Child and Caregiver/Family Life Impact	15 (29%)	1 (20%)	3 (18%)	11 (37%)
Child quality of life	1 (2%)	0 (0%)	0 (0%)	1 (3%)
Overall clinician-assessed health status of child^10*^	6 (12%)	1 (20%)	0 (0%)	5 (17%)
Caregiver/family psychosocial well-being	2 (4%)	0 (0%)	2 (12%)	0 (0%)
Parental experiences with illness care and prevention	1 (2%)	0 (0%)	0 (0%)	1 (3%)
Caregiver/family economic impact	3 (6%)	0 (0%)	1 (6%)	2 (7%)
Physical activity participation and tolerance	1 (2%)	0 (0%)	0 (0%)	1 (3%)
Achievement of treatment goals	1 (2%)	0 (0%)	0 (0%)	1 (3%)
Domain: Child Behaviour, Mental Health, and Temperament	5 (10%)	2 (40%)	0 (0%)	3 (10%)
Behaviour problems and externalizing mental health or behaviour disorders	4 (8%)	2 (40%)	0 (0%)	2 (7%)
Attention-deficit hyperactivity disorder (ADHD) or ADHD-like symptoms	2 (4%)	1 (20%)	0 (0%)	1 (3%)
Internalizing mental health or mood disorders and associated symptoms	1 (2%)	0 (0%)	0 (0%)	1 (3%)
Autism spectrum disorder (ASD) or ASD-like symptoms	1 (2%)	0 (0%)	0 (0%)	1 (3%)
Tic disorder	1 (2%)	0 (0%)	0 (0%)	1 (3%)
Domain: Disease Management and Feeding Behaviour	15 (29%)	2 (40%)	4 (24%)	9 (30%)
Age at treatment initiation	1 (2%)	0 (0%)	1 (6%)	0 (0%)
Possession or use of an emergency card or letter	1 (2%)	0 (0%)	1 (6%)	0 (0%)
Frequency of dietary analysis	1 (2%)	0 (0%)	0 (0%)	1 (3%)
Overall dietary intake relative to standards	1 (2%)	0 (0%)	0 (0%)	1 (3%)
Infant feeding difficulties	1 (2%)	0 (0%)	0 (0%)	1 (3%)
Feeding difficulties	2 (4%)	1 (20%)	0 (0%)	1 (3%)
Use of a feeding tube	3 (6%)	0 (0%)	1 (6%)	2 (7%)
Diet modification	3 (6%)	0 (0%)	1 (6%)	2 (7%)
Fasting	3 (6%)	1 (20%)	0 (0%)	2 (7%)
Fat restriction	1 (2%)	0 (0%)	1 (6%)	0 (0%)
Carnitine supplementation	5 (10%)	0 (0%)	2 (12%)	3 (10%)
Cornstarch supplementation	2 (4%)	0 (0%)	1 (6%)	1 (3%)
Fatty acid supplementation	1 (2%)	0 (0%)	1 (6%)	0 (0%)
Vitamin supplementation	1 (2%)	0 (0%)	0 (0%)	1 (3%)
Prescription or use of medication, supplements, medical foods other than carnitine, cornstarch, or vitamins	2 (4%)	0 (0%)	0 (0%)	2 (7%)
Supplementation with rapidly available carbohydrates during acute illness	2 (4%)	0 (0%)	2 (12%)	0 (0%)
Sick day plan	2 (4%)	0 (0%)	1 (6%)	1 (3%)
Prescription of use of medications or supplements unrelated to MCAD deficiency	2 (4%)	1 (20%)	0 (0%)	1 (3%)
Adherence to prescribed or recommended treatment or management strategy	2 (4%)	0 (0%)	1 (6%)	1 (3%)
3. CORE AREA: RESOURCE USE	18 (35%)	1 (20%)	7 (41%)	10 (33%)
Domain: Health Service Use and Costs	18 (35%)	1 (20%)	7 (41%)	10 (33%)
Access to care	2 (4%)	0 (0%)	0 (0%)	2 (7%)
Costs of care	2 (4%)	0 (0%)	1 (6%)	1 (3%)
Emergency department use^10*^	6 (12%)	0 (0%)	1 (6%)	5 (17%)
Hospitalization^2*^	14 (27%)	1 (20%)	5 (29%)	8 (27%)
Outpatient care use^10*^	6 (12%)	0 (0%)	2 (12%)	4 (13%)
Genetic counseling and family cascade carrier testing	4 (8%)	0 (0%)	0 (0%)	4 (13%)
Health education service use	1 (2%)	0 (0%)	0 (0%)	1 (3%)
Provision and coordination of services	1 (2%)	0 (0%)	1 (6%)	0 (0%)
4. CORE AREA: DEATH	24 (46%)	2 (40%)	9 (53%)	13 (43%)
Domain: Death	24 (46%)	2 (40%)	9 (53%)	13 (43%)
Death^1^	24 (46%)	2 (40%)	9 (53%)	13 (43%)
5. CORE AREA: PATHOPHYSIOLOGICAL MANIFESTATIONS	29 (56%)	5 (100%)	9 (53%)	15 (50%)
Domain: Acute Disease-specific Manifestations	22 (42%)	3 (60%)	7 (41%)	12 (40%)
Metabolic decompensation^2*^	14 (27%)	3 (60%)	4 (24%)	7 (23%)
Encephalopathy^10*^	6 (12%)	1 (20%)	3 (18%)	2 (7%)
Seizures^7*^	7 (13%)	2 (40%)	2 (12%)	3 (10%)
Cardiovascular symptoms and disorders	3 (6%)	0 (0%)	1 (6%)	2 (7%)
Respiratory symptoms and disorders	3 (6%)	0 (0%)	1 (6%)	2 (7%)
Muscle symptoms and disorders	4 (8%)	0 (0%)	1 (6%)	2 (7%)
Hypoglycaemia^7*^	7 (13%)	1 (20%)	3 (18%)	3 (10%)
Hyperammonemia	2 (4%)	0 (0%)	0 (0%)	2 (7%)
Hyperuricemia	1 (2%)	0 (0%)	0 (0%)	1 (3%)
Ketonuria	2 (4%)	0 (0%)	0 (0%)	2 (7%)
Metabolic acidosis	2 (4%)	0 (0%)	0 (0%)	2 (7%)
Psychogenic blindness	1 (2%)	1 (20%)	0 (0%)	0 (0%)
Chronic sequelae of an acute event	4 (8%)	3 (60%)	1 (6%)	0 (0%)
Domain: Non Disease-specific Symptoms and Disorders	13 (25%)	3 (60%)	5 (29%)	5 (17%)
Neurological symptoms and disorders	1 (2%)	0 (0%)	1 (6%)	0 (0%)
Signs of discomfort	1 (2%)	0 (0%)	0 (0%)	1 (3%)
Pallor	2 (4%)	0 (0%)	0 (0%)	2 (7%)
Dehydration	2 (4%)	0 (0%)	0 (0%)	2 (7%)
Acute infections^6^	10 (19%)	3 (60%)	4 (24%)	3 (10%)
Body temperature abnormalities	2 (4%)	0 (0%)	1 (6%)	1 (3%)
Gastrointestinal symptoms and disorders	3 (6%)	1 (20%)	1 (6%)	1 (3%)
Hyperglycaemia	1 (2%)	0 (0%)	0 (0%)	1 (3%)
Bronchospasms	1 (2%)	0 (0%)	0 (0%)	1 (3%)
Domain: Biomarkers of Nutritional and Organ-specific Health	11 (21%)	1 (20%)	3 (18%)	7 (23%)
Acylcarnitines	3 (6%)	0 (0%)	3 (18%)	0 (0%)
Free carnitine	1 (2%)	0 (0%)	0 (0%)	1 (3%)
Total carnitine	5 (10%)	0 (0%)	2 (12%)	3 (10%)
Fasting tolerance biomarkers	1 (2%)	0 (0%)	0 (0%)	1 (3%)
Nutritional assessment biomarkers	5 (10%)	0 (0%)	2 (12%)	3 (10%)
Inflammation biomarkers	1 (2%)	0 (0%)	0 (0%)	1 (3%)
Liver health biomarkers	3 (6%)	0 (0%)	1 (6%)	2 (7%)
Kidney health biomarkers	2 (4%)	0 (0%)	0 (0%)	2 (7%)
Muscle health biomarkers	3 (6%)	1 (20%)	1 (6%)	1 (3%)
Neurological health biomarkers	1 (2%)	0 (0%)	0 (0%)	1 (3%)
General health biomarkers	2 (4%)	0 (0%)	0 (0%)	2 (7%)

^1–10^indicates top ten most reported or discussed unique outcomes, ties indicated with an asterisks

Death was the most commonly reported or discussed unique outcome for MCAD deficiency (24/52 articles, 46%) (Table 2). The next most frequently reported outcomes were cognition and intelligence/IQ, hospitalization, metabolic decompensation, and overall child development, which were each included in 14 articles (27% of articles). Among the other frequently reported outcomes (reported in more than five studies), four were in the pathophysiological manifestations core area (three in the acute disease-specific manifestations domain, one in the non disease-specific symptoms and disorders domain), two were in the growth and development core area (one in the cognition and development domain, and one in the physical growth and anthropometry domain), two in the resource use core area in the health service use and costs domain, and one in the life impact core area under the child and caregiver/family life impact domain. One-third of the outcomes (27/83 outcomes) were reported by a single article.

#### Changes over time to reported outcomes for MCAD deficiency

We observed changes over time in the frequency of reporting for some MCAD deficiency outcomes identified in our review (Table 2). For example, within the growth and development domain, there was an increase in reporting of outcomes in the physical growth and anthropometry domain: one of 17 articles (6%) published between 2000 and 2009 reported outcomes within this domain compared with 9 of 30 articles (30%) published after 2009. There was a modest decrease in reporting of outcomes in the cognition and development domain (8/17 or 47% from 2000 to 2009 vs. 11/30 or 37% after 2009). There was also an increase in reporting of outcomes in the life impact core area (6/17 or 35% from 2000 to 2009 vs. 16/30 or 53% after 2009) including the outcome, overall clinician-assessed health status of child (0/17 or 0% from 2000 to 2009 vs. 5/30 or 17% after 2009). Other changes included decreased reporting of acute infections (4/17 or 24% from 2000 to 2009 vs. 3/30 or 10% after 2009) and acylcarnitines (3/17 from 2000 to 2009 or 18% vs. 0/30 or 0% after 2009).

#### Outcome measurement instruments for selected MCAD deficiency outcomes

We summarized data for outcome measurement instruments associated with neuro-psychological outcomes and/or outcomes that were typically measured using self- or parent-reported questionnaires. Among 25 articles measuring such outcomes for MCAD deficiency, we identified 11 outcome measurement instruments associated with 11 unique outcomes (see Additional file 4). The most diversely measured outcome was cognition and intelligence/IQ: eight unique instruments were reported by 14 articles, with the Wechsler Intelligence Scales family of measurement instruments [23] being the most frequently specified (3/14 articles, 21%). The only other outcome with more than one reported measurement instrument was sensorimotor and motor functioning with four unique tools used in seven articles. One measurement instrument was specified for each of caregiver/family psychosocial well-being, behaviour problems and externalizing mental health or behaviours disorders, internalizing behaviour, and overall child development. Studies reporting child quality of life, parental experiences with illness care and prevention, attention-deficit hyperactivity disorder (ADHD) or ADHD-like symptoms, autism spectrum disorder (ASD) or ASD-like symptoms, and tic disorder were unclear about which outcome measurement instruments were used. There were not enough data available to assess any changes over time in how outcomes were measured for MCAD deficiency.

### Outcomes for PKU

#### Study characteristics

PKU data synthesis included 343 articles consisting of 326 PKU-specific articles, 16 newborn screening long-term follow-up studies, and one pediatric COS. Similar to MCAD deficiency, over half of the articles included for PKU data synthesis were published after 2009 (56%) and mainly consisted of observational studies (83%) (Table 1). A majority of articles were focused on children (56%), with smaller proportions including children and adults combined (32%) or newborns only (11%). A number of studies clearly included children but did not specify the age ranges of the included participants and, as described for MCAD deficiency, further results were not broken down by age group. Articles reported or discussed a median of three unique outcomes (range = 1–25), with the majority including five or fewer outcomes (76%).

#### PKU outcomes within domains and core areas

Data extraction initially yielded 565 outcomes. The study team combined outcomes covering similar concepts into 97 unique PKU outcomes (Table 3). Unique outcomes were grouped into 11 domains within the five core areas. The most frequently represented core area was pathophysiological manifestations, which contained two domains and 31 unique outcomes reported by 281/343 articles (82%). The most diverse core area was life impact with five domains and 44 unique outcomes reported by 156/343 articles (45%).

**Table 3:** Unique PKU outcomes reported in studies included for data synthesis

Outcome	# (%) of articles TOTAL (*n* = 343)	# (%) of articles 2000–2004 (*n* = 84)	# (%) articles 2005–2009 (*n* = 67)	# (%) articles 2010–2014 (*n* = 120)	# (%) articles after 2014 (*n* = 72)
1. CORE AREA: GROWTH AND DEVELOPMENT	153 (45%)	40 (48%)	28 (42%)	53 (44%)	32 (44%)
Domain: Physical Growth and Anthropometry	63 (18%)	6 (7%)	14 (21%)	25 (21%)	18 (25%)
Body mass index, overweight, or obesity status^8*^	35 (10%)	4 (5%)	3 (4%)	14 (12%)	14 (19%)
Growth	16 (5%)	1 (1%)	5 (7%)	8 (7%)	2 (3%)
Head circumference	8 (2%)	1 (1%)	1 (1%)	3 (3%)	3 (4%)
Height/length^5*^	42 (12%)	4 (5%)	7 (10%)	18 (15%)	13 (18%)
Weight	38 (11%)	2 (2%)	9 (13%)	16 (13%)	11 (15%)
Body composition	9 (3%)	1 (1%)	2 (3%)	4 (3%)	2 (3%)
Domain: Cognition and Development	110 (32%)	34 (40%)	19 (28%)	38 (32%)	19 (26%)
Cognition and intelligence/IQ^2^	82 (24%)	25 (30%)	14 (21%)	29 (24%)	14 (19%)
Overall child development	11 (3%)	3 (4%)	1 (1%)	5 (4%)	2 (3%)
Sensorimotor and motor functioning^10*^	32 (9%)	9 (11%)	4 (6%)	17 (14%)	2 (3%)
Learning difficulties/disabilities	1 (0%)	0 (0%)	1 (1%)	0 (0%)	0 (0%)
Academic achievement/school performance	20 (6%)	5 (6%)	1 (1%)	9 (8%)	5 (7%)
Executive functioning^10*^	32 (9%)	9 (11%)	5 (7%)	11 (9%)	7 (10%)
2. CORE AREA: LIFE IMPACT	156 (45%)	28 (33%)	24 (36%)	61 (51%)	43 (60%)
Domain: Child Life Impact	35 (10%)	4 (5%)	1 (1%)	20 (17%)	10 (14%)
Child quality of life	21 (6%)	2 (2%)	1 (1%)	12 (10%)	6 (8%)
Child psychosocial well-being and self-concept	15 (4%)	0 (0%)	0 (0%)	10 (8%)	5 (7%)
Child social impact and function	12 (3%)	2 (2%)	0 (0%)	6 (5%)	4 (6%)
Bodily pain or discomfort	7 (2%)	0 (0%)	0 (0%)	4 (3%)	3 (4%)
Sleep problems	1 (0%)	0 (0%)	0 (0%)	1 (1%)	0 (0%)
Overall clinician-assessed health status of child	4 (1%)	0 (0%)	0 (0%)	3 (3%)	1 (1%)
Child understanding of and self-efficacy with management of PKU	3 (1%)	1 (1%)	0 (0%)	1 (1%)	1 (1%)
Achievement of treatment goals	1 (0%)	0 (0%)	0 (0%)	0 (0%)	1 (1%)
Domain: Caregiver/family Life Impact	19 (6%)	4 (5%)	1 (1%)	7 (6%)	7 (10%)
Impact of PKU on caregiver/family quality of life	10 (3%)	0 (0%)	0 (0%)	5 (4%)	5 (7%)
Caregiver/family psychosocial well-being	9 (3%)	4 (5%)	0 (0%)	2 (2%)	3 (4%)
Caregiver/family economic impact	5 (1%)	1 (1%)	1 (1%)	2 (2%)	1 (1%)
Impact of PKU on caregiver/family diet	1 (0%)	0 (0%)	0 (0%)	1 (1%)	0 (0%)
Domain: Child and Caregiver/family Life Impact	3 (1%)	1 (1%)	0 (0%)	0 (0%)	2 (3%)
Strategies used by parents to help children cope with PKU	1 (0%)	0 (0%)	0 (0%)	0 (0%)	1 (1%)
Perceived control over behavior and skills	2 (1%)	1 (1%)	0 (0%)	0 (0%)	1 (1%)
Domain: Child Behaviour, Mental Health, and Temperament	42 (12%)	8 (10%)	4 (6%)	19 (16%)	11 (15%)
Behaviour problems and externalizing mental health or behaviour disorders	19 (6%)	5 (6%)	2 (3%)	8 (7%)	4 (6%)
Attention-deficit hyperactivity disorder (ADHD) or ADHD-like symptoms	15 (4%)	2 (2%)	1 (1%)	8 (7%)	4 (6%)
Internalizing mental health or mood disorders and associated symptoms	26 (8%)	4 (5%)	1 (1%)	15 (13%)	6 (8%)
Autism spectrum disorder (ASD) or ASD-like symptoms	9 (3%)	0 (0%)	1 (1%)	5 (4%)	3 (4%)
Atypical behaviour and mental symptoms other than those specified	1 (0%)	0 (0%)	0 (0%)	1 (1%)	0 (0%)
Temperament/personality	4 (1%)	1 (1%)	0 (0%)	3 (3%)	0 (0%)
Domain: Disease Management and Feeding Behaviour	113 (33%)	19 (23%)	21 (31%)	41 (34%)	32 (44%)
Age at treatment initiation	4 (1%)	1 (1%)	0 (0%)	3 (3%)	0 (0%)
Infant breast and formula feeding	2 (1%)	0 (0%)	0 (0%)	1 (1%)	1 (1%)
Dietary intake of phenylalanine	28 (8%)	5 (6%)	5 (7%)	10 (8%)	8 (11%)
Dietary intake of amino acids other than phenylalanine	6 (2%)	2 (2%)	0 (0%)	3 (3%)	1 (1%)
Dietary intake of medical foods or formula, modified low-protein foods, and protein substitutes	9 (3%)	2 (2%)	0 (0%)	6 (5%)	1 (1%)
Dietary intake of protein^5*^	42 (12%)	9 (11%)	5 (7%)	20 (17%)	8 (11%)
Dietary intake of energy	30 (9%)	8 (10%)	6 (9%)	11 (9%)	5 (7%)
Overall dietary intake relative to standards	5 (1%)	0 (0%)	0 (0%)	3 (3%)	2 (3%)
Dietary intake of fat	17 (5%)	5 (6%)	2 (3%)	6 (5%)	4 (6%)
Dietary intake of specific fatty acids	16 (5%)	6 (7%)	4 (6%)	4 (3%)	2 (3%)
Dietary intake of cholesterol	7 (2%)	3 (4%)	1 (1%)	3 (3%)	0 (0%)
Dietary intake of carbohydrates	15 (4%)	3 (4%)	3 (4%)	6 (5%)	3 (4%)
Dietary intake of fibre	7 (2%)	3 (4%)	1 (1%)	2 (2%)	1 (1%)
Glycemic index of foods consumed	1 (0%)	0 (0%)	0 (0%)	0 (0%)	1 (1%)
Dietary intake of vitamins and minerals	19 (6%)	6 (7%)	1 (1%)	6 (5%)	6 (8%)
Dietary intake of major food groups	4 (1%)	0 (0%)	0 (0%)	2 (2%)	2 (3%)
Eating behaviour	2 (1%)	0 (0%)	0 (0%)	1 (1%)	1 (1%)
Frequency of dietary analysis	1 (0%)	0 (0%)	0 (0%)	1 (1%)	0 (0%)
Adherence to PKU diet	27 (8%)	1 (1%)	7 (10%)	7 (6%)	12 (17%)
Prescriptions of use of medications to manage PKU	1 (0%)	0 (0%)	0 (0%)	1 (1%)	0 (0%)
Phenylalanine tolerance	26 (8%)	1 (1%)	7 (10%)	7 (6%)	11 (15%)
Liberalization of PKU diet	3 (1%)	1 (1%)	0 (0%)	1 (1%)	1 (1%)
Child acceptability of PKU diet	14 (4%)	2 (2%)	1 (1%)	6 (5%)	5 (7%)
Caregiver/family acceptability of PKU diet	11 (3%)	0 (0%)	2 (3%)	7 (6%)	2 (3%)
3. CORE AREA: RESOURCE USE	11 (3%)	2 (2%)	1 (1%)	7 (6%)	1 (1%)
Domain: Health Service Use and Costs	11 (3%)	2 (2%)	1 (1%)	7 (6%)	1 (1%)
Access to care	1 (0%)	0 (0%)	0 (0%)	0 (0%)	1 (1%)
Costs of care	1 (0%)	0 (0%)	0 (0%)	1 (1%)	0 (0%)
Emergency department use	3 (1%)	0 (0%)	0 (0%)	3 (3%)	0 (0%)
Hospitalization	8 (2%)	2 (2%)	0 (0%)	6 (5%)	0 (0%)
Outpatient care use	5 (1%)	2 (2%)	0 (0%)	3 (3%)	0 (0%)
Genetic counseling and family cascade carrier testing	4 (1%)	0 (0%)	0 (0%)	3 (3%)	1 (1%)
Health education service use	1 (0%)	0 (0%)	0 (0%)	1 (1%)	0 (0%)
Use of medical devices	3 (1%)	2 (2%)	0 (0%)	1 (1%)	0 (0%)
Provision and coordination of services	2 (1%)	1 (1%)	1 (1%)	0 (0%)	0 (0%)
4. CORE AREA: DEATH	9 (3%)	0 (0%)	1 (1%)	6 (5%)	2 (3%)
Domain: Death	9 (3%)	0 (0%)	1 (1%)	6 (5%)	2 (3%)
Death	9 (3%)	0 (0%)	1 (1%)	6 (5%)	2 (3%)
5. CORE AREA: PATHOPHYSIOLOGICAL MANIFESTATIONS	281 (82%)	71 (85%)	53 (79%)	102 (85%)	55 (76%)
Domain: Monitoring of Disease-specific Biomarkers and Surrogate Outcomes	234 (68%)	57 (68%)	42 (63%)	86 (72%)	49 (68%)
Frequency of phenylalanine or tyrosine monitoring	9 (3%)	2 (2%)	0 (0%)	6 (5%)	1 (1%)
Phenylalanine concentration in the blood and other tissues^1^	228 (66%)	56 (67%)	41 (61%)	83 (69%)	48 (67%)
Tyrosine concentration in the blood^7^	39 (11%)	9 (11%)	5 (7%)	18 (15%)	7 (10%)
Phenylalanine to tyrosine ratio	13 (4%)	1 (1%)	2 (3%)	7 (6%)	3 (4%)
Phenylalanine metabolism and kinetics	4 (1%)	2 (2%)	0 (0%)	1 (1%)	1 (1%)
Large neutral amino acid (LNAA) concentration in the blood	1 (0%)	0 (0%)	0 (0%)	1 (1%)	0 (0%)
Micronutrient deficiency	2 (1%)	0 (0%)	0 (0%)	0 (0%)	2 (3%)
BH4 malabsorption	1 (0%)	0 (0%)	1 (1%)	0 (0%)	0 (0%)
Pharmacokinetics of sapropterin	2 (1%)	0 (0%)	1 (1%)	0 (0%)	1 (1%)
Domain: Monitoring of Non Disease-Specific Biomarkers and Surrogate Outcomes	151 (44%)	37 (44%)	28 (42%)	55 (46%)	31 (43%)
Bone health	29 (8%)	4 (5%)	3 (4%)	14 (12%)	8 (11%)
Blood health	27 (8%)	4 (5%)	3 (4%)	13 (11%)	7 (10%)
Neurological health – Clinical symptoms and diagnoses	20 (6%)	3 (4%)	4 (6%)	9 (8%)	4 (6%)
Neurological health – Biomarkers and surrogate outcomes^8*^	35 (10%)	12 (14%)	5 (7%)	12 (10%)	6 (8%)
Neurotransmitters	7 (2%)	3 (4%)	2 (3%)	1 (1%)	1 (1%)
Liver health	6 (2%)	0 (0%)	0 (0%)	4 (3%)	2 (3%)
Kidney health	9 (3%)	2 (2%)	0 (0%)	4 (3%)	3 (4%)
Metabolic syndrome/energy metabolism^3^	52 (15%)	12 (14%)	10 (15%)	20 (17%)	10 (14%)
Biomarkers of protein synthesis	22 (6%)	3 (4%)	4 (6%)	11 (9%)	4 (6%)
Biomarkers of vitamins and trace minerals^4^	45 (13%)	9 (11%)	5 (7%)	19 (16%)	12 (17%)
Concentration of amino acids other than Phe/Tyr in the blood	28 (8%)	4 (5%)	3 (4%)	12 (10%)	9 (13%)
Biomarkers of antioxidant status, oxidative stress and inflammation	20 (6%)	4 (5%)	7 (10%)	6 (5%)	3 (4%)
Neonatal complications	2 (1%)	0 (0%)	0 (0%)	1 (1%)	1 (1%)
Dermatological health	6 (2%)	0 (0%)	1 (1%)	1 (1%)	4 (6%)
Gastrointestinal health	8 (2%)	0 (0%)	0 (0%)	4 (3%)	4 (6%)
Immune system disorders	1 (0%)	0 (0%)	0 (0%)	0 (0%)	1 (1%)
Infections	4 (1%)	0 (0%)	0 (0%)	1 (1%)	3 (4%)
Musculoskeletal health	9 (3%)	0 (0%)	0 (0%)	7 (6%)	2 (3%)
Respiratory health	5 (1%)	0 (0%)	0 (0%)	2 (2%)	3 (4%)
Dental and oral health	3 (1%)	1 (1%)	0 (0%)	1 (1%)	1 (1%)
Ear health	1 (0%)	0 (0%)	0 (0%)	0 (0%)	1 (1%)
Eye health	2 (1%)	0 (0%)	0 (0%)	1 (1%)	1 (1%)

^1–10^indicates top ten most reported or discussed unique outcomes, ties indicated with an asterisks; ^p^ indicates neuro-psychological outcomes

Phenylalanine concentration in the blood and other tissues was the most common unique outcome reported or discussed among the articles included in our review (228/343 articles, 66%). The next most frequently reported outcomes were cognition and intelligence/IQ (82/343 articles, 24%) and metabolic syndrome/energy metabolism (52/343 articles, 15%). Among the 10 most frequently reported unique outcomes, five were within the pathophysiological manifestations core area (two in the monitoring of disease-specific biomarkers and surrogate outcomes domain, three in the monitoring of non disease-specific biomarkers and surrogate outcomes domain), four were within the growth and development core area (three from the physical growth and anthropometry domain, one from cognition and development), and one from the life impact core area (from the disease management and feeding behaviour domain). Sixteen percent of outcomes (16/97 outcomes) were reported by a single study.

#### Changes over time to reported outcomes for PKU

We observed changes over time in the frequency of reporting for some unique outcomes identified in the reviewed studies related to PKU (Table 3). For example, within the growth and development core area, there was an increase in articles reporting unique outcomes in the domain of physical growth and anthropometry from the period between 2000 and 2004 (6/84 or 7% of articles) to the period after 2014 (18/72 or 25% of articles). Within this domain there was increased reporting of body mass index (from 4/84 or 5% of articles from 2000 to 2004 to 14/72 or 19% of articles after 2014), and its components, i.e., height/length (from 4/84 or 5% in 2000–2004 to 13/72 or 18% of articles after 2014), and weight (from 2/84 or 2% of articles from 2000 to 2004 to 11/72 or 15% of articles after 2014). There was a decrease in reporting of outcomes within the domain of cognition and development (from 34/84 or 40% of articles from 2000 to 2004 to 19/72 or 26% of articles after 2014). The frequency of articles reporting outcomes in the life impact core area increased over time (from 28/84 or 33% of articles from 2000 to 2004 to 43/72 or 60% of articles after 2014), with the largest increase seen in the domain of disease management and feeding behaviour (from 19/84 or 23% of articles in 2000–2004 to 32/72 or 44% of articles after 2014).

#### Outcome measurement instruments for selected PKU outcomes

We summarized data for outcome measurement instruments associated with neuro-psychological outcomes and/or outcomes that were typically measured using self- or parent-reported questionnaires. Among 131 articles that measured such outcomes for PKU, we identified 88 outcome measurement instruments associated with 17 unique outcomes (see Additional file 5). The three unique outcomes with the most diverse sets of instruments were in the cognition and development domain. For the outcome, cognition and intelligence/IQ, there were 39 different instruments reported among 82 articles, with the Wechsler Intelligence Scales family of measurement instruments [23] being the most frequently specified (33/82 articles, 40%). Executive functioning was measured using 25 different instruments across 32 articles with the Behaviour Rating Inventory of Executive Function (BRIEF) [24] being the most frequently specified (10/32 articles, 31%). For sensorimotor and motor functioning, there were 17 different instruments across 32 articles with the Bayley Scales of Infant and Toddler Development [25] being the most frequently reported (4/32 articles, 13%). There were no notable trends in the reporting of outcome measurement instruments over time.

## Discussion

We reviewed pediatric literature related to MCAD deficiency and PKU to identify the scope of reported and recommended outcomes. Similar to reviews of outcomes reporting in other clinical areas [26–28], we found substantial diversity of outcomes reported across the five core areas of outcome measurement for both diseases. Notably, almost a third of outcomes for MCAD deficiency and over 15% for PKU were reported in only a single study. With little overlap of outcomes across studies, this suggests limited potential for combining or comparing results across published studies for these diseases and highlights the potential value of developing COSs for MCAD deficiency and PKU.

For MCAD deficiency, there was relatively equal representation of each of the five core areas: approximately half of published studies (46–56%) incorporated outcomes within each core area with the exception of resource use (35%). The most frequently reported outcomes for MCAD deficiency were focused on the risk of life-threatening consequences and manifestations of the disease. This emphasis on mortality-related outcomes is seen in COSs for other potentially life-threatening conditions like post-partum haemorrhage [29] and fetal growth restriction [30]. By contrast, for PKU, there was a dominance of the pathophysiological manifestations core area, similar to that seen in a review of outcomes for type II diabetes [28], and for children with feeding tubes and neurological impairments [22]. The focus on pathophysiological manifestations for PKU reflects frequent reporting of the specific outcome blood phenylalanine concentration. Blood phenylalanine is a well-established surrogate indicator of clinical symptoms for PKU and has been used as a marker of treatment adherence in treatment guidelines and as a clinical trial outcome [13–15]. Industry-sponsored studies may also be more likely to incorporate short-term and surrogate outcomes in evaluating treatments for rare diseases [4], which could contribute to the prominence of pathophysiological end-points in studies of PKU. However, the relatively small number of articles on PKU that reported patient-oriented outcomes (as compared with blood phenylalanine) is potentially of concern.

Specifically, patient-oriented outcomes that reflect the lived experience of patients and their caregivers have emerged as a key priority for evaluative studies in the field of rare diseases [4]. The life impact core area, which covers many such outcomes, appeared relatively well-represented in articles reporting on MCAD deficiency, although most individual outcomes within this core area were themselves reported only once or twice, perhaps reflecting a lack of consensus on which aspects of life impacts are of highest priority for measurement. In the PKU literature, the life impact core area was less commonly represented but, similar to MCAD deficiency, it was by far the most diverse core area, with 44 unique outcomes. This suggests a need to work directly with patients and their family members to identify which patient-oriented outcomes are most meaningful to measure in future research for both diseases. Furthermore, there was diversity in the specific outcome measurement instruments reported for many of the patient- or caregiver-reported outcomes for both diseases. There are very few disease-specific questionnaires for MCAD deficiency and PKU, likely because the small patient populations make the development and validation of such outcome measurement instruments challenging [6]. Thus, in addition to understanding which outcomes are most highly prioritized, there is a need to select the generic instruments (or develop disease-specific instruments) that best capture the life impact of MCAD deficiency and PKU for patients and their families.

This review has several strengths. The study followed a published protocol written in collaboration with patient partners, and established methodology as reported in the PRISMA statement and COMET handbook [7, 18]. Our search for relevant articles was extensive, covering electronic databases of peer reviewed literature, supplemented by a grey literature search guided by Grey Matters [20], additional search strategies for long-term follow-up initiatives of newborn screening, and a search of the COMET database for pediatric COSs [8]. Through data extraction and synthesis we created a comprehensive list of all of the outcomes reported or recommended in the MCAD deficiency and PKU literature that can be used to support the development of COSs for these diseases. Our review also has limitations. Despite our comprehensive search strategy, the non-specific nature of index terms for newborn screening long-term follow-up initiatives made searching for studies in that area challenging and we may have missed relevant published articles. Due to the size of the PKU literature we opted to extract literature from the year 2000 onward and we may have missed unique outcomes that were reported only before that date. We only included studies published in English for practical reasons and may have missed important literature published in other languages. We also originally planned to summarize outcomes by the age of the children studied and we considered a summary that accounted for disease severity, but we were unable to extract these variables consistently due to incomplete reporting of sample characteristics. Similarly, we did not report outcomes by other study characteristics such as follow-up time for longitudinal studies and we did not collect information about how often outcomes were collected in studies where repeat measurements would have been possible.

Our findings suggest that evaluative studies of interventions for MCAD deficiency and PKU would benefit from COSs given the multitude of outcomes in the literature. The 83 MCAD deficiency and 97 PKU outcomes that we identified constitute a list of candidate core outcomes for the next stage of COS development, which involves a consensus process to narrow down the list to a small number of outcomes that are of highest priority for collection in future studies [7]. In order to make final recommendations about outcomes and also outcome measurement tools, the consensus process must involve multiple stakeholders, including patients and family members, to ensure that future evaluative research is patient-oriented and focused on meaningful outcomes. This is particularly important given the large number of outcomes and outcome measurement instruments that we identified in the life impact core area for both diseases, and the focus on pathophysiological rather than patient-oriented outcomes in the PKU literature.

## Conclusions

Substantial heterogeneity exists in the outcomes reported in the MCAD deficiency and PKU literature and a diversity of outcome measurement instruments was used to measure many of these outcomes. This lack of consistency impedes comparisons among studies and limits the potential for data synthesis, leading to inefficient use of limited resources available for evaluating the effectiveness of new and existing interventions. Our findings suggest that future studies of the effectiveness and comparative effectiveness of interventions for pediatric MCAD deficiency and PKU would benefit from disease-specific COSs.

## Supplementary information

**Additional file 1.** Microsoft Word document (.docx). Title: Supplemental Materials and Methods: Description: Includes PRISMA checklist, search strategies, screening forms, and data extraction fields

**Additional file 2.** Microsoft Word document (.docx). Title: List of eligible studies excluded from data synthesis. Description: A table listing the references for eligible PKU and COMET database articles that were excluded from data synthesis

**Additional file 3.** Microsoft Excel document (.xlsx). Title: Review database. Description: Database of the studies included in data synthesis

**Additional file 4.** Microsoft Word document (.docx). Title: Measurement instruments for typically self-reported or neuropsychological MCAD deficiency outcomes. Description: Includes frequency data and references for listed measurement instruments

**Additional file 5.** Microsoft Word document (.docx). Title: Measurement instruments for typically self-reported or neuropsychological PKU outcomes. Description: Includes frequency data and references for listed measurement instruments

## Data Availability

The dataset supporting the conclusions of this article is included within the article and its additional files.

## Abbreviations

ADHD: Attention-Deficit Hyperactivity Disorder
ASD: Autism Spectrum Disorder
CADTH: Canadian Agency for Drugs and Technologies in Health
COMET: Core Outcome Measures in Effectiveness Trials
COS: Core Outcome Set
IMD: Inherited Metabolic Disease
MCAD: Medium Chain Acyl-coA Dehydrogenase
OMERACT: Outcome Measures in Rheumatology
PKU: Phenylketonuria
PRISMA: Preferred Reporting Items for Systematic Reviews and Meta-Analyses
