# Supplementary material for: Outcomes in pediatric studies of medium-chain acyl-coA dehydrogenase (MCAD) deficiency and phenylketonuria (PKU): a review
Source: Orphanet J Rare Dis. 2020 Jan 14;15:12. doi: 10.1186/s13023-019-1276-1 (PMC6961328; doi:10.1186/s13023-019-1276-1)
Supplement: Supplementary file 1 — Additional file 1. Microsoft Word document (.docx). Title: Supplemental Materials and Methods: Description: Includes PRISMA checklist, search strategies, screening forms, and data extraction fields [file 13023_2019_1276_MOESM1_ESM.docx]

**Additional File 1.** Supplemental Materials and Methods.

**1.1: PRISMA Checklist**

| **Section/topic** | **#** | **Checklist item** | **Reported on page #** |
| --- | --- | --- | --- |
| **TITLE** | | |  |
| Title | 1 | Identify the report as a systematic review, meta-analysis, or both. | 1 |
| **ABSTRACT** | | |  |
| Structured summary | 2 | Provide a structured summary including, as applicable: background; objectives; data sources; study eligibility criteria, participants, and interventions; study appraisal and synthesis methods; results; limitations; conclusions and implications of key findings; systematic review registration number. | 2-3 |
| **INTRODUCTION** | | |  |
| Rationale | 3 | Describe the rationale for the review in the context of what is already known. | 4-5 |
| Objectives | 4 | Provide an explicit statement of questions being addressed with reference to participants, interventions, comparisons, outcomes, and study design (PICOS). | 5 |
| **METHODS** | | |  |
| Protocol and registration | 5 | Indicate if a review protocol exists, if and where it can be accessed (e.g., Web address), and, if available, provide registration information including registration number. | 5 |
| Eligibility criteria | 6 | Specify study characteristics (e.g., PICOS, length of follow-up) and report characteristics (e.g., years considered, language, publication status) used as criteria for eligibility, giving rationale. | 6-7 |
| Information sources | 7 | Describe all information sources (e.g., databases with dates of coverage, contact with study authors to identify additional studies) in the search and date last searched. | 6 |
| Search | 8 | Present full electronic search strategy for at least one database, including any limits used, such that it could be repeated. | Additional file 1 |
| Study selection | 9 | State the process for selecting studies (i.e., screening, eligibility, included in systematic review, and, if applicable, included in the meta-analysis). | 7-8 |
| Data collection process | 10 | Describe method of data extraction from reports (e.g., piloted forms, independently, in duplicate) and any processes for obtaining and confirming data from investigators. | 8 |
| Data items | 11 | List and define all variables for which data were sought (e.g., PICOS, funding sources) and any assumptions and simplifications made. | 8 |
| Risk of bias in individual studies | 12 | Describe methods used for assessing risk of bias of individual studies (including specification of whether this was done at the study or outcome level), and how this information is to be used in any data synthesis. | N/A |
| Summary measures | 13 | State the principal summary measures (e.g., risk ratio, difference in means). | N/A |
| Synthesis of results | 14 | Describe the methods of handling data and combining results of studies, if done, including measures of consistency (e.g., I^2^) for each meta-analysis. | 8 |

| **Section/topic** | **#** | **Checklist item** | **Reported on page #** |
| --- | --- | --- | --- |
| Risk of bias across studies | 15 | Specify any assessment of risk of bias that may affect the cumulative evidence (e.g., publication bias, selective reporting within studies). | N/A |
| Additional analyses | 16 | Describe methods of additional analyses (e.g., sensitivity or subgroup analyses, meta-regression), if done, indicating which were pre-specified. | N/A |
| **RESULTS** | | |  |
| Study selection | 17 | Give numbers of studies screened, assessed for eligibility, and included in the review, with reasons for exclusions at each stage, ideally with a flow diagram. | 9 |
| Study characteristics | 18 | For each study, present characteristics for which data were extracted (e.g., study size, PICOS, follow-up period) and provide the citations. | 9-10, 13 |
| Risk of bias within studies | 19 | Present data on risk of bias of each study and, if available, any outcome level assessment (see item 12). | N/A |
| Results of individual studies | 20 | For all outcomes considered (benefits or harms), present, for each study: (a) simple summary data for each intervention group (b) effect estimates and confidence intervals, ideally with a forest plot. | N/A |
| Synthesis of results | 21 | Present results of each meta-analysis done, including confidence intervals and measures of consistency. | 9-15 |
| Risk of bias across studies | 22 | Present results of any assessment of risk of bias across studies (see Item 15). | N/A |
| Additional analysis | 23 | Give results of additional analyses, if done (e.g., sensitivity or subgroup analyses, meta-regression [see Item 16]). | N/A |
| **DISCUSSION** | | |  |
| Summary of evidence | 24 | Summarize the main findings including the strength of evidence for each main outcome; consider their relevance to key groups (e.g., healthcare providers, users, and policy makers). | 15-16 |
| Limitations | 25 | Discuss limitations at study and outcome level (e.g., risk of bias), and at review-level (e.g., incomplete retrieval of identified research, reporting bias). | 17 |
| Conclusions | 26 | Provide a general interpretation of the results in the context of other evidence, and implications for future research. | 17-18 |
| **FUNDING** | | |  |
| Funding | 27 | Describe sources of funding for the systematic review and other support (e.g., supply of data); role of funders for the systematic review. | 20 |

*From:*  Moher D, Liberati A, Tetzlaff J, Altman DG, The PRISMA Group (2009). Preferred Reporting Items for Systematic Reviews and Meta-Analyses: The PRISMA Statement. PLoS Med 6(7): e1000097. doi:10.1371/journal.pmed1000097

For more information, visit: **www.prisma-statement.org**.

**1.2: Final Search Strategies**

**(i) MCAD deficiency – Strategies**

Final Strategies

2017 Jul 20

Ovid Multifile

Database: Embase Classic+Embase <1947 to 2017 July 19>, Ovid MEDLINE(R) Epub Ahead of Print, In-Process & Other Non-Indexed Citations, Ovid MEDLINE(R) Daily and Ovid MEDLINE(R) <1946 to Present>

Search Strategy:

--------------------------------------------------------------------------------

1 Acyl-CoA Dehydrogenase/df [Deficiency] (216)

2 (MCAD or MCADD).tw,kw. (1727)

3 medium chain acyl coenzyme A dehydrogenase deficien*.tw,kw. (126)

4 medium chain acyl-CoA dehydrogenase deficien*.tw,kw. (812)

5 medium chain acyl dehydrogenase deficien*.tw,kw. (0)

6 MCACA-dehydrogenase deficien*.tw,kw. (0)

7 Octanoyl-CoA dehydrogenase deficien*.tw,kw. (0)

8 Octanoyl-coenzyme A dehydrogenase deficien*.tw,kw. (0)

9 or/1-8 (2258)

10 exp Animals/ not (exp Animals/ and Humans/) (15364299)

11 9 not 10 [ANIMAL-ONLY REMOVED] (1575)

12 11 use ppez [MEDLINE RECORDS] (811)

13 medium chain acyl coenzyme A dehydrogenase deficiency/ (243)

14 (MCAD or MCADD).tw,kw. (1727)

15 medium chain acyl coenzyme A dehydrogenase deficien*.tw,kw. (126)

16 medium chain acyl-CoA dehydrogenase deficien*.tw,kw. (812)

17 medium chain acyl dehydrogenase deficien*.tw,kw. (0)

18 MCACA-dehydrogenase deficien*.tw,kw. (0)

19 Octanoyl-CoA dehydrogenase deficien*.tw,kw. (0)

20 Octanoyl-coenzyme A dehydrogenase deficien*.tw,kw. (0)

21 or/13-20 (2267)

22 exp animal experimentation/ or exp animal model/ or exp animal experiment/ or nonhuman/ or exp vertebrate/ (45907618)

23 exp human/ or exp human experimentation/ or exp human experiment/ (35927904)

24 22 not 23 (9981417)

25 21 not 24 [ANIMAL-ONLY REMOVED] (1884)

26 25 use emczd [EMBASE RECORDS] (1128)

27 12 or 26 [BOTH DATABASES] (1939)

28 limit 27 to english language (1840)

29 limit 28 to yr="1990-CURRENT" (1738)

30 remove duplicates from 29 (1156) [TOTAL UNIQUE RECORDS]

31 30 use ppez [MEDLINE UNIQUE RECORDS] (704)

32 30 use emczd [EMBASE UNIQUE RECORDS] (452)

***************************

Cochrane Library

Search Name: MCADD - Outcomes

Date Run: 20/07/17 18:47:10.437

Description: Final - 2017 Jul 20

ID Search Hits

#1 [mh "Acyl-CoA Dehydrogenase"/DF] 6

#2 (MCAD or MCADD):ti,ab,kw 8

#3 "medium chain acyl coenzyme A dehydrogenase" next deficien*:ti,ab,kw 6

#4 "medium chain acyl-CoA dehydrogenase" next deficien*:ti,ab,kw 10

#5 "medium chain acyl dehydrogenase" next deficien*:ti,ab,kw 0

#6 "MCACA-dehydrogenase" next deficien*:ti,ab,kw 0

#7 "octanoyl-CoA dehydrogenase" next deficien*:ti,ab,kw 0

#8 "octanoyl-coenzyme A dehydrogenase" next deficien*:ti,ab,kw 0

#9 {or #1-#8} Publication Year from 1990 to 2017 21

DSR – 1

DARE – 1

CENTRAL – 11

Methods – 1

HTA - 1

NHS EED - 6

**(ii) PKU –Strategies**

Final Strategies

2017 Jul 20

Ovid Multifile

Database: Embase Classic+Embase <1947 to 2017 July 19>, Ovid MEDLINE(R) Epub Ahead of Print, In-Process & Other Non-Indexed Citations, Ovid MEDLINE(R) Daily and Ovid MEDLINE(R) <1946 to Present>

Search Strategy:

--------------------------------------------------------------------------------

1 exp Phenylketonurias/ (16762)

2 phenylketonuria*.tw,kw. (13086)

3 PKU.tw,kw. (6470)

4 ((folling* or foelling*) adj disease).tw,kw. (73)

5 BH4 deficien*.tw,kw. (450)

6 dihydropteridine reductase deficien*.tw,kw. (238)

7 DHPR deficien*.tw,kw. (171)

8 phenylalanine hydroxylase deficien*.tw,kw. (337)

9 PAH deficien*.tw,kw. (331)

10 oligophrenia phenylpyruvica.tw,kw. (24)

11 phenylpyruvic oligophrenia.tw,kw. (362)

12 quinoid dihydropteridine reductase deficien*.tw,kw. (0)

13 QDPR deficien*.tw,kw. (5)

14 Hyperphenylalanin?emia*.tw,kw. (2883)

15 HPABH4C.tw,kw. (0)

16 tetrahydrobiopterin deficien*.tw,kw. (337)

17 mckusick 26160.tw,kw. (5)

18 or/1-17 (19990)

19 exp Animals/ not (exp Animals/ and Humans/) (15364299)

20 18 not 19 (16858)

21 exp Phenylketonurias/dh, dt, th [PKU - Therapies] (2022)

22 (controlled clinical trial or randomized controlled trial or pragmatic clinical trial).pt. (559492)

23 clinical trials as topic.sh. (187520)

24 exp Randomized Controlled Trials as Topic/ (248867)

25 (randomi#ed or randomi#ation* or randomly or RCT$1 or placebo*).tw,kw. (1957159)

26 ((singl* or doubl* or trebl* or tripl*) adj (mask* or blind* or dumm*)).tw,kw. (366730)

27 trial.ti. (419573)

28 or/22-27 (2534112)

29 20 and 28 [RCTs] (324)

30 controlled clinical trial.pt. (94452)

31 Controlled Clinical Trial/ or Controlled Clinical Trials as Topic/ (548068)

32 (control* adj2 trial*).tw,kw. (493512)

33 Non-Randomized Controlled Trials as Topic/ (9155)

34 (nonrandom* or non-random* or quasi-random* or quasi-experiment*).tw,kw. (101310)

35 (nRCT or nRCTs or non-RCT?).tw,kw. (1394)

36 Controlled Before-After Studies/ (206875)

37 (control* adj3 ("before and after" or "before after")).tw,kw. (8188)

38 Interrupted Time Series Analysis/ (198788)

39 time series.tw,kw. (49919)

40 (pre- adj3 post-).tw,kw. (170661)

41 (pretest adj3 posttest).tw,kw. (9055)

42 Historically Controlled Study/ (217165)

43 (control* adj2 stud$3).tw,kw. (461757)

44 Control Groups/ (124743)

45 (control* adj2 group$1).tw,kw. (1015499)

46 trial.ti. (419573)

47 or/30-46 (2889255)

48 20 and 47 [non-RCTs] (653)

49 exp Cohort Studies/ (2021662)

50 cohort?.tw,kw. (1119011)

51 Retrospective Studies/ (985850)

52 (longitudinal or prospective or retrospective).tw,kw. (2491463)

53 ((followup or follow-up) adj (study or studies)).tw,kw. (109916)

54 Observational study.pt. (39817)

55 (observation$2 adj (study or studies)).tw,kw. (191860)

56 ((population or population-based) adj (study or studies or analys#s)).tw,kw. (42520)

57 ((multidimensional or multi-dimensional) adj (study or studies)).tw,kw. (236)

58 Comparative Study.pt. (1821653)

59 ((comparative or comparison) adj (study or studies)).tw,kw. (225863)

60 exp Case-Control Studies/ (1024656)

61 ((case-control* or case-based or case-comparison) adj (study or studies)).tw,kw. (201873)

62 or/49-61 (6395533)

63 20 and 62 [Observational Studies] (1459)

64 29 or 48 or 63 [ALL STUDY DESIGNS] (2105)

65 limit 20 to systematic reviews [Limit not valid in Embase; records were retained] (9125)

66 meta analysis.pt. (83207)

67 exp meta-analysis as topic/ (52290)

68 (meta-analy* or metanaly* or metaanaly* or met analy* or integrative research or integrative review* or integrative overview* or research integration or research overview* or collaborative review*).tw,kw. (278698)

69 (systematic review* or systematic overview* or evidence-based review* or evidence-based overview* or (evidence adj3 (review* or overview*)) or meta-review* or meta-overview* or meta-synthes* or "review of reviews" or technology assessment* or HTA or HTAs).tw,kw. (326719)

70 exp Technology assessment, biomedical/ (22438)

71 (cochrane or health technology assessment or evidence report).jw. (33530)

72 (network adj (MA or MAs)).tw,kw. (15)

73 (NMA or NMAs).tw,kw. (3619)

74 indirect comparison?.tw,kw. (3631)

75 (indirect treatment* adj1 comparison?).tw,kw. (472)

76 (mixed treatment* adj1 comparison?).tw,kw. (1151)

77 (multiple treatment* adj1 comparison?).tw,kw. (200)

78 (multi-treatment* adj1 comparison?).tw,kw. (3)

79 simultaneous comparison?.tw,kw. (904)

80 mixed comparison?.tw,kw. (35)

81 or/66-80 (588476)

82 20 and 81 (105)

83 65 or 82 [REVIEWS] (9142)

84 21 or 64 or 83 [ALL THERAPIES & STUDY DESIGNS (INCLUDING REVIEWS)] (11838)

85 limit 84 to english language (9333)

86 85 use ppez [MEDLINE RECORDS] (2325)

87 phenylketonuria/ (16660)

88 phenylketonuria*.tw,kw. (13086)

89 PKU.tw,kw. (6470)

90 ((folling* or foelling*) adj disease).tw,kw. (73)

91 BH4 deficien*.tw,kw. (450)

92 dihydropteridine reductase deficien*.tw,kw. (238)

93 DHPR deficien*.tw,kw. (171)

94 phenylalanine hydroxylase deficien*.tw,kw. (337)

95 PAH deficien*.tw,kw. (331)

96 oligophrenia phenylpyruvica.tw,kw. (24)

97 phenylpyruvic oligophrenia.tw,kw. (362)

98 quinoid dihydropteridine reductase deficien*.tw,kw. (0)

99 QDPR deficien*.tw,kw. (5)

100 Hyperphenylalanin?emia*.tw,kw. (2883)

101 HPABH4C.tw,kw. (0)

102 tetrahydrobiopterin deficien*.tw,kw. (337)

103 mckusick 26160.tw,kw. (5)

104 or/87-103 (19984)

105 exp animal experimentation/ or exp animal model/ or exp animal experiment/ or nonhuman/ or exp vertebrate/ (45907618)

106 exp human/ or exp human experimentation/ or exp human experiment/ (35927904)

107 105 not 106 (9981417)

108 104 not 107 [ANIMAL-ONLY REMOVED] (18765)

109 phenylketonuria/dm, dt, th [PKU - THERAPIES] (1409)

110 randomized controlled trial/ or controlled clinical trial/ (1186929)

111 exp "clinical trial (topic)"/ (245514)

112 (randomi#ed or randomi#ation* or randomly or RCT$1 or placebo*).tw,kw. (1957159)

113 ((singl* or doubl* or trebl* or tripl*) adj (mask* or blind* or dumm*)).tw,kw. (366730)

114 trial.ti. (419573)

115 or/110-114 (2672482)

116 108 and 115 [RCTS] (468)

117 controlled clinical trial/ (535322)

118 "controlled clinical trial (topic)"/ (8974)

119 (control* adj2 trial*).tw,kw. (493512)

120 (nonrandom* or non-random* or quasi-random* or quasi-experiment*).tw,kw. (101310)

121 (nRCT or nRCTs or non-RCT?).tw,kw. (1394)

122 (control* adj3 ("before and after" or "before after")).tw,kw. (8188)

123 time series analysis/ (19844)

124 time series.tw,kw. (49919)

125 pretest posttest control group design/ (311)

126 (pre- adj3 post-).tw,kw. (170661)

127 (pretest adj3 posttest).tw,kw. (9055)

128 controlled study/ (5611332)

129 (control* adj2 stud$3).tw,kw. (461757)

130 control group/ (124743)

131 (control* adj2 group$1).tw,kw. (1015499)

132 trial.ti. (419573)

133 or/117-132 (7391292)

134 108 and 133 [NON-RCTs] (1573)

135 cohort analysis/ (524693)

136 cohort?.tw,kw. (1119011)

137 retrospective study/ (1222475)

138 longitudinal study/ (215889)

139 prospective study/ (858625)

140 (longitudinal or prospective or retrospective).tw,kw. (2491463)

141 follow up/ (1212967)

142 ((followup or follow-up) adj (study or studies)).tw,kw. (109916)

143 observational study/ (161155)

144 (observation$2 adj (study or studies)).tw,kw. (191860)

145 population research/ (86852)

146 ((population or population-based) adj (study or studies or analys#s)).tw,kw. (42520)

147 ((multidimensional or multi-dimensional) adj (study or studies)).tw,kw. (236)

148 exp comparative study/ (3052001)

149 ((comparative or comparison) adj (study or studies)).tw,kw. (225863)

150 exp case control study/ (1024656)

151 ((case-control* or case-based or case-comparison) adj (study or studies)).tw,kw. (201873)

152 or/135-151 (8064914)

153 108 and 152 [OBSERVATIONAL STUDIES] (2171)

154 116 or 134 or 153 [ALL STUDY DESIGNS] (3480)

155 meta-analysis/ (214055)

156 "systematic review"/ (144425)

157 "meta analysis (topic)"/ (35936)

158 (meta-analy* or metanaly* or metaanaly* or met analy* or integrative research or integrative review* or integrative overview* or research integration or research overview* or collaborative review*).tw,kw. (278698)

159 (systematic review* or systematic overview* or evidence-based review* or evidence-based overview* or (evidence adj3 (review* or overview*)) or meta-review* or meta-overview* or meta-synthes* or "review of reviews" or technology assessment* or HTA or HTAs).tw,kw. (326719)

160 biomedical technology assessment/ (21317)

161 (cochrane or health technology assessment or evidence report).jw. (33530)

162 (network adj (MA or MAs)).tw,kw. (15)

163 (NMA or NMAs).tw,kw. (3619)

164 indirect comparison?.tw,kw. (3631)

165 (indirect treatment* adj1 comparison?).tw,kw. (472)

166 (mixed treatment* adj1 comparison?).tw,kw. (1151)

167 (multiple treatment* adj1 comparison?).tw,kw. (200)

168 (multi-treatment* adj1 comparison?).tw,kw. (3)

169 simultaneous comparison?.tw,kw. (904)

170 mixed comparison?.tw,kw. (35)

171 or/155-170 (633419)

172 108 and 171 [REVIEWS] (165)

173 109 or 154 or 172 [ALL THERAPIES & STUDY DESIGNS (INCLUDING REVIEWS)] (4499)

174 limit 173 to english language (3998)

175 174 use emczd [EMBASE RECORDS] (2936)

176 86 or 175 [BOTH DATABASES] (5261)

177 limit 176 to yr="1990-current" (4090)

178 remove duplicates from 177 (2928) [TOTAL UNIQUE RECORDS]

179 178 use ppez [MEDLINE UNIQUE RECORDS] (1507)

180 178 use emczd [EMBASE UNIQUE RECORDS] (1421)

***************************

Cochrane Library

Search Name: PKU - Outcomes

Date Run: 20/07/17 19:06:13.719

Description: Final - 2017 Jul 20

ID Search Hits

#1 [mh Phenylketonurias] 108

#2 phenylketonuria*:ti,ab,kw 242

#3 PKU:ti,ab,kw 167

#4 ((folling* or foelling*) next disease):ti,ab,kw 0

#5 BH4 next deficien*:ti,ab,kw 3

#6 "dihydropteridine reductase" next deficien*:ti,ab,kw 1

#7 DHPR next deficien*:ti,ab,kw 0

#8 "phenylalanine hydroxylase" next deficien*:ti,ab,kw 5

#9 PAH next deficien*:ti,ab,kw 4

#10 "oligophrenia phenylpyruvica":ti,ab,kw 0

#11 "phenylpyruvic oligophrenia":ti,ab,kw 0

#12 "quinoid dihydropteridine reductase" next deficien*:ti,ab,kw 0

#13 QDPR next deficien*:ti,ab,kw 0

#14 (hyperphenylalaninemia* or hyperphenylalaninaemia*):ti,ab,kw 31

#15 HPABH4C:ti,ab,kw 0

#16 tetrahydrobiopterin next deficien*:ti,ab,kw 1

#17 "mckusick 26160":ti,ab,kw 2

#18 {or #1-#17} 289

#19 [mh Phenylketonurias/DH,DT,TH] 76

#20 #18 or #19 Publication Year from 1990 to 2017 288

DSR – 6 [Reviews]

DARE – 5 [Reviews]

Trials – 261 [RCTs]

Methods – 2 *[Do Not Download]*

HTA – 9 [Reviews]

NHS EED – 5 *[Do Not Download]*

**(iii) IMD Outcomes Searches –Strategies**

Final Strategy

2017 Jul 20

Ovid Multifile

Database: Embase Classic+Embase <1947 to 2017 July 19>, Ovid MEDLINE(R) Epub Ahead of Print, In-Process & Other Non-Indexed Citations, Ovid MEDLINE(R) Daily and Ovid MEDLINE(R) <1946 to Present>

Search Strategy:

--------------------------------------------------------------------------------

1 Genetic Diseases, Inborn/ (34920)

2 exp Metabolism, Inborn Errors/ (410246)

3 (((inborn or in born) adj3 (disease? or disorder? or error?)) and metaboli*).tw,kw. (14572)

4 (((familial or genetic or inherited or heritabl* or hereditary) adj3 (disease? or disorder?)) and metaboli*).tw,kw. (22907)

5 or/1-4 (460213)

6 Neonatal Screening/ (23363)

7 ((neonat* or newborn* or infant*) adj3 (screen* or detect*)).tw,kw. (37460)

8 exp Infant, Newborn/ and (screen* or detect*).tw,kw. (116380)

9 or/6-8 (137556)

10 5 and 9 (12909)

11 exp "Outcome Assessment (Health Care)"/ (1306232)

12 (outcome? adj3 (assess* or evaluat* or measur* or study or studying or studied or studies)).tw,kw. (801726)

13 Program Evaluation/ (65189)

14 (program* adj3 (assess* or evaluat*)).tw,kw. (66536)

15 Quality Assurance, Health Care/ (218960)

16 (quality adj3 (assess* or assur*)).tw,kw. (184604)

17 exp Quality Control/ (356586)

18 (quality adj3 control*).tw,kw. (117343)

19 Quality Improvement/ (33488)

20 (quality adj3 improv*).tw,kw. (286405)

21 Evaluation Studies.pt. (234631)

22 (evaluation adj (study or studies)).tw,kw. (12333)

23 Follow-Up Studies/ (1395551)

24 ((followup or follow-up) adj (study or studies)).tw,kw. (109916)

25 ((followup or follow-up) adj3 (long-term or longterm)).tw,kw. (132691)

26 Cost-Benefit Analysis/ (147714)

27 (cost? adj2 benefit?).tw,kw. (43665)

28 Data Collection/ (276070)

29 exp Health Care Surveys/ (43129)

30 Health Surveys/ (218169)

31 ((health or healthcare) adj2 (survey or surveys)).tw,kw. (82745)

32 exp Population Surveillance/ (255016)

33 (surveillance? adj3 population?).tw,kw. (6216)

34 or/11-33 (4969375)

35 10 and 34 (2135)

36 exp Animals/ not (exp Animals/ and Humans/) (15364299)

37 35 not 36 [ANIMAL-ONLY REMOVED] (1643)

38 37 use ppez [MEDLINE RECORDS] (831)

39 exp "inborn error of metabolism"/ (259003)

40 exp metabolic encephalopathy/ (189341)

41 (((inborn or in born) adj3 (disease? or disorder? or error?)) and metaboli*).tw,kw. (14572)

42 (((familial or genetic or inherited or heritabl* or hereditary) adj3 (disease? or disorder?)) and metaboli*).tw,kw. (22907)

43 or/39-42 (396705)

44 newborn screening/ (15647)

45 ((neonat* or newborn* or infant*) adj3 (screen* or detect*)).tw,kw. (37460)

46 newborn/ and (screen* or detect*).tw,kw. (116165)

47 or/44-46 (135648)

48 43 and 47 (11919)

49 exp outcome assessment/ (385693)

50 (outcome? adj3 (assess* or evaluat* or measur* or study or studying or studied or studies)).tw,kw. (801726)

51 exp program evaluation/ (82137)

52 (program* adj3 (assess* or evaluat*)).tw,kw. (66536)

53 health care quality/ (284393)

54 (quality adj3 (assess* or assur*)).tw,kw. (184604)

55 exp quality control/ (356586)

56 (quality adj3 control*).tw,kw. (117343)

57 (quality adj3 improv*).tw,kw. (286405)

58 "evaluation and follow up"/ (4258)

59 evaluation study/ (28626)

60 (evaluation adj (study or studies)).tw,kw. (12333)

61 follow up/ (1212967)

62 ((followup or follow-up) adj (study or studies)).tw,kw. (109916)

63 ((followup or follow-up) adj3 (long-term or longterm)).tw,kw. (132691)

64 "cost benefit analysis"/ (147714)

65 (cost? adj2 benefit?).tw,kw. (43665)

66 health care survey/ (42169)

67 exp health survey/ (674600)

68 ((health or healthcare) adj2 (survey or surveys)).tw,kw. (82745)

69 (surveillance? adj3 population?).tw,kw. (6216)

70 or/49-69 (4139063)

71 48 and 70 (3272)

72 exp animal experimentation/ or exp animal model/ or exp animal experiment/ or nonhuman/ or exp vertebrate/ (45907618)

73 exp human/ or exp human experimentation/ or exp human experiment/ (35927904)

74 72 not 73 (9981417)

75 71 not 74 (3264)

76 75 use emczd [EMBASE RECORDS] (1752)

77 38 or 76 (2583)

78 remove duplicates from 77 (2161)

79 78 use ppez [MEDLINE UNIQUE RECORDS] (810)

80 78 use emczd [EMBASE UNIQUE RECORDS] (1351)

***************************

**(iv) Grey Literature Search (limited to 15 hours)**

Search Date: April 6^th^ – April 17^th^, 2018.

Search Terms (varied depending on source but typically included one or more of the following terms):

- PKU: “PKU”, “phenylketonuria”, “PAH deficiency”, “phenylalanine hydroxylase deficiency”
- MCAD Deficiency: “MCAD”, “MCAD Deficiency”, “MCADD”, “Medium-Chain Acyl CoA dehydrogenase deficiency”

Note: a number of Clinical Practice Guideline and Newborn Screening Program sources did not have a keyword search option. These were manually searched documents related to PKU or MCAD deficiency.

Sources:

| **Clinical Trial Registries** | **Health Technology Assessment Agencies** |
| --- | --- |
| ClinicalTrials.gov | Canadian Agency for Drugs and Technologies in Health |
| Clinical Trials Registry – India | Euroscan Secretariat |
| ISRCTN Registry | INAHTA Secretariat - International Network of Agencies for Health Technology Assessment |
| Thomson CenterWatch |  |
| United Kingdom Clinical Trials gateway |  |
| WHO International Clinical Trials Registry Platform |  |
| **Clinical Practice Guidelines** | **Newborn Screening Programs** |
| Alberta Medical Association | Newborn Screening Translational Research Network |
| American Association for Clinical Chemistry | California Department of Public Health |
| Best Practice Advocacy Centre New Zealand | Colorado Department of Public Health and Environment |
| British Columbia Ministry of Health | Illinois Department of Public Health |
| Canadian Medical Association | Indiana State Department of Health |
| Center for Disease Control - Genomic Testing - Guidelines, Policies, and Recommendations in Genomics | Louisiana Department of Health |
| French National Authority for Health | New England Newborn Screening Program |
| Institute for Clinical Systems Improvement | Michigan Department of Health & Human Services |
| National Guideline Clearinghouse |  |
| National Health and Medical Research Council |  |
| National Institute for Health and Care Excellence |  |
| Ontario Association of Medical Laboratories |  |
| Public Health Agency of Canada |  |
| Registered Nurses' Association of Ontario |  |
| Scottish Intercollegiate Guidelines Network |  |
| The College of Physicians and Surgeons of Ontario |  |
| The Regulation and Quality Improvement Authority |  |
| University of Ottawa School of Rehabilitation Science |  |
| Winnipeg Regional Health Authority |  |

Search Returns: 1023

**(v) Additional Searches for Long-Term Follow-Up Initiatives for Newborn Screening**

Search Date: May 3^rd^, 2018

Key Articles:

Hinton CF, Homer CJ, Thompson AA, et al. A framework for assessing outcomes from newborn screening: on the road to measuring its promise. *Mol Genet Metab*. 2016;118(4):221-229. doi:10.1016/j.ymgme.2016.05.017

Lindner M, Gramer G, Haege G, et al. Efficacy and outcome of expanded newborn screening for metabolic diseases - Report of 10 years from South-West Germany. *Orphanet J Rare Dis*. 2011;6(44):1-10. doi:10.1186/1750-1172-6-44

Search Sources:

- Citation Search: Google Scholar and Web of Science
- Related Articles Search: Google Scholar and PubMed

Search Returns: 811

**(vi) COMET Database Search**

Search Date: April 19^th^, 2018

Search Strategy:

{Target Population - Age{:Minimum age of population: 0:Maximum age of population: 18:}Study Type{:COS for clinical trials or clinical research:COS for practice:COS for registry:COS Patient Reported Outcomes:}}

Search Returns: 334

**1.3: Final Screening Forms**

**(i) Peer-Reviewed Electronic Database Search Screening Forms**

***Level 1: Title/abstract screening***

-Liberal accelerated method: One review to include; two reviewers to exclude.

1. Is this article considered relevant based on our criteria below?

Inclusion Criteria:

Population:

- Children (≤18 years) or mixed population (children and adult) diagnosed with inherited metabolic disease.

Study Design:

- Non-animal studies of PKU and/or MCAD deficiency using any study design.

OR

- Publications focusing on long-term follow-up initiatives related to newborn screening of inherited metabolic diseases (must include PKU and/or MCAD deficiency but these two conditions don’t have to be specifically named if it is a study of newborn screening for inherited metabolic disease generally since there are no newborn screening programs in the world that screen for inherited metabolic diseases but not PKU).

Exclusion Criteria:

Population:

- Adult population (>18 years) exclusively.
- Population not focused on inherited metabolic disease.

Study Design:

- Animal or *in vitro* studies.
- Case reports and case series with less than 5 subjects.
- The focus of the study is solely on evaluating methods of identifying or diagnosing disease.

Other:

- There is no abstract, and the study can be classified as irrelevant with certainty from reading the title.
  - Yes/Unclear (Include)
  - No (Exclude)

***Level 2: Full-text screening***

**-**Two independent reviewers screened articles in duplicate.

1. Is the full text-available?

- Yes (Proceed)
- Study was published before 1990 (Exclude)
- Abstract or conference proceeding only (Exclude)
- Not available (Exclude)

1. Is the article published in English?

- Yes (Proceed)
- No (Exclude)

1. Is it an animal study?

- Yes (Exclude)
- No (Proceed)

1. Is this article about patients diagnosed with inherited metabolic disease(s) (must include PKU and/or MCAD deficiency)?*

* For studies of newborn screening: These two conditions don’t have to be specifically named if it is a study of newborn screening for inherited metabolic disease. Since there are generally no NBS programs in the world that screen for IMDs but not PKU.

- Yes (Proceed)
- No (Exclude)

1. Is the article a study design of interest?

Select ‘Yes’ if it is a primary study

OR

Select ‘Yes’ if the article provides guidance or recommendations for follow-up or long-term monitoring after diagnosis of PKU or MCAD deficiency

- Yes (Proceed)
- No (Exclude)

1. Is the article focused on children (≤18 years) diagnosed with a pediatric condition?

- Yes – Children exclusively (Proceed)
- Yes – Mixed population (Proceed)
- No – Adults exclusively (Exclude)
- No – Age group not specified (Exclude)

1. Are there at least five children in the study?

- Yes (Proceed)
- Study/article does not have any subjects (Proceed)
- No – fewer than five children (Exclude)

1. Does the study report or discuss at least one outcome in children?

*An outcome is “any identified result in a (Sub)Domain that is amenable to change due to the effects of a health intervention” (modified OMERACT definition).

 **A Sub(Domain) is defined by OMERACT as components of Core Areas, which are “aspects of health or a health condition that need to be measured to appropriate assess the effects of a health intervention.”

 ***The outcome being discussed must be presented separately for children in primary studies. For a guidelines and recommendations, children must discussed as a separate subgroup.

- Yes (Include)
- Outcome(s) is/are not reported or discussed separately for children (Exclude)
- No (Exclude)

1. Record Slot Category

- PKU
- MCAD deficiency
- PKU/MCAD deficiency
- Newborn screening

**(ii) Grey Literature Search Screening Forms**

***Level 1: Title/abstract screening***

-Single reviewer included/excluded articles.

1. Does the grey literature document or information source appear to be relevant to pediatric PKU, MCAD deficiency, or the long-term follow-up of newborn screening initiatives?

***Level 2: Full-text screening***

**-**Two independent reviewers screened articles in duplicate.

1. Is the article available in English?

- Yes (Proceed)
- No (Exclude)

1. Is the article a study design of interest?

- Yes – primary study or guideline/recommendation (Proceed)
- No (Exclude)

1. Is the article focused on humans?

- Yes (Proceed)
- No (Exclude)

1. Does the article focus on patients diagnosed with inherited metabolic disease(s) (must include PKU and/or MCAD deficiency)? Note: if focus is just on “newborn screening”, select “Yes.” All newborn screening programs will include PKU/MCAD.

- Yes (Proceed)
- No (Exclude)

1. Is the article focused on children (≤18 years) diagnosed with a pediatric condition or a person acting as their surrogate responder (i.e. parent, caregiver, physician) as the unit of analysis or topic of discussion?

- Yes – Children exclusively (Proceed)
- No – Mixed population (Exclude)
- No – Adults exclusively (Exclude)
- No – Age group not specified (Exclude)

1. Does the article report or discuss at least one outcome in children?

*An outcome is “any identified result in a (Sub)Domain that is amenable to change due to the effects of a health intervention” (modified OMERACT definition).

 **A Sub(Domain) is defined by OMERACT as components of Core Areas, which are “aspects of health or a health condition that need to be measured to appropriate assess the effects of a health intervention.”

 ***The outcome being discussed must be presented separately for children in primary studies. For a guidelines and recommendations, children must discussed as a separate subgroup.

- Yes (Proceed)
- No (Exclude)

1. If a primary study that has been terminated or completed, did the final sample include 5 or more children (≤18 years)?

- Yes (Proceed)
- No (Exclude)

1. Record Slot Category

- PKU
- MCAD deficiency
- PKU/MCAD deficiency
- Newborn screening

**(iii) Additional Searches for Long-Term Follow-Up Initiatives of Newborn Screening**

***Level 1: Title/abstract screening***

-Single reviewer included/excluded articles.

1. Is the article in English?
   - Yes (Proceed)
   - No (Exclude)
2. Does the article appear to be about a long-term follow-up initiative for newborn screening (must include PKU and/or MCAD deficiency although these two diseases do not need to be named specifically, since there are generally no newborn screening programs in the world that screen for inherited metabolic diseases but not PKU)?
   - Yes (Proceed)
   - No (Exclude)
3. Is the article a duplicate of an article already included in the review?
   - Yes (Exclude)
   - No (Include)

***Level 2: Full-text screening***

**-**Two independent reviewers screened articles in duplicate.

Same level 2 screening form as the peer-reviewed electronic database search screening form above.

**(iv) COMET Database Search for Pediatric Projects**

***Level 1: Title/abstract screening***

-Single reviewer included/excluded articles.

1. Is this article considered relevant based on our criteria below?
   - Yes/Unclear (Include)
   - No (Exclude)
   - No Abstract (Include)

| Yes/Unclear | No |
| --- | --- |
| Population:   - Children (≤18 years) or mixed population (children and adult) diagnosed with a pediatric condition | Population:   - Adult population (>18 years) exclusively |
| Study Design:   - Publication describing findings from core outcome initiatives related to other pediatric conditions (non-specific to PKU and/or MCAD) | Study Design:   - Animal/In-vitro studies - Case reports and case series with less than 5 subjects |

***Level 2: Full-text screening***

**-**Two independent reviewers screened articles in duplicate.

1. Is the full-text available? (If full-text has not been uploaded, then skip for now).
   - Yes (Include) – proceed to Q2
   - No – not available (Exclude)
   - No – abstract/conference proceeding only (Exclude)
2. Is the article published in English?
   - Yes (Include) – proceed to Q3
   - No (Exclude)
3. Is the article focused on children (≤18 years) diagnosed with a pediatric condition?
   - Yes (Include) – proceed to Q4.
   - No* (Exclude)
4. Is the article a study design of interest?
   - Yes* (Include)
   - No** (Exclude)

*Select “Yes” if it is a publication describing findings from core outcome initiatives related to other pediatric conditions (non-specific to PKU or MCAD).

**Select “No” if any of the following applies: (i) animal/in-vivo/in-vitro studies; (ii) case reports and case series with less than 5 subjects.

**1.5: Data Extraction Fields**

| **Data Category** | **Data Fields** |
| --- | --- |
| **Study Characteristics** | 1. Authors, Year (RefID) 2. Journal 3. Country of publication 4. Study design |
| **Participant Characteristics** | 1. Age 2. Diagnosed disease 3. Disease severity (%) 4. % of patients identified by newborn screening |
| **Intervention Details**  (for studies whose primary purpose was to evaluate one or more interventions) | If an intervention was used in the study, then describe its specific characteristics. |
| **Comparator Details**  (for studies whose primary purpose was to evaluate one or more interventions**)** | If a comparator treatment was used in the study, then describe its specific characteristics. |
| **Outcome Details** | 1. Describe the outcome 2. Describe the scale/tool used to measure the outcome (e.g. laboratory value, clinician-reported, patient/care provider-reported) 3. Was the measurement scale/tool validated? (Include related citations, if provided) |
