# Supplementary material for: Outcomes in pediatric studies of medium-chain acyl-coA dehydrogenase (MCAD) deficiency and phenylketonuria (PKU): a review
Source: Orphanet J Rare Dis. 2020 Jan 14;15:12. doi: 10.1186/s13023-019-1276-1 (PMC6961328; doi:10.1186/s13023-019-1276-1)
Supplement: Supplementary file 2 — Additional file 2. Microsoft Word document (.docx). Title: List of eligible studies excluded from data synthesis. Description: A table listing the references for eligible PKU and COMET database articles that were excluded from data synthesis [file 13023_2019_1276_MOESM2_ESM.docx]

**Additional File 1**. List of eligible studies excluded from data synthesis (n=188).

| 1. Schular A, Somogyi C, Toros I, et al. A longitudinal study of phenylketonuria based on the data of the Budapest Screening Center. *Eur J Pediatr*. 1996;155(Suppl 1):S50-S52. doi:10.1007/pl00014249  2. Buist NRM, Prince AP, Huntington KL, Tuerck JM, Waggoner DD. A new amino acid mixture permits new approaches to the treatment of phenylketonuria. *Acta Pædiatrica*. 1994;407:75-77. doi:10.1111/j.1651-2227.1994.tb13458.x  3. Griffiths AM, Otley AR, Hyams J, et al. A review of activity indices and end points for clinical trials in children with Crohn’s disease. *Inflamm Bowel Dis*. 2005;11(2):185-196. doi:https://doi.org/10.1097/00054725-200502000-00013  4. Allori AC, Kelley T, Meara JG, et al. A standard set of outcome measures for the comprehensive appraisal of cleft care. *Cleft Palate-Craniofacial J*. 2017;54(5):540-554. doi:10.1597/15-292  5. Langenbeck U, Behbehani A, Mench-Hoinowski A. A synopsis of the unconjugated acidic transamination metabolites of phenylalanine in phenylketonuria. *J Inherit Metab Dis*. 1992;15(1):136-144. doi:10.1007/BF01800355  6. MacDonald A, Harris G, Rylance G, Asplin D, Booth IW. Abnormal feeding behaviours in phenylketonuria. *J Hum Nutr Diet*. 1997;10(3):163-170. doi:10.1046/j.1365-277X.1997.00050.x  7. Reddel HK, Taylor R, Bateman ED, et al. An official American Thoracic Society/European Respiratory Society Statement: Asthma control and exacerbations. *Am J Respir Crit Care Med*. 2009;180(1):59-99. doi:10.1164/rccm.200801-060st  8. Treacy EP, Delente JJ, Elkas G, et al. Analysis of phenylalanine hydroxylase genotypes and pyperphenylalaninemia phenotypes using L-[1-13C] phenylalanine oxidation rates in vivo: A pilot study. *Pediatr Res*. 1997;42(4):430-435. doi:10.1203/00006450-199710000-00002  9. Sierra C, Vilaseca MA, Moyano D, et al. Antioxidant status in hyperphenylalaninemia. *Clin Chim Acta*. 1998;276(1):1-9. doi:10.1016/S0009-8981(98)00091-6  10. Busse WW, Morgan WJ, Taggart V, Togias A. Asthma outcomes workshop: Overview. *J Allergy Clin Immunol*. 2012;129(3):S1-S8. doi:10.1016/j.jaci.2011.12.985  11. Scheibenreiter S, Tiefenthaler M, Hinteregger V, et al. Austrian report on longitudinal outcome in phenylketonuria. *Eur J Pediatr*. 1996;155(Suppl 1):S45-S49. doi:10.1007/pl00014248  12. Walter JH, Tyfield LA, Holton JB, Johnson C. Biochemical control, genetic analysis and magnetic resonance imaging in patients with phenylketonuria. *Eur J Pediatr*. 1993;152:822-827.  13. Leuzzi V, Trasimeni G, Gualdi GF, Antonozzi I. Biochemical, clinical and neuroradiological (MRI) correlations in late-detected PKU patients. *J Inherit Metab Dis*. 1995;18(5):624-634. doi:10.1007/BF02436009  14. Schulpis KH, Nyalala JO, Papakonstantinou ED, et al. Biotin recycling impairment in phenylketonuric children with seborrheic dermatitis. *Int J Dermatol*. 1998;37:918-921.  15. Schulpis KH, Papakonstantinou E, Kalogirou S. Biotinidase activity in patients with phenylketonuria. *J Inherit Metab Dis*. 1995;18(6):750-751. doi:10.1007/BF02436767  16. Allen J, Baur L, Waters D, et al. Body protein in prepubertal children with phenylketonuria. *Eur J Clin Nutr*. 1996;50(3):178-186.  17. Zeman J, Bayer M, Štěpán J. Bone mineral density in patients with phenylketonuria. *Acta Paediatr*. 1999;88(12):1348-1351. doi:10.1111/j.1651-2227.1999.tb01049.x  18. McMurry MP, Chan GM, Leonard CO, Ernst SL. Bone mineral status in children with phenylketonuria - Relationship to nutritional intake and phenylalanine. *Am J Clin Nutr*. 1992;55:997-1004.  19. Al-Qadreh A, Schulpis K, Athanasopoulou H, Mengreli C, Skarpalezou A, Voskaki I. Bone mineral status in children with phenylketonuria under treatment. *Acta Paediatr*. 1998;87(11):1162-1166. doi:10.1111/j.1651-2227.1998.tb00924.x  20. Fisch RO, Feinberg SB, Weisberg S, Day D. Bony changes of PKU neonates unrelated to phenylalanine levels. *J Inherit Metab Dis*. 1991;14(6):890-895. doi:10.1007/BF01800469  21. Toft PB, Lou HC, Krageloh-Mann I, et al. Brain magnetic resonance imaging in children with optimally controlled hyperphenylalaninaemia. *J Inherit Metab Dis*. 1994;17(5):575-583. doi:10.1007/bf00711594  22. Motzfeldt K, Lilje R, Nylander G. Breastfeeding in phenylketonuria. *Acta Paediatr*. 1999;432:25-27. doi:10.1111/j.1651-2227.1999.tb01151.x  23. Greve LC, Wheeler MD, Green-Burgeson DK, Zorn EM. Breast-feeding in the management of the newborn with phenylketonuria: A practical approach to dietary therapy. *J Am Diet Assoc*. 1994;94(3):305-309. doi:10.1016/0002-8223(94)90373-5  24. Schulman S, Fisch RO, Zempel CE, Gadish O, Chang P-N. Children with phenylketonuria: The interface of family and child functioning. *Dev Behav Pediatr*. 1991;12(5):315-321.  25. Mazzocco MMM, Yannicelli S, Nord AM, Van Doorninck W, Davidson-Mund AJ, Greene CL. Cognition and tyrosine supplementation among school-aged children with phenylketonuria. *Pediatr Forum*. 1992;146:1261-1264.  26. Krauch G, Müller E, Anninos A, Bremer HJ. Comparison of the protein quality of dietetically treated phenylketonuria patients with the recommendations of the WHO expert consultation. *Eur J Pediatr*. 1996;155(Suppl 1):S153-S157. doi:10.1007/pl00014235  27. Antoshechkin AG, Chentsova T V., Tatur VY, Naritsin DB, Railian GP. Content of phenylalanine, tyrosine and their metabolites in CSF in phenylketonuria. *J Inherit Metab Dis*. 1991;14(5):749-754. doi:10.1007/BF01799945  28. Vilaseca MA, Briones P, Ferrer I, et al. Controlled diet in phenylketonuria may cause serum carnitine deficiency. *J Inherit Metab Dis*. 1993;16(1):101-104. doi:10.1007/BF00711322  29. Awiszus D, Unger I. Coping with PKU: Results of narrative interview with parents. *Eur J Pediatr*. 1990;149(Suppl 1):S45-S51.  30. McGrath PJ, Walco GA, Turk DC, et al. Core outcome domains and measures for pediatric acute and chronic/recurrent pain clinical trials: PedIMMPACT recommendations. *J Pain*. 2008;9(9):771-783. doi:10.1016/j.jpain.2008.04.007  31. Hillman L, Schlotzhauer C, Lee D, et al. Decreased bone mineralization in children with phenylketonuria under treatment. *Eur J Pediatr*. 1996;155(Suppl 1):148-152. doi:10.1007/PL00014234  32. Artuch R, Vilaseca M-A, Moreno J, Lambruschini N, Cambra FJ, Campistol J. Decreased serum ubiquinone-10 concentrations in phenylketonuria. *Am J Clin Nutr*. 1999;70(5):892-895. doi:10.1093/ajcn/70.5.892  33. Schawhn B, Mokov E, Scheidhauer K, Lettgen B, Schonau E. Decreased trabecular bone mineral density in patients with phenylketonuria measured by peripheral quantitative computed tomography. *Acta Paediatr*. 1998;87(1):61-63. doi:10.1111/j.1651-2227.1998.tb01387.x  34. Weglage J, Pietsch M, Fünders B, Koch HG, Ullrich K. Deficits in selective and sustained attention processes in early treated children with phenylketonuria - Result of impaired frontal lobe functions? *Eur J Pediatr*. 1996;155(3):200-204. doi:10.1007/BF01953938  35. Doherty LB, Rohr FJ, Levy HL. Detection of phenylketonuria in the very early newborn blood specimen. *Pediatrics*. 1991;87(2):240-244.  36. Sinha IP, Gallagher R, Williamson PR, Smyth RL. Development of a core outcome set for clinical trials in childhood asthma: a survey of clinicians, parents, and young people. *Trials*. 2012;13:1-9. doi:10.1186/1745-6215-13-103  37. Allin BSR, Hall NJ, Ross AR, Marven SS, Kurinczuk JJ, Knight M. Development of a gastroschisis core outcome set. *Arch Dis Child Fetal Neonatal*. 2019;104:F76-F82. doi:10.1136/ archdischild-2017-314560  38. Seashore MR, Wappner R, Cho S, de la Cruz F. Development of guidelines for treatment of children with phenylketonuria: Report of a meeting at the National Institute of Child Health and Human Development held August 15, 1995, National Institutes of Health, Bethesda, Maryland. *Pediatrics*. 1999;104(e67):1-9. doi:10.1542/peds.104.6.e67  39. Murugupillai R, Ranganathan SS, Wanigasinghe J, Muniyandi R, Arambepola C. Development of outcome criteria to measure effectiveness of antiepileptic therapy in children. *Epilepsy Behav*. 2018;80:56-60. doi:10.1016/j.yebeh.2017.12.011  40. Endres W. Diet in phenylketonuria: How long? Policies under discussion. *Ann Nutr Metab*. 1998;42(2):63-67. doi:10.1159/000012719  41. Bick U, Fahrendorf AC, Vassallo P, Weglage J, Ullrich K. Disturbed myelination in patients with treated hyperphenylalaninaemia: Evaluation with magnetic resonance imaging. *Eur J Pediatr*. 1991;150:185-189.  42. MacDonald A, Rylance G, Asplin D, Hall S, Booth I. Does a single plasma phenylalanine predict quality of control in phenylketonuria? *Arch Dis Child*. 1998;78(2):122-126. doi:10.1136/adc.78.2.122  43. van Spronsen FJ, Verkerk PH, van Houten M, et al. Does impaired growth of PKU patients correlate with the strictness of dietary treatment? *Acta Paediatr*. 1997;86(8):816-818.  44. Riva E, Agostoni C, Biasucci G, et al. Early breastfeeding is linked to higher intelligence quotient scores in dietary treated phenylketonuric children. *Acta Paediatr*. 1996;85(1):56-58. doi:10.1111/j.1651-2227.1996.tb13890.x  45. Pietz J, Lutcke A, Sontheimer D, et al. EEG development in early treated PKU patients from birth to 6 years of age. *Eur J Pediatr*. 1990;149(Suppl 1):S28-S33. doi:10.1007/bf02126296  46. Smith I, Beasley MG, Ades AE. Effect on intelligence of relaxing the low phenylalanine diet in phenylketonuria. *Arch Dis Child*. 1990;65(3):311-316. doi:10.1136/adc.66.3.311  47. Jochum F, Terwolbeck K, Meinhold H, Behne D, Menzel H, Lombeck I. Effects of a low selenium state in patients with phenylketonuria. *Acta Paediatr*. 1997;86(7):775-777. doi:10.1111/j.1651-2227.1997.tb08587.x  48. Schmidt E, Burgard P, Rupp A. Effects of concurrent phenylalanine levels on sustained attention and calculation speed in patients treated early for phenylketonuria. *Eur J Pediatr*. 1996;155(Suppl 1):S82-S86. doi:10.1007/pl00014258  49. Calomme M, Vanderpas J, Francois B, et al. Effects of selenieum supplementation on thyroid hormone metabolism in phenylketonuria subjects on a phenylalanine restricted diet. *Biol Trace Elem Res*. 1995;47:349-353.  50. De Giorgis GF, Nonnis E, Crocioni F, et al. Evolution of daytime quiet sleep components in early treated phenylketonuric infants. *Brain Dev*. 1996;18:201-206.  51. Griffiths P, Tarrini M, Robinson P. Executive function and psychosocial adjustment in children with early treated phenylketonuria: Correlation with historical and concurrent phenylalanine levels. *J Intellect Disabil Res*. 1997;41(4):317-323. doi:10.1111/j.1365-2788.1997.tb00715.x  52. Griffiths P, Campbell R, Robinson P. Executive function in treated phenylketonuria as measured by the one-back and two-back versions of the continuous performance test. *J Inherit Metab Dis*. 1998;21(2):125-135. doi:10.1023/A:1005339524847  53. MacDonald A, Rylance G, Hall S, Asplin D, Booth I. Factors affecting the variation in plasma phenylalanine in patients with phenylketonuria on diet. *Arch Dis Child*. 1996;74(5):412-417. doi:10.1136/adc.74.5.412  54. Giovannini M, Agostoni C, Biasucci G, et al. Fatty acid metabolism in phenylketonuria. *Eur J Pediatr*. 1996;155(Suppl 1):132-135.  55. MacDonald A, Rylance G, Asplin D, Hall K, Harris G, Booth I. Feeding problems in young PKU children. *Acta Pædiatrica*. 1994;407(Suppl):73-74. doi:10.1111/j.1651-2227.1994.tb13457.x  56. Greeves LG, Carson DJ, Magee A, Patterson CC. Fractures and phenylketonuria. *Acta Paediatr*. 1997;86(3):242-244. doi:10.1111/j.1651-2227.1997.tb08882.x  57. Hashishe MM. Genetic study of phenylketonuria. *J Egypt Public Health Assoc*. 1992;67(3-4):443-463.  58. Schaefer F, Burgard P, Batzler U, et al. Growth and skeletal muaturation in children with phenylketonuria. *Acta Paediatr*. 1994;83(5):534-541. doi:10.1111/j.1651-2227.1994.tb13075.x  59. Schulpis KH, Platokouki H, Papakonstantinou ED, Adamtziki E, Bargeliotis A, Aronis S. Haemostatic variables in phenylketonuric children under dietary treatment. *J Inherit Metab Dis*. 1996;19(5):603-609. doi:10.1007/BF01799833  60. Lucas A, Baker BA, Morley RM. Hyperphenylalaninaemia and outcome in intravenously fed preterm neonates. *Arch Dis Child*. 1993;68:579-583. doi:10.1136/adc.68.5_Spec_No.579  61. Verkerk PH, Van Spronsen FJ, Smit GPA, Sengers RCA. Impaired prenatal and postnatal growth in Dutch patients with phenylketonuria. *Arch Dis Child*. 1994;71(2):114-118. doi:10.1136/adc.71.2.114  62. Diamond A, Herzberg C. Impaired sensitivity to visual contrast in children treated early and continuously for phenylketonuria. *Brain*. 1996;119(2):523-538. doi:10.1093/brain/119.2.523  63. Röricht S, Meyer BU, Irlbacher K, Ludolph AC. Impairment of callosal and corticospinal system function in adolescents with early-treated phenylketonuria: A transcranial magnetic stimulation study. *J Neurol*. 1999;246(1):21-30. doi:10.1007/s004150050301  64. Böhles H, Ullrich K, Endres W, Behbehani AW, Wendel U. Inadequate iron availability as a possible cause of low serum carnitine concentrations in patients with phenylketonuria. *Eur J Pediatr*. 1991;150(6):425-428. doi:10.1007/BF02093725  65. Hjelm M, Seakins J, Antoshechkin A. Indications of changed amino acid homeostasis in untreated and treated PKU. *Acta Pædiatrica*. 1994;407(Suppl):57-59. doi:10.1111/j.1651-2227.1994.tb13452.x  66. Stemerdink BA, van der Meere JJ, wan der Molen MW, et al. Information processing in patients with early and continuously-treated phenylketonuria. *Eur J Pediatr*. 1995;154:739-746. doi:10.1007/bf02276719  67. Zeman J, Pijackova A, Behulova J, Urge O, Saligova D, Hyanek J. Intellectual and school performance in adolescents with phenylketonuria according to their dietary compliance: The Czech-Slovak Collaborative Study. *Eur J Pediatr*. 1996;155(Suppl 1):S56-S58. doi:10.1007/pl00014251  68. Azen CG, Koch R, Friedman EG, et al. Intellectual development in 12-year-old children treated for phenylketonuria. *Am J Dis Child*. 1991;145(1):35-39. doi:10.1001/archpedi.1991.02160010037012  69. Burgard P, Schmidt E, Rupp A, Schneider W, Bremer HJ. Intellectual development of the patients of the German Collaborative Study of children treated for phenylketonuria. *Eur J Pediatr*. 1996;155(Suppl 1):S33-S38. doi:10.1007/pl00014245  70. Hilliges C, Awiszus D, Wendel U. Intellectual performance of children with maple syrup urine disease. *Eur J Pediatr*. 1993;152(2):144-147. doi:10.1007/BF02072492  71. Smith I, Beasley MG, Ades AE. Intelligence and quality of dietary treatment in phenylketonuria. *Arch Dis Child*. 1990;65(5):472-478. doi:10.1136/adc.65.5.472  72. Costello PM, Beasley MG, Tillotson SL, Smith I. Intelligence in mild atypical phenylketonuria. *Eur J Pediatr*. 1994;153(4):260-263. doi:10.1007/s004310050132  73. Dennis M, Lockyer L, Lazenby AL, Donnelly RE, Wilkinson M, Schoonheyt W. Intelligence patterns among children with functioning autism, phenylketonuria, and childhood head injury. *J Autism Dev Disord*. 1999;29(1):5-16.  74. Gourovitch ML, Craft S, Dowton SB, Ambrose P, Sparta S. Interhemispheric transfer in children with early-treated phenylketonuria. *J Clin Exp Neuropsychol*. 1994;16(3):393-404. doi:10.1080/01688639408402650  75. Miranda Da Cruz BDO, Seidler H, Widhalm K. Iron status and iron supplementation in children with classical phenylketonuria. *J Am Coll Nutr*. 1993;12(5):531-536. doi:10.1080/07315724.1993.10718348  76. Jochum F, Terwolbeck K, Meinhold H, Behne D, Menzel H, Lombeck I. Is there any health risk of low dietary selenium supply in pku-children? *Nutr Res*. 1999;19(3):349-360.  77. Craft S, Gourovitch ML, Dowton SB, Swanson JM, Bonforte S. Lateralized deficits in visual attention in males with developmental dopamine depletion. *Neuropsychologia*. 1992;30(4):341-351. doi:10.1016/0028-3932(92)90107-W  78. Oura T. Life-long treatment for phenylketonuria. *Southeast Asian Jounral Trop Med Public Heal*. 1999;30(Suppl 2):61-62.  79. Pöge AP, Bäumann K, Müller E, Leichsenring M, Schmidt H, Bremer HJ. Long-chain polyunsaturated fatty acids in plasma and erythrocyte membrane lipids of children with phenylketonuria after controlled linoleic acid intake. *J Inherit Metab Dis*. 1998;21(4):373-381. doi:10.1023/A:1005350523826  80. Cabalska MB, Nowaczewska I, Sendecka E, Zorska K. Longitudinal study on early diagnosis and treatment of phenylketonuria in Poland. *Eur J Pediatr*. 1996;155(Suppl 1):S53-S55. doi:10.1007/pl00014250  81. Koch R, Moseley K, Ning J, Romstad A, Guldberg P, Guttler F. Long-term beneficial effects of the phenylalanine-restricted diet in late-diagnosed individuals with phenylketonuria. *Mol Genet Metab*. 1999;67(2):148-155. doi:10.1006/mgme.1999.2863  82. Rey F, Abadie V, Plainguet F, Rey J. Long-term follow up of patients with classical phenylketonuria after diet relaxation at 5 years of age: The Paris Study. *Eur J Pediatr*. 1996;155(Suppl 1):S39-S44. doi:10.1007/pl00014246  83. Cechak P, Hejcmanova L, Rupp A. Long-term follow-up of patients treated for phenylketonuria (PKU): Results from the Prague PKU Center. *Eur J Pediatr*. 1996;155(Suppl 1):S59-63.  84. Bodley JL, Austin VJ, Hanley WB, Clarke JTR, Zlotkin S. Low iron stores in infants and children with treated phenylketonuria: A population at risk for iron-deficiency anaemia and associated cognitive deficits. *Eur J Pediatr*. 1993;152(2):140-143. doi:10.1007/BF02072491  85. Wappner R, Cho S, Kronmal RA, Schuett V, Seashore MR. Management of phenylketonuria for optimal outcome: A review of guidelines for phenylketonuria management and a report of surveys of parents, patients, and clinic directors. *Pediatrics*. 1999;104(e68):4-9. doi:10.1542/peds.104.6.e68  86. Morris C, Janssens A, Shiling V, et al. Meaningful health outcomes for paediatric neurodisability: Stakeholder prioritisation and appropriateness of patient reported outcome measures. *Health Qual Life Outcomes*. 2015;13(87):1-9. doi:10.1186/s12955-015-0284-7  87. Pavletic SZ, Martin P, Lee SJ, et al. Measuring therapeutic response in chronic graft-versus-host disease: National Institutes of Health consensus development project on criteria for clinical trials in chronic graft-versus-host disease: IV. Response criteria working group report. *Biol Blood Marrow Transplant*. 2006;12(3):252-266. doi:10.1016/j.bbmt.2006.01.008  88. Carlson GA, Jensen PS, Findling RL, et al. Methodological issues and controversies in clinical trials with child and adolescent patients with bipolar disorder: report of a consensus conference. *J Child Adolesc Psychopharmacol*. 2003;13(1):13-27. http://onlinelibrary.wiley.com/o/cochrane/clcmr/articles/CMR-10847/frame.html.  89. Zekanowski C, Nowacka M, Cabalska B, Bal J. Molecular basis of mild hyperphenylalaninaemia in Poland. *J Med Genet*. 1997;34:1035-1036.  90. Enns GM, Martinez DR, Kuzmin AI, et al. Molecular correlations in phenylketonuria: Mutation patterns and corresponding biochemical and clinical phenotypes in a heterogeneous california population. *Pediatr Res*. 1999;46(5):594-594. doi:10.1203/00006450-199911000-00017  91. Zekanowski C, Nowacka M, Gizewska M, Filipowicz J, Cabalska B, Bal J. Mutations in exon 3 of the PAH gene causing mild hyperphenylalaninemia. *Genet Test*. 1999;3(3):297-299. doi:10.1089/109065799316626  92. Thompson AJ, Smith I, Brenton D, et al. Neurological deterioration in young adults with phenylketonuria. *Lancet*. 1990;336:602-605.  93. Weglage J, Pietsch M, Fünders B, Koch H, Ullrich K. Neurological findings in early treated phenylketonuria. *Acta Pædiatrica*. 1995;84(4):411-415. doi:10.1111/j.1651-2227.1995.tb13661.x  94. Ludolph A, Ullrich K, Nedjat S, H M, U B. Neurological outcome in 22 treated adolescents with hyperphenylalaninemia. *Acta Neurol Scand*. 1992;85(4):243-248. doi:10.1111/j.1600-0404.1992.tb04039.x  95. Coskun T, Topcu M, Ustundag I, et al. Neurophysiological studies of patients with classical phenylketonuria evaluation of results of IQ scores EEG and evoked potentials. *Turk J Pediatr*. 1993;35(1):1-10.  96. Burgard P, Rey F, Rupp A, Abadie V, Rey J. Neuropsychologic functions of early treated patients with phenylketonuria, on and off diet: Results of a cross-national and cross- sectional study. *Pediatr Res*. 1997;41(3):368-374. doi:10.1203/00006450-199703000-00011  97. Griffiths P, Paterson L, Harvie A. Neuropsychological effects of subsequent exposure to phenylalanine in adolescents and young adults with early-treated phenylketonuria. *J Intellect Disabil Res*. 1995;5:365-372.  98. Griffiths P, Ward N, Harvie A, Cockburn F. Neuropsychological outcome of experimental manipulation of phenylalanine intake in treated phenylketonuria. *J Inherit Metab Dis*. 1998;21(1):29-38. doi:10.1023/A:1005307229813  99. Welsh M, Pennigton B, Ozonoff S, Rouse B, McCabe ERB. Neuropsychology of early-treated PKU: Specific executive function deficits. *Child Dev*. 1990;61(February 1987):1697-1713.  100. Leuzzi V, Gualdi GF, Fabbrizi F, Trasimeni G, Di Biasi C, Antonozzi I. Neuroradiological (MRI) abnormalities in phenylketonuric subjects: Clinical and biochemical correlations. *Neuropediatrics*. 1993;24:302-306. doi:10.1055/s-2008-1071561  101. Tiliotson SL, Costello PM, Smith I. No reduction in birth weight in phenylketonuria. *Eur J Pediatr*. 1995;154(10):847-849. doi:10.1007/BF01959795  102. Schulz B, Bremer HJ. Nutrient intake and food consumption of adolescents and young adults with phenylketonuria. *Acta Pædiatrica*. 1995;84(7):743-748. doi:10.1111/j.1651-2227.1995.tb13748.x  103. Acosta PB, Yannicelli S, Marriage B, et al. Nutrient intake and growth of infants with phenylketonuria undergoing therapy. *J Pediatr Gastroenterol Nutr*. 1998;27(3):287-291. doi:10.1097/00005176-199809000-00003  104. Gropper SS, Naglak MC, Nardella M, Plyler A, Rarback S, Yannicelli S. Nutrient intakes of adolescents with phenylketonuria and infants and children with maple syrup urine disease on semisynthetic diets. *J Am Coll Nutr*. 1993;12(2):108-114. doi:10.1080/07315724.1993.10718290  105. Giovannini M, Biasucci G, Luotti D, Fiori L, Riva E. Nutrition in children affected by inherited metabolic diseases. *Ann 1st Super Sanita*. 1995;31(4):489-502.  106. Acosta PB, Greene C, Yannicelli S, et al. Nutrition studies in treated infants with phenylketonuria. *Int Pediatr*. 1993;8(1):63-73.  107. Giovannini M, Biasucci G, Agostoni C, Luotti D, Rottoli A, Riva E. Nutritional approach to PKU diet. *Int Pediatr*. 1995;10(1):100-104.  108. Fisberg RM, da Silva-Fernandes ME, Schmidt BJ, Fisberg M. Nutritional evaluation of children with phenylketonuria. *São Paulo Med J*. 1999;117(5):185-191. doi:10.1590/S1516-31801999000500002  109. Ramsey BW, Boat TF. Outcome measures for clinical trials in cystic fibrosis: Summary of a Cystic Fibrosis Foundation Consensus Conference. *J Pediatr*. 1994;124:177-192.  110. Ruemmele FM, Hyams JS, Otley A, et al. Outcome measures for clinical trials in paediatric IBD: An evidence-based, expert-driven practical statement paper of the paediatric ECCO committee. *Gut*. 2015;64(3):438-446. doi:10.1136/gutjnl-2014-307008  111. Beasley MG, Costello PM, Smith I. Outcome of treatment in young adults with phenylketonuria detected by routine neonatal screening between 1964 and 1971. *Q J Med*. 1994;87(3):155-160. doi:10.1093/oxfordjournals.qjmed.a068910  112. Weglage J, Rupp A, Schmidt E. Personality characteristics in patients with phenylketonuria treated early. *Pediatr Res*. 1994;35(5):611-613.  113. Burgard P, Rupp A, Konecki DS, Trefz FK, Schmidt H, Lichter-Konecki U. Phenylalanine hydroxylase genotypes, predicted residual enzyme activity and phenotypic parameters of diagnosis and treatment of phenylketonuria. *Eur J Pediatr*. 1996;155(Suppl 1):S11-S15. doi:10.1007/pl00014222  114. Smith AM, McMurry MP, Chan GM, Leonard CO. Phenylketonuria affects the selenium status of children, adolescents, and young adults. *J Trace Elem Exp Med*. 1994;7:39-45.  115. Hendrikx MMT, van der Schot LWA, Slijper FME, Huisman J, Kalverboer AF. Phenylketonuria and some aspects of emotional development. *Eur J Pediatr*. 1994;153:832-835. doi:10.1007/s004310050226  116. Fisch R, Matalon R, Weisberg S, Michals K. Phenylketonuria: Current dietary treatment practices in the United States and Canada. *J Am Coll Nutr*. 1997;16(2):147-151. doi:10.1080/07315724.1997.10718665  117. Cerone R, Schiaffino MC, Di Stefano S, Veneselli E. Phenylketonuria: Diet for life or not? *Acta Paediatr*. 1999;88(6):664-666. doi:10.1080/08035259950169350  118. Pietz J, Kreis R, Schmidt H, Meyding-Lamade UK, Rupp A, Boesch C. Phenylketonuria: Findings at MR imaging and localized in vivo H-1 MR spectrospocy of the brain in patients with early treatment. *Radiology*. 1996;201:413-420.  119. Pearsen KD, Gean-Marton AD, Levy HL, Davis KR. Phenylketonuria: MR imagining of the brain with clinical correlation. *Radiology*. 1990;177:437-440.  120. Van Spronsen FJ, van Dijk T, Smit GPA, et al. Phenylketonuria: plasma phenylalanine responses to different distributions of the daily phenylalanine allowance over the day. *Pediatrics*. 1996;97(6):839-844.  121. Van Spronsen FJ, Reijngoud DJ, Smit GPA, et al. Phenylketonuria: The in vivo hydroxylation rate of phenylalanine into tyrosine is decreased. *J Clin Invest*. 1998;101(12):2875-2880. doi:10.1172/JCI737  122. Fisch RO, Chang PN, Weisberg S, Guldberg P, Güttler F, Tsai MY. Phenylketonuric patients decades after diet. *J Inherit Metab Dis*. 1995;18(3):347-353. doi:10.1007/BF00710427  123. Dhondt JL, Largillière C, Moreno L, Farriaux JP. Physical growth in patients with phenylketonuria. *J Inherit Metab Dis*. 1995;18(2):135-137. doi:10.1007/BF00711748  124. Mcburnie MA, Kronmal RA, Schuett VE, Koch R, Azeng CG. Physical growth of children treated for phenylketonuria. *Ann Hum Biol*. 1991;18(4):357-368. doi:10.1080/03014469100001662  125. Gerdes A-M, Nielsen JB, Lou H, Guttler F. Plasma amino acids in term neonates and infants with phenylketonuria before and after institution of the diet. *Acta Paediatr Scand*. 1990;79(1):64-68. doi:10.1111/j.1651-2227.1990.tb11332.x  126. Agostoni C, Marangoni F, Riva E, Giovannini M, Galli C. Plasma arachidonic acid and serum thromboxane B2 concentrations in phenylketonuric children negatively correlate with dietary compliance. *Prostaglandins Leukot Essent Fat Acids*. 1997;56(3):219-222. doi:10.1016/S0952-3278(97)90538-X  127. Gropper SS, Yannicelli S. Plasma molybdenum concentrations in children with and without phenylketonuria. *Biol Trace Elem Res*. 1993;38(3):227-231. doi:10.1007/bf02785307  128. van Spronsen FJ, van Rijn M, van Dijk T, et al. Plasma phenylalanine and tyrosine responses to different nutritional conditions (fasting/postprandial) in patients with phenylketonuria: effect of sample timing. *Pediatrics*. 1993;92(4):570-573. http://www.ncbi.nlm.nih.gov/pubmed/8414829.  129. Fisberg RM, Eugênia M, Silva-Femandes DA, Fisberg M, Schmidt BJ. Plasma zinc, copper, and erythrocyte superoxide dismutase in children with phenylketonuria. *Nutrition*. 1999;15(6):449-452. doi:10.1016/S0899-9007(99)00082-9  130. Sanjurjo P, Perteagudo L, Soriano JR, Vilaseca A, Campistol J. Polyunsaturated fatty acid status in patients with phenylketonuria. *J Inherit Metab Dis*. 1994;17(6):704-709. doi:10.1007/bf00712012  131. Spronsen FJ, Smit GPA, Houten M, Sengers RCA, Verkerk PH. Predictors of mean phenylalanine levels during the first five years of life in patients with phenylketonuria who were treated early. *Acta Paediatr*. 1994;407(Suppl):70-72. doi:10.1111/j.1651-2227.1994.tb13456.x  132. Diamond A, Prevor MB, Callender G, Druin DP. Prefrontal cortex cognitive deficits in children treated early and continously for pku. *Monogr thesociety Res child Dev*. 1997;252(62:4).  133. Ruperto N, Ravelli A, Murray KJ, et al. Preliminary core sets of measures for disease activity and damage assessment in juvenile systemic lupus erythematosus and juvenile dermatomyositis. *Rheumatology*. 2003;42(12):1452-1459. doi:10.1093/rheumatology/keg403  134. de Sonneville LMJ, Schmidt E, Michel U, Batzler U. Preliminary neuropsychological test results. *Eur J Pediatr*. 1990;149(Suppl 1):S39-S44.  135. Van Der Stap DKD, Rider LG, Alexanderson H, et al. Proposal for a candidate core set of fitness and strength tests for patients with childhood or adult idiopathic inflammatory myopathies. *J Rheumatol*. 2016;43(1):169-176. doi:10.3899/jrheum.150270  136. Miller FW, Rider LG, Chung Y-L, et al. Proposed preliminary core set measures for disease outcome assessment in adult and juvenile idiopathic inflammatory myopathies. *Rheumatology*. 2001;40(11):1262-1273. doi:10.1093/rheumatology/40.11.1262  137. Acosta PB, Yannicelli S. Protein intake affects phenylalanine requirements and growth of infants with phenylketonuria. *Acta Pædiatrica*. 1994;407(Suppl):66-67. doi:10.1111/j.1651-2227.1994.tb13454.x  138. Acosta PB, Yannicelli S, Marriage B, et al. Protein Status of Infants with Phenylketonuria Undergoing Nutrition Management. *J Am Coll Nutr*. 1999;18(2):102-107. doi:10.1080/07315724.1999.10718834  139. Weglage J, Fünders B, Wilken B, et al. Psychological and social findings in adolescents with phenylketonuria. *Eur J Pediatr*. 1992;151(7):522-525. doi:10.1007/BF01957759  140. Burgard P, Armbruster M, Schmidt E, Rupp A. Psychopathology of patients treated early for phenylketonuria: results of the German collaborative study of phenylketonuria. *Acta Pædiatrica*. 1994;407:108-110. doi:10.1111/j.1651-2227.1994.tb13467.x  141. Weglage J, Funders B, Ullrich K, Rupp A, Schmidt E. Psychosocial aspects in phenylketonuria. *Eur J Pediatr*. 1996;155(Suppl 1):S101-S104. doi:10.1007/pl00014225  142. Greeves LG, Thomas PS, Carson DJ. Radiological assessment of the hand and wrist in phenylketonuria and hyperphenylalaninaemia. *Pediatr Radiol*. 1995;25(5):353-355. doi:10.1007/BF02021701  143. Burgard P, Bremer HJ, Bührdel P, et al. Rationale for the German recommendations for phenylalanine level control in phenylketonuria 1997. *Eur J Pediatr*. 1999;158(1):46-54. doi:10.1007/s004310051008  144. Cockburn F, Clark BJ. Recommendations for protein and amino acid intake in phenylketonuria patients. *Eur J Pediatr*. 1996;155(Suppl 1):S125-S129. doi:10.1007/pl00014229  145. Przyrembel H. Recommendations for protein and amino acid intake in phenylketonuria patients. *Eur J Pediatr*. 1996;155(Suppl 1):S130-S131. doi:10.1007/pl00014229  146. Acosta PB. Recommendations for protein and energy intakes by patients with phenylketonuria. *Eur J Pediatr*. 1996;155(Suppl 1):S121-S124. doi:10.1007/pl00014227  147. Smith I on behalf of the MRCWP on P. Recommendations on the dietary management of phenylketonuria. *Arch Dis Child*. 1993;68(3):426-427.  148. Galli C, Agostoni C, Mosconi C, Riva E, Carlo Salart P, Giovannini M. Reduced plasma C-20 and C-22 polyunsaturated fatty acids in children with phenylketonuria during dietary intervention. *J Pediatr*. 1991;119(4):562-567. doi:10.1016/S0022-3476(05)82405-9  149. Weglage J, Pietsch M, Denecke J, et al. Regression of neuropsychological deficits in early-treated phenylketonurics during adolescence. *J Inherit Metab Dis*. 1999;22(6):693-705. doi:10.1023/A:1005587915468  150. Lichter-Konecki U, Rupp A, Konecki DS, Trefz FK, Schmidt H, Burgard P. Relation between phenylalanine hydroxylase genotypes and phenotypic parameters of diagnosis and treatment of hyperphenylalaninaemic disorders. *J Inherit Metab Dis*. 1994;17(3):362-365. doi:10.1007/bf00711831  151. Allen JR, McCauley JC, Waters DL, O’Connor J, Roberts DC, Gaskin KJ. Resting energy expenditure in children with phenylketonuria. *Am J Clin Nutr*. 1995;62:797-801.  152. Michel U, Schmidt E, Batzler U. Results of psychological testing of patients aged 3-6 years. *Eur J Pediatr*. 1990;149(Suppl 1):S34-S38.  153. Smith I, Cook B, Beasley M. Review of neonatal screening programme for phenylketonuria. *BMJ*. 1991;303:333-335. doi:10.1136/bmj.303.6806.824  154. Lipinska L, Laskowska-Klita T, Cabalska B. Riboflavin status in phenylketonuric patients in the course of dietary treatment. *J Inherit Metab Dis*. 1994;17(2):242. doi:10.1007/BF00711625  155. Weglage J, Funders B, Wilken B, Schubert D, Ullrich K. School performance and intellectual outcome in adolescents with phenylketonuria. *Acta Paediatr*. 1993;81(6-7):582-586. doi:10.1111/j.1651-2227.1993.tb12759.x  156. Lombeck I, Jochum F, Terwolbeck K. Selenium status in infants and children with phenylketonuria and in maternal phenylketonuria. *Eur J Pediatr*. 1996;155(Suppl 1):S140-S144. doi:10.1007/pl00014232  157. Wilke BC, Vidailhet M, Favier A, et al. Selenium, glutathione peroxidase (GSH-Px) and lipid peroxidation products before and after selenium supplementation. *Clin Chim Acta*. 1992;207(1-2):137-142. doi:10.1016/0009-8981(92)90157-L  158. Schulpis KH, Nounopoulos C, Scarpalezou A, Bouloukos A, Missiou-Tsagarakis S. Serum carnitine level in phenylketonuric children under dietary control in Greece. *Acta Paediatr*. 1990;79(10):930-934. doi:10.1111/j.1651-2227.1990.tb11354.x  159. DeClue TJ, Davis J, Schocken DM, Kangas R, Benford SA. Serum lipid concentrations in subjects with phenylketonuria and their families. *Am J Dis Child*. 1991;145(11):1266-1268. doi:10.1001/archpedi.1991.02160110058020  160. Trefz FK, Batzler U, König T, et al. Significance of the in vivo deuterated phenylalanine load for long-term phenylalanine tolerance and psychointellectual outcome in patients with PKU. *Eur J Pediatr*. 1990;149(Suppl 1):S25-S27. doi:10.1007/BF02126295  161. Wendel U, Ullrich K, Schmidt H, Batzler U. Six-year follow up of phenylalanine intakes and plasma phenylalanine concentrations. *Eur J Pediatr*. 1990;149(Suppl 1):S13-S16.  162. Kalverboer A, van der Schot L, Hendrikx M, Huisman J, Slijper F, Stemerdink B. Social behaviour and task orientation in early-treated PKU. *Acta Paediatr*. 1994;83(Suppl 407):104-105. doi:10.1111/j.1651-2227.1994.tb13465.x  163. Ozanne AE, Krimmer H, Murdoch BE. Speech and language skills in children with early treated phenylketonuria. *Am J Ment*. 1990;94(6):625-632.  164. Lutz P, Schmidt H, Batzler U. Study design and description of patients. *Eur J Pediatr*. 1990;149(Suppl 1):S5-S12.  165. Leuzzi V, Rinalduzzi S, Chiarotti F, Garzia P, Trasimeni G, Accornero N. Subclinical visual impairment in phenylketonuria. A neurophysiological study (VEP-P) with clinical, biochemical, and neuroradiological (MRI) correlations. *J Inherit Metab Dis*. 1998;21(4):351-364. doi:10.1023/A:1005346422918  166. Azen C, Koch R, Friedman E, Wenz E, Fishler K. Summary of findings from the United States Collaborative Study of children treated for phenylketonuria. *Eur J Pediatr*. 1996;155(Suppl 1):S29-S32. doi:10.1007/bf03036507  167. Ponzone A, Guardamagna O, Ferraris S, Ferrero GB, Dianzani I, Cotton, Richard GH. Tetrahydrobiopterin loading test in hyperphenylalaninemia. *Pediatr Res*. 1991;30(5):435-438. doi:10.1203/00006450-199111000-00008  168. Kure S, Hou D-C, Ohura T, et al. Tetrahydrobiopterin-responsive phenylalanine hydroxylase deficiency. *J Pediatr*. 1999;135(3):375-378. doi:10.1016/S0022-3476(99)70138-1  169. Smith MA, Leeder SR, Jalaludin B, Smith WT. The asthma health outcome indicators study. *Aust N Z J Public Health*. 1996;20(1):69-75. doi:10.1111/j.1467-842X.1996.tb01340.x  170. Cardona F, Leuzzi V, Antonozzi I, Benedetti P, Loizzo A. The development of auditory and visual evoked potentials in early treated phenylketonuric children. *Electroencephalogr Clin Neurophysiol*. 1991;80(1):8-15. doi:10.1016/0168-5597(91)90036-W  171. Agostoni C, Riva E, Biasucci G, et al. The effects of n-3 and n-6 polyunsaturated fatty acids on plasma lipids and fatty acids of treated phenylketonuric children. *Prostaglandins, Leukot Essent Fat Acids*. 1995;53(6):401-404. doi:10.1016/0952-3278(95)90103-5  172. Kilpatrick NM, Awang H, Wilcken B, Christodoulou J. The implication of phenylketonuria on oral health. *Pediatr Dent*. 1999;21(7):433-437. http://pesquisa.bvsalud.org/portal/resource/pt/mdl-10633517.  173. Benit P, Rey F, Blandin-Savoja F, Munnich A, Abadie V, Rey J. The mutant genotype is the main determinant of the metabolic phenotype in phenylalanine hydroxylase deficiency. *Mol Genet Metab*. 1999;68(1):43-47. doi:10.1006/mgme.1999.2886  174. van der Schot LW, Doesburg WH, Sengers RCA. The phenylalanine response curve in relation to growth and mental development in the first years of life. *Acta Pædiatrica*. 1994;407(Suppl):68-69. doi:10.1111/j.1651-2227.1994.tb13455.x  175. Koch R, Fishler K, Azen C, Guldberg P, Guttler F. The Relationship of genotype to phenotype in phenylalanine hydroxylase deficiency. *Biochem Mol Med*. 1997;60(2):92-101. doi:10.1006/bmme.1997.2577  176. Calomme MR, Vanderpas JB, Francois B, et al. Thyroid function parameters during a selenium repletion/depletion study in phenylketonuric subjects. *Experientia*. 1995;51(12):1208-1215. doi:10.1007/bf01944738  177. Buist NRM, Prince AP, Huntington KL, Waggoner DD, Tuerck JM. Towards improving the diet for hyperphenylalaninemia and other metabolic disorders. *Int Pediatr*. 1993;8(1):80-88.  178. Reilly C, Barrett JE, Patterson CM, Tinggi U, Latham SL, Marrinan A. Trace element nutrition status and dietary intake of children with phenylketonuria. *Am J Clin Nutr*. 1990;52:159-165.  179. Griffiths P, Smith C, Harvie A. Transitory hyperphenylalaninaemia in children with continuously treated phenylketonuria. *Am J Ment Retard*. 1997;102(1):27-36. doi:10.1352/0895-8017(1997)102<0027:thicwc>2.0.co;2  180. Buist NRM, Waggoner DD, Huntington KL, Tuerck JM. Treatment of hyperphenylalaninemia More needs to be done. *Int Pediatr*. 1995;10(1):105-108.  181. Smith I. Treatment of phenylalanine hydroxylase deficiency. *Acta Pædiatrica*. 1994;407(Suppl):60-65. doi:10.1111/j.1651-2227.1994.tb13453.x  182. Prince AP, McMurry MP, Buist NRM. Treatment products and approaches for phenylketonuria: Improved palatability and flexibility demonstrate safety, efficacy and acceptance in US clinical trials. *J Inherit Metab Dis*. 1997;20(4):486-498. doi:10.1023/A:1005337126669  183. Legido A, Tonyes L, Carter D, Schoemaker A, Di George A, Grover WD. Treatment variables and intellectual outcome in children with classic phenylketonuria: A single-center-based study. *Clin Pediatr (Phila)*. 1993;32(7):417-425. doi:10.1177/000992289303200706  184. Berry HK, Brunner RL, Hunt MM, White PP. Valine, Isoleucine, and Leucine: A New Treatment for Phenylketonuria. *Am J Dis Child*. 1990;144:539-543.  185. Leuzzi V, Cardona F, Antonozzi I, Loizzo A. Visual auditory and somatosensorial evoked potentials in early and late treated adolescents with phenylketonuria. *J Clin Neurophysiol*. 1994;11(6):602-606.  186. Hanley WB, Feigenbaum ASJ, Schoonheyt WE, Austin VJ. Vitamin B12 deficiency in adolescents and young adults with phenylketonuria. *Eur J Pediatr*. 1996;155(Suppl 1):S145-S147. doi:10.1016/0140-6736(93)92047-W  187. Prince AP, Leklem JE. Vitamin B-6 status of alcohol-aged patients with phenylketonuria. *Am J Clin*. 1994;60:262-268.  188. Ullrich K, Moller H, Weglage J, et al. White matter abnormalities in phenylketonuria: Results of magnetic resonance measurements. *Acta Paediatr*. 1994;407(Suppl):78-82. doi:10.1111/j.1651-2227.1994.tb13459.x |
| --- |
