# Supplementary material for: Outcomes in pediatric studies of medium-chain acyl-coA dehydrogenase (MCAD) deficiency and phenylketonuria (PKU): a review
Source: Orphanet J Rare Dis. 2020 Jan 14;15:12. doi: 10.1186/s13023-019-1276-1 (PMC6961328; doi:10.1186/s13023-019-1276-1)
Supplement: Supplementary file 4 — Additional file 4. Microsoft Word document (.docx). Title: Measurement instruments for typically self-reported or neuropsychological MCAD deficiency outcomes. Description: Includes frequency data and references for listed measurement instruments [file 13023_2019_1276_MOESM4_ESM.docx]

**Additional File 4.** Measurement instruments for typically self-reported or neuropsychological MCAD deficiency outcomes.

| **Outcome** | **Measurement Instrument** | **# (%) of articles** | **References** |
| --- | --- | --- | --- |
| **CORE AREA: GROWTH AND DEVELOPMENT** | | | |
| **Domain: Cognition and Development** | | | |
| **Cognition and intelligence/IQ (n=14)** | Bayley Scales of Infant and Toddler Development [1] | 2 (14%) | [2,3] |
|  | Brunet-Lezine scale [4] | 2 (14%) | [5,6] |
|  | Denver Developmental Screening Test [7] | 1 (7%) | [2] |
|  | Kaufman Assessment Battery for Children [8] | 1 (7%) | [2] |
|  | McCarthy Scales of Children’s Abilities [9] | 2 (14%) | [5,6] |
|  | Snijders-Oomen Nonverbal Intelligence Test [10] | 1 (7%) | [2] |
|  | The Woodcock-Johnson Tests of Cognitive Abilities and Achievement [11] | 1 (7%) | [12] |
|  | Wechsler (age-appropriate version) [13] | 3 (21%) | [2,5,6] |
|  | Unclear | 10 (71%) | [2,14–22] |
| **Overall child development (n=14)** | Vineland Adaptive Behavior Scale [23] | 2 (14%) | [3,12] |
|  | Unclear | 12 (86%) | [14,15,29,30,18,19,21,24–28] |
| **Sensorimotor and motor functioning (n=7)** | Bayley Scales of Infant and Toddler Development [1] | 1 (14%) | [3] |
|  | Brunet-Lezine scale [4] | 2 (29%) | [5,6] |
|  | McCarthy Scales of Children’s Abilities [9] | 2 (29%) | [5,6] |
|  | Wechsler (age-appropriate version) [13] | 2 (29%) | [5,6] |
|  | Unclear | 4 (57%) | [14,21,22,31] |
| **CORE AREA: LIFE IMPACT** | | | |
| **Domain: Child and Caregiver/Family Life Impact** | | | |
| **Child quality of life (n=1)** | Unclear | 1 (100%) | [32] |
| **Caregiver/family psychosocial well-being (n=2)** | Parenting Stress Index [33] | 2 (100%) | [3,12] |
| **Parental experiences with illness care and prevention (n=1)** | Unclear | 1 (100%) | [34] |
| **Domain: Child Behaviour, Mental Health, and Temperament** | | | |
| **Behaviour problems and externalizing mental health or behaviour disorders (n=4)** | Behaviour Assessment System for Children [35] | 1 (25%) | [14] |
|  | Unclear | 3 (75%) | [17,18,28] |
| **Attention-deficit hyperactivity disorder (ADHD) or ADHD-like symptoms (n=2)** | Unclear | 2 (100%) | [14,18] |
| **Internalizing mental health or mood disorders and associated symptoms (n=1)** | Behaviour Assessment System for Children [35] | 1 (100%) | [14] |
|  | Unclear | 1 (100%) | [14] |
| **Autism spectrum disorder (ASD) or ASD-like symptoms (n=1)** | Unclear | 1 (100%) | [36] |
| **Tic disorder (n=1)** | Unclear | 1 (100%) | [14] |

**References**

1. Bayley N. Bayley Scales of Infant and Toddler Development. 3rd ed. San Antonio, TX: Harcourt Assessment; 2006.

2. Gramer G, Haege G, Fang-Hoffmann J, Hoffmann GF, Bartram CR, Hinderhofer K, et al. Medium-chain acyl-coA dehydrogenase deficiency: Evaluation of genotype-phenotype correlation in patients detected by newborn screening. JIMD Rep. 2015;May:101–12.

3. Waisbren SE, Albers S, Amato S, Ampola M, Brewster TG, Demmer L, et al. Effect of expanded newborn screening for biochemical genetic disorders on child outcomes and parental stress. J Am Med Assoc [Internet]. 2003;290:2564–72. Available from: http://ovidsp.ovid.com/ovidweb.cgi?T=JS&PAGE=reference&D=emed8&NEWS=N&AN=37443286

4. Brunet O, Lezine I. Desenvolvimento psicológico da primeira infância. Porto Alegre: Artes Médicas; 1981.

5. Couce ML, Castineiras DE, Moure JD, Cocho JA, Sanches-Pintos P, Garcia-Villoria J, et al. Relevance of expanded neonatal screening of medium-chain acyl co-A dehygrogenase deficiency: Outcome of a decade in Galicia (Spain). JIMD Rep. 2011;June:131–6.

6. Couce ML, Castineiras DE, Bóveda MD, Baña A, Cocho JA, Iglesias AJ, et al. Evaluation and long-term follow-up of infants with inborn errors of metabolism identified in an expanded screening programme. Mol Genet Metab [Internet]. Elsevier Inc.; 2011;104:470–5. Available from: http://dx.doi.org/10.1016/j.ymgme.2011.09.021

7. Frankenburg W, Dodds J, Archer P. Denver II Technical Manual. Denver, CO: Denver Developmental Materials; 1990.

8. Kaufman AS, Kaufman NL. Kaufman Assessment Battery for Children [Internet]. 2nd ed. Bloomington, MN: Pearson; 2018. Available from: https://www.pearsonassessments.com/store/usassessments/en/Store/Professional-Assessments/Cognition-%26-Neuro/Gifted-%26-Talented/Kaufman-Assessment-Battery-for-Children-%7C-Second-Edition-Normative-Update/p/100000088.html

9. McCarthy D. McCarthy Scales of Children’s Abilities. New York, NY: The Psychological Corporation; 1972.

10. Tellegen PJ, Laros JA. The construction and validation of a nonverbal test of intelligence: The revision of the Snijders-Oomen tests. Eur J Psychol Assess. 1993;9:147–57.

11. Schrank FA, McGrew KS, Mather N, Woodcock R. Woodcock-Johnson IV. Rolling Meadows, IL: Riverside Publishing; 2014.

12. Joy P, Black C, Rocca A, Haas M, Wilcken B. Neuropsychological functioning in children with medium chain acyl coenzyme A dehydrogenase deficiency (MCADD): The impact of early diagnosis and screening on outcome. Child Neuropsychol. 2009;15:8–20.

13. Wechsler D. Wechsler Intelligence Scale for Children. 5th ed. Bloomington, MN: Pearson; 2014.

14. Waisbren SE, Landau Y, Wilson J, Vockley J. Neuropsychological outcomes in fatty acid oxidation disorders: 85 cases detected by newborn screening. Dev Disabil Res Rev. 2013;17:260–8.

15. Wilcken B, Haas M, Joy P, Wiley V, Bowling F, Carpenter K, et al. Expanded newborn screening: Outcome in screened and unscreened patients at age 6 years. Pediatrics [Internet]. 2009;124:e241–8. Available from: http://pediatrics.aappublications.org/cgi/doi/10.1542/peds.2008-0586

16. Hsu H-W, Zytkovicz TH, Comeau AM, Strauss AW, Marsden D, Shih VE, et al. Spectrum of medium-chain acyl-coA dehydrogenase deficiency detected by newborn screening. Pediatrics [Internet]. 2008;121:e1108–14. Available from: http://pediatrics.aappublications.org/cgi/doi/10.1542/peds.2007-1993

17. Wilson CJ, Champion MP, Collins JE, Clayton PT, Leonard J V. Outcome of medium chain acyl-CoA dehydrogenase deficiency after diagnosis. Arch Dis Child. 1999;80:459–62.

18. Iafolla AK, Thompson RJ, Roe CR. Medium-chain acyl-coenzyme A dehydrogenase deficiency: Clinical course in 120 affected children. J Pediatr. 1994;March:409–15.

19. Hinton CF, Homer CJ, Thompson AA, Williams A, Hassell KL, Feuchtbaum L, et al. A framework for assessing outcomes from newborn screening: on the road to measuring its promise. Mol Genet Metab. 2016;118:221–9.

20. Wright EL, Van Hove JLK, Thomas J. Mountain States Genetics Regional Collaborative Center’s metabolic newborn screening long-term follow-up study: A collaborative multi-site approach to newborn screening outcomes research. Genet Med. 2010;12:S228–41.

21. Feuchtbaum L, Dowray S, Lorey F. The context and approach for the California newborn screening short- and long-term follow-up data system: Preliminary findings. Genet Med. 2010;12:242–50.

22. Lindner M, Gramer G, Haege G, Fang-Hoffmann J, Schwab KO, Tacke U, et al. Efficacy and outcome of expanded newborn screening for metabolic diseases - Report of 10 years from South-West Germany. Orphanet J Rare Dis. 2011;6:1–10.

23. Sparrow SS, Cicchetti D V, Saulnier CA. Vineland Adaptive Behavior Scales. 3rd ed. Bloomington, MN: Pearson; 2016.

24. Andresen BS, Bross P, Udvari S, Kirk J, Gray G, Kmoch S, et al. The molecular basis of medium-chain acyl-CoA dehydrogenase (MCAD) deficiency in compound heterozygous patients: Is there correlation between genotype and phenotype? Hum Mol Genet. 1997;6:695–707.

25. Pollitt RJ, Leonard JV. Prospective surveillance study of medium chain acyl-CoA dehydrogenase deficiency in the UK. Arch Dis Child. 1998;79:116–9.

26. Klose DA, Kolker S, Heinrich B, Prietsch V, Mayatepek E, von Kries R, et al. Incidence and short-term outcome of children with symptomatic presentation of organic acid and fatty acid oxidation disorders in Germany. Pediatrics. 2002;110:1204–11.

27. Liebl B, Nennstiel-Ratzel U, Roscher A, von Kries R. Data required for the evaluation of newborn screening programmes. Eur J Pediatr. 2003;162:S57–61.

28. Berry SA, Jurek AM, Anderson C, Bentler K. The inborn errors of metabolism information system: A project of the Region 4 Genetics Collaborative Priority 2 Workgroup. Genet Med. 2010;12:S215–9.

29. Purevsuren J, Hasegawa Y, Fukuda S, Kobayashi H, Mushimoto Y, Yamada K, et al. Clinical and molecular aspects of Japanese children with medium chain acyl-CoA dehydrogenase deficiency. Mol Genet Metab [Internet]. Elsevier Inc.; 2012;107:237–40. Available from: http://dx.doi.org/10.1016/j.ymgme.2012.06.010

30. Huang X, Yang L, Tong F, Yang R, Zhao Z. Screening for inborn errors of metabolism in high-risk children: A 3-year pilot study in Zhejiang Province, China. BMC Pediatr [Internet]. BioMed Central Ltd; 2012;12:1–7. Available from: http://www.biomedcentral.com/1471-2431/12/18

31. Horvath GA, Davidson AGF, Stockler-Ipsiroglu SG, Lillquist YP, Waters PJ, Olpin S, et al. Newborn screening for MCAD. Can J Public Heal. 2003;99:276–80.

32. Hatam N, Shirvani S, Javanbakht M, Askarian M, Rastegar M. Cost-utility analysis of neonatal screening program, Shiraz University of Medical Sciences, Shiraz, Iran, 2010. Iran J Pediatr. 2013;23:493–500.

33. Abidin RR. Parenting Stress Index. 4th ed. Lutz, FL: PAR;

34. Piercy H, Machaczek K, Ali P, Yap S. Parental experiences of raising a child with medium chain acyl-coA dehydrogenase deficiency. Glob Qual Nurs Res [Internet]. 2017;4. Available from: http://journals.sagepub.com/doi/10.1177/2333393617707080

35. Reynolds CR, Kamphaus RW. Behaviour Assessment System for Children [Internet]. 3rd ed. Bloomington, MN: Pearson; 2015. Available from: https://www.pearsonassessments.com/store/usassessments/en/Store/Professional-Assessments/Behavior/Comprehensive/Behavior-Assessment-System-for-Children-%7C-Third-Edition-/p/100001402.html

36. Tal G, Pitt J, Morrisy S, Tzanakos N, Boneh A. An audit of newborn screening procedure: Impact on infants presenting clinically before results are available. Mol Genet Metab [Internet]. Elsevier Inc.; 2015;114:403–8. Available from: http://dx.doi.org/10.1016/j.ymgme.2014.12.435
