# Supplementary material for: Outcomes in pediatric studies of medium-chain acyl-coA dehydrogenase (MCAD) deficiency and phenylketonuria (PKU): a review
Source: Orphanet J Rare Dis. 2020 Jan 14;15:12. doi: 10.1186/s13023-019-1276-1 (PMC6961328; doi:10.1186/s13023-019-1276-1)
Supplement: Supplementary file 5 — Additional file 5. Microsoft Word document (.docx). Title: Measurement instruments for typically self-reported or neuropsychological PKU outcomes. Description: Includes frequency data and references for listed measurement instruments [file 13023_2019_1276_MOESM5_ESM.docx]

**Additional File 5.** Measurement instruments for typically self-reported or neuropsychological PKU outcomes.

| **Outcome** | **Measurement Instrument** | **# (%) of articles** | **References** |
| --- | --- | --- | --- |
| **CORE AREA: GROWTH AND DEVELOPMENT** | | | |
| **Domain: Cognition and Development** | | | |
| **Cognition and intelligence/IQ (n=82)** | Ages and Stages Questionnaires [1] | 1 (1%) | [2] |
|  | Amsterdam Neuropsychological Tasks [3] | 3 (4%) | [4–6] |
|  | Basic Steps of Development [7] | 1 (1%) | [8] |
|  | Bayley Scales of Infant and Toddler Development [9] | 10 (12%) | [10–19] |
|  | Brunet-Lezine Scale [20] | 3 (4%) | [21–23] |
|  | California Verbal Learning Test [24] | 1 (1%) | [25] |
|  | Contingency Naming Test [26] | 2 (2%) | [27,28] |
|  | Controlled Oral Word Association Test [29] | 1 (1%) | [30] |
|  | Culture Fair Intelligence Test [31] | 3 (4%) | [32–34] |
|  | Denver Developmental Screening Test [35] | 1 (1%) | [36] |
|  | Diagnostic and Statistical Manual of Mental Disorders [37] | 1 (1%) | [38] |
|  | Early Language Milestone Scale [39] | 1 (1%) | [8] |
|  | Faux Pas Test [40] | 2 (2%) | [4,41] |
|  | Go/no-go Task [42] | 2 (2%) | [43,44] |
|  | Kaufman Assessment Battery for Children | 1 (1%) | [45] |
|  | Kaufman Brief Intelligence Test [46] | 3 (4%) | [21,22,47] |
|  | McCarthy Scales of Children's Abilities [48] | 3 (4%) | [13,14,23] |
|  | NEPSY [49] | 1 (1%) | [43] |
|  | Nisonger Child Behavior Rating Form [50] | 1 (1%) | [51] |
|  | Novotni Social Skills Checklist [52] | 1 (1%) | [4] |
|  | Peabody Development Motor Scales [53] | 1 (1%) | [54] |
|  | Perception of Differences Test (FACES) [55] | 1 (1%) | [56] |
|  | Raven Progressive Matrices [57] | 1 (1%) | [58] |
|  | Reading the Mind in the Eyes Test [59] | 2 (2%) | [4,41] |
|  | Rey Auditory Verbal Learning Test [60] | 2 (2%) | [27,28] |
|  | Rey Visual Design Learning Test [61] | 1 (1%) | [28] |
|  | Rey-Osterrieth Complex Figure Test [62] | 3 (4%) | [25,30,56] |
|  | Simple Reaction Time Test [43] | 1 (1%) | [43] |
|  | Social Cognitive Skills Test [63] | 2 (2%) | [4,41] |
|  | Social Skills Rating System [64] | 1 (1%) | [4] |
|  | Stanford-Binet Intelligence Scales [65] | 3 (4%) | [17,66,67] |
|  | Stimulus-Response Compatibility [43] | 1 (1%) | [43] |
|  | Stroop Task [68] | 3 (4%) | [25,33,69] |
|  | Test d-2 [70] | 1 (1%) | [33] |
|  | Test of Language Development [71] | 1 (1%) | [72] |
|  | Test of everyday attention for children [73] | 2 (2%) | [27,28] |
|  | Toulouse Pieron Test [74] | 1 (1%) | [56] |
|  | Vineland Adaptive Behaviour Scales [75] | 1 (1%) | [76] |
|  | Wechsler (age-appropriate version) [77] | 33 (40%) | [2,4,10,12–14,21–23,25,27,28,30,41,43,45,56,72,78–92] |
|  | Unclear | 31 (38%) | [4,41,67,69,85,93–118] |
| **Overall Child Development (n=11)** | Adaptive Behaviour Assessment System [119] | 2 (18%) | [12,117] |
|  | Diagnostic and Statistical Manual of Mental Disorders [37] | 2 (18%) | [38,51] |
|  | Vineland Adaptive Behaviour Scales [75] | 2 (18%) | [19,76] |
|  | Wechsler (age-appropriate version) [77] | 1 (9%) | [51] |
|  | Unclear | 5 (45%) | [112,114,120–122] |
| **Sensorimotor and motor functioning (n=32)** | Ages and Stages Questionnaires [1] | 1 (3%) | [2] |
|  | Amsterdam Neuropsychological Tasks [3] | 3 (9%) | [4,101,123] |
|  | Basic Steps of Development [7] | 1 (3%) | [8] |
|  | Bayley Scales of Infant and Toddler Development [9] | 4 (13%) | [10,16,18,19] |
|  | Brunet-Lezine Scale [20] | 1 (3%) | [23] |
|  | Denver Developmental Screening Test [35] | 1 (3%) | [36] |
|  | Early Language Milestone Scale [39] | 1 (3%) | [8] |
|  | German Number Combination Test [124] | 1 (3%) | [33] |
|  | Landolt C [125] | 1 (3%) | [126] |
|  | McCarthy Scales of Children's Abilities [48] | 1 (3%) | [23] |
|  | Peabody Development Motor Scales [53] | 2 (6%) | [18,54] |
|  | Purdue Pegboard Test [127] | 1 (3%) | [56] |
|  | Rostock-Oseretzky Scale [128] | 2 (6%) | [129,130] |
|  | Trail Making Test [131] | 1 (3%) | [33] |
|  | Vineland Adaptive Behaviour Scales [75] | 1 (3%) | [76] |
|  | Visual Evoked Potentials [132] | 3 (9%) | [16,133,134] |
|  | Wechsler (age-appropriate version) [77] | 2 (6%) | [10,23] |
|  | Unclear | 13 (41%) | [21,67,93,94,111–113,117,126,135–138] |
| **Executive Functioning (n=32)** | 2-Back Task [139] | 1 (3%) | [43] |
|  | Amsterdam Neuropsychological Tasks [3] | 5 (16%) | [4,5,94,123,140] |
|  | Antisaccade Task [141] | 1 (3%) | [142] |
|  | Behavior Rating Inventory of Executive Function [143] | 10 (31%) | [4,12,25,30,94,144–148] |
|  | California Verbal Learning Test [24] | 2 (6%) | [43,69] |
|  | Cambridge Neuropsychological Test Automated Battery [149] | 1 (3%) | [94] |
|  | Children's Memory Scale [150] | 1 (3%) | [43] |
|  | Contingency Naming Test [26] | 3 (9%) | [27,28,30] |
|  | Continuous Performance Test [151] | 1 (3%) | [58] |
|  | Controlled Oral Word Association Test [29] | 1 (3%) | [27] |
|  | Elithorn Test [152] | 2 (6%) | [79,153] |
|  | FAS Test [154] | 1 (3%) | [56] |
|  | Flanker Task [155] | 1 (3%) | [142] |
|  | Go/no-go Task [42] | 3 (9%) | [43,44,142] |
|  | McCarthy Scales of Children's Abilities [48] | 1 (3%) | [69] |
|  | PKU-QoL [156] | 1 (3%) | [157] |
|  | Recognition Span Task [158] | 1 (3%) | [43] |
|  | Rey-Osterrieth Complex Figure Test [62] | 3 (9%) | [27,28,153] |
|  | Stroop Task [68] | 3 (9%) | [25,58,142] |
|  | Tower of London [159] | 3 (9%) | [28,58,153] |
|  | Trail Making Test [131] | 2 (6%) | [33,56] |
|  | Visual Search Test [160] | 1 (3%) | [153] |
|  | Weigl's Sorting Test [161] | 1 (3%) | [153] |
|  | Wechsler (age-appropriate version) [77] | 4 (13%) | [2,27,28,56] |
|  | Wisconsin Card Sorting Test [162] | 4 (13%) | [33,69,79,153] |
|  | Unclear | 9 (28%) | [43,67,114,116–118,135,142,153] |
| **CORE AREA: LIFE IMPACT** | | | |
| **Domain: Child Life Impact** | | | |
| **Child Quality of Life (n=21)** | Child Health Questionnaire [163] | 2 (9%) | [82,164] |
|  | DISABKIDS [165] | 1 (4%) | [166] |
|  | KINDL [167] | 3 (13%) | [168–170] |
|  | PKU-QoL [156] | 1 (4%) | [157] |
|  | PedsQL [171] | 3 (13%) | [164,166,172] |
|  | TNO-AZL [173] | 2 (9%) | [94,174] |
|  | Unclear | 11 (48%) | [4,19,67,93,96,114,116,117,175–177] |
| **Child psychosocial well-being and self-concept (n=15)** | Child Health Questionnaire [156] | 2 (13%) | [82,164] |
|  | DISABKIDS [165] | 1 (7%) | [166] |
|  | Family Systems Illness Model [178] | 1 (7%) | [179] |
|  | KINDL [167] | 3 (20%) | [168–170] |
|  | PKU-QoL [156] | 1 (7%) | [157] |
|  | PedsQL [171] | 2 (13%) | [164,166] |
|  | TNO-AZL [173] | 1 (7%) | [94] |
|  | Unclear | 6 (40%) | [21,67,114,117,118,180] |
| **Child social impact and function (n=12)** | DISABKIDS [165] | 1 (8%) | [166] |
|  | KINDL [167] | 3 (25%) | [168–170] |
|  | Multidimensional Scale for Perceived Social Support [181] | 1 (8%) | [4] |
|  | PKU-QoL [156] | 1 (8%) | [157] |
|  | PedsQL [171] | 2 (17%) | [164,166] |
|  | TNO-AZL [173] | 1 (8%) | [94] |
|  | Test of Anxiety and Depression in Infancy and Adolescence [182] | 1 (8%) | [172] |
|  | Unclear | 3 (25%) | [76,180,183] |
| **Child understanding of and self-efficacy with management of PKU (n=3)** | Unclear | 3 (100%) | [67,183,184] |
| **Domain: Caregiver/family Life impact** | | | |
| **Impact of PKU on caregiver/family quality of life (n=10)** | Child Health Questionnaire [163] | 2 (20%) | [82,164] |
|  | KINDL [167] | 3 (30%) | [168–170] |
|  | PKU-QoL [156] | 1 (10%) | [157] |
|  | PedsQL [171] | 1 (10%) | [164] |
|  | Unclear | 4 (40%) | [176,177,185,186] |
| **Caregiver/family psychosocial well-being (n=9)** | Child Health Questionnaire [163] | 2 (22%) | [82,164] |
|  | Coping Strategies Questionnaire [187] | 1 (11%) | [188] |
|  | PKU-QoL [156] | 1 (11%) | [157] |
|  | Parenting Stress Index [189] | 2 (22%) | [19,76] |
|  | Unclear | 5 (56%) | [76,176,180,188,190] |
| **Domain: Child and Caregiver/family Life Impact** | | | |
| **Perceived control over behaviour and skills (n=2)** | Patient Activation Measure [191] | 1 (50%) | [184] |
|  | Unclear | 1 (50%) | [190] |
| **Domain: Child Behaviour, Mental Health, and Temperament** | | | |
| **Behaviour problems and externalizing mental health or behaviour disorders (n=19)** | Achenbach School-Age Assessment Forms [192] | 5 (26%) | [4,18,174,188,193] |
|  | Behavior Assessment System for Children [194] | 1 (5%) | [12] |
|  | Behavior Rating Inventory of Executive [143] | 5 (26%) | [30,144,145,147,148] |
|  | Beck Anxiety Inventory [195] | 1 (5%) | [12] |
|  | Beck Depression Inventory [196] | 1 (5%) | [12] |
|  | Child Health Questionnaire [163] | 2 (11%) | [82,164] |
|  | Clinical Global Impression [197] | 2 (11%) | [51,144] |
|  | Kiddie Schedule for Affective Disorders and Schizophrenia [198] | 1 (5%) | [51] |
|  | Nisonger Child Behavior Rating Form [50] | 1 (5%) | [51] |
|  | PKU-QoL [156] | 1 (5%) | [157] |
|  | Unclear | 5 (26%) | [21,51,102,117,120] |
| **Attention-deficit hyperactivity disorder (ADHD) or ADHD-like symptoms (n=15)** | ADHD Rating Scale [199] | 5 (33%) | [56,144,145,193,200] |
|  | Achenbach School-Age Assessment Forms [192] | 1 (7%) | [4] |
|  | Behavior Rating Inventory of Executive [143] | 1 (7%) | [56] |
|  | Diagnostic and Statistical Manual of Mental Disorders [37] | 1 (7%) | [66] |
|  | Go/no-go Task [42] | 1 (7%) | [201] |
|  | Kiddie Schedule for Affective Disorders and Schizophrenia [198] | 1 (7%) | [51] |
|  | Nisonger Child Behavior Rating Form [50] | 1 (7%) | [51] |
|  | PKU-QoL [156] | 1 (7%) | [157] |
|  | Unclear | 5 (33%) | [21,67,94,108,117] |
| **Internalizing mental health or mood disorders and associated symptoms (n=26)** | Achenbach School-Age Assessment Forms [192] | 6 (23%) | [4,18,34,174,188,193] |
|  | Adaptive Behaviour Assessment System [119] | 1 (4%) | [202] |
|  | Behavior Rating Inventory of Executive [143] | 2 (8%) | [94,148] |
|  | Beck Depression Inventory [196] | 1 (4%) | [203] |
|  | Diagnostic and Statistical Manual of Mental Disorders [37] | 1 (4%) | [91] |
|  | Hedonic scale [204] | 1 (4%) | [205] |
|  | Nisonger Child Behavior Rating Form [50] | 1 (4%) | [51] |
|  | PKU-QoL [156] | 1 (4%) | [157] |
|  | Pediatric Symptom Checklist [206] | 1 (4%) | [146] |
|  | State-Trait Anxiety Inventory [207] | 1 (4%) | [203] |
|  | Test of Anxiety and Depression in Infancy and Adolescence [182] | 1 (4%) | [172] |
|  | Wechsler (age-appropriate version) [77] | 1 (4%) | [202] |
|  | Unclear | 11 (42%) | [12,21,67,94,96,108,116,117,136,208,209] |
| **Autism spectrum disorder (ASD) or ASD-like symptoms (n=9)** | Autism behaviour checklist [210] | 1 (11%) | [93] |
|  | Bayley [9] | 1 (11%) | [93] |
|  | DISABKIDS [165] | 1 (11%) | [166] |
|  | Diagnostic and Statistical Manual of Mental Disorders [37] | 2 (22%) | [38,66] |
|  | Kiddie Schedule for Affective Disorders and Schizophrenia [198] | 1 (11%) | [51] |
|  | Nisonger Child Behavior Rating Form [50] | 1 (11%) | [51] |
|  | Stanford-Binet Intelligence Scales [65] | 1 (11%) | [93] |
|  | Wechsler (age-appropriate version) [77] | 1 (11%) | [93] |
|  | Unclear | 4 (44%) | [21,116,117,136] |
| **Atypical behavioural and mental symptoms other than those specified (n=1)** | Unclear | 1 (100%) | [117] |
| **Temperament/personality (n=4)** | BIS/BAS Scales [211] | 1 (25%) | [4] |
|  | Groningen Behaviour Checklist School [212] | 1 (25%) | [213] |
|  | Sensitivity to Punishment and Sensitivity to Reward Questionnaire [214] | 1 (25%) | [4] |
|  | TNO-AZL [173] | 1 (25%) | [94] |
|  | Toddler Temperament Questionnaire [215] | 1 (25%) | [18] |

**References**

1. Squires J, Bricker DD. Ages and Stages Questionnaires: A parent-completed child monitoring system. 3rd ed. Baltimore, MD: Brookes; 2009.

2. Aghasi P, Setoodeh A, Sayarifard A, Rashidiyan M, Sayarifard F, Rabbani A, et al. Intellectual and developmental status in children with Hyperphenylalaninemia and PKU who were screened in a national program. Iran J Pediatr. 2015;25:1–5.

3. de Sonneville LMJ. Amsterdamn Neuropsychological Tasks: A computer-aided assessment program. In: Den Brinker BPL., Beek PJ, Mulder LJ., editors. Cogn Ergon Clin Assess Comput Learn. Lisse, The Netherlands: Swets & Zeitlinger; 1999. p. 187–202.

4. Jahja R, Huijbregts SCJ, de Sonneville LMJ, van der Meere JJ, Bosch AM, Hollak CEM, et al. Mental health and social functioning in early treated Phenylketonuria: The PKU-COBESO study. Mol Genet Metab [Internet]. Elsevier Inc.; 2013;110:S57–61. Available from: http://dx.doi.org/10.1016/j.ymgme.2013.10.011

5. Huijbregts S, de Sonnenville L, Licht R, Sergeant J, van Spronsen F. Inhibition of prepoten responding and attentional flexibility in treated phenylketonuria. Dev Neuropsychol. 2002;22:481–99.

6. Huijbregts SCJ, De Sonneville LMJ, Licht R, Van Spronsen F. J, Verkerk PH, Sergeant JA. Sustained attention and inhibition of cognitive interference in treated phenylketonuria: Associations with concurrent and lifetime phenylalanine concentrations. Neuropsychologia. 2002;40:7–15.

7. Perez-Ramos AM. Passos Básicos do Desenvolvimento Infantil: Projeto Multinacional de Educação. Brasilia: MEC/OEA; 1975.

8. Lamônica DAC, Stump MV, Pedro KP, Rolim-Liporacci MC, Caldeira ACGC, Anastácio-Pessan F da L, et al. Breastfeeding follow-up in the treatment of children with phenylketonuria. J Soc Bras Fonoaudiol [Internet]. 2012;24:3816–9. Available from: http://www.ncbi.nlm.nih.gov/pubmed/23306691

9. Bayley N. Bayley Scales of Infant and Toddler Development. 3rd ed. San Antonio, TX: Harcourt Assessment; 2006.

10. Muntau AC, Burlina A, Eyskens F, Freisinger P, De Laet C, Leuzzi V, et al. Efficacy, safety and population pharmacokinetics of sapropterin in PKU patients <4 years: results from the SPARK open-label, multicentre, randomized phase IIIb trial. Orphanet J Rare Dis. Orphanet Journal of Rare Diseases; 2017;12:1–11.

11. Longo N, Siriwardena K, Feigenbaum A, Dimmock D, Burton BK, Stockler S, et al. Long-term developmental progression in infants and young children taking sapropterin for phenylketonuria: a two-year analysis of safety and efficacy. Genet Med [Internet]. 2015;17:365–73. Available from: http://www.nature.com/doifinder/10.1038/gim.2014.109

12. Vockley J, Andersson HC, Antshel KM, Braverman NE, Burton BK, Frazier DM, et al. Phenylalanine hydroxylase deficiency: Diagnosis and management guideline. Genet Med. 2014;16:188–200.

13. Giovanni M, Riva E, Fiori L, Paci S, Verduci E, Agostoni C. Treating phenylketonuria: a single centre experience. J Int Med Res. 2007;35:742–52.

14. Anastasoaie V, Kurzius L, Forbes P, Waisbren S. Stability of blood phenylalanine levels and IQ in children with phenylketonuria. Mol Genet Metab. 2008;95:17–20.

15. Agostoni C, Harvie A, McCulloch DL, Demellweek C, Cockburn F, Giovanni M, et al. A randomized trial of long-chain polyunsaturated fatty acid supplementation in infants with phenylketonuria. Dev Med Child Neurol [Internet]. 2006;48:207–12. Available from: http://ovidsp.ovid.com/ovidweb.cgi?T=JS&PAGE=reference&D=emed7&NEWS=N&AN=2006456892

16. Agostoni C, Verduci E, Massetto N, Radaelli G, Riva E, Giovannini M. Plasma long-chain polyunsaturated fatty acids and neurodevelopment through the first 12 months of life in phenylketonuria. Dev Med Child Neurol. 2003;45:257–61.

17. Trefz FK, Cipcic-Schmidt S, Koch R. Final intelligence in late treated patients with phenylketonuria. Eur J Pediatr [Internet]. 2000;159:S145–8. Available from: http://link.springer.com/10.1007/PL00014380

18. Wu W, Sheng D, Shao J, Zhao Z. Mental and motor development and psychosocial adjustment of Chinese children with phenylketonuria. J Paediatr Child Health. 2011;47:441–7.

19. Waisbren SE, Albers S, Amato S, Ampola M, Brewster TG, Demmer L, et al. Effect of expanded newborn screening for biochemical genetic disorders on child outcomes and parental stress. J Am Med Assoc [Internet]. 2003;290:2564–72. Available from: http://ovidsp.ovid.com/ovidweb.cgi?T=JS&PAGE=reference&D=emed8&NEWS=N&AN=37443286

20. Brunet O, Lezine I. Desenvolvimento psicológico da primeira infância. Porto Alegre: Artes Médicas; 1981.

21. Gonzalez MJ, Gutierrez AP, Gassio R, Fuste ME, Vilaseca MA, Campistol J. Neurological complications and behavioral problems in patients with phenylketonuria in a Follow-up Unit. Mol Genet Metab [Internet]. Elsevier Inc.; 2011;104:S73–9. Available from: http://dx.doi.org/10.1016/j.ymgme.2011.07.015

22. Vilaseca MA, Lambruschini N, Gómez-López L, Gutiérrez A, Fusté E, Gassió R, et al. Quality of dietary control in phenylketonuric patients and its relationship with general intelligence. Nutr Hosp. 2010;25:60–6.

23. Couce ML, Castineiras DE, Bóveda MD, Baña A, Cocho JA, Iglesias AJ, et al. Evaluation and long-term follow-up of infants with inborn errors of metabolism identified in an expanded screening programme. Mol Genet Metab [Internet]. Elsevier Inc.; 2011;104:470–5. Available from: http://dx.doi.org/10.1016/j.ymgme.2011.09.021

24. Delis DC, Kramer JH, Kaplan E, Ober BA. California Verbal Learning Test. 3rd ed. Bloomington, MN: Pearson; 2017.

25. Antshel KM, Waisbren SE. Timing is everything: Executive functions in children exposed to elevated levels of phenylalanine. Neuropsychology. 2003;17:458–68.

26. Anderson VA, Anderson P, Northam E, Taylor HG. Standardisation of the contingency naming test (CNT) for school-aged children: A measure of reactive flexibility. Clin Neuropsychol Assess. 2000;20:385–406.

27. Anderson PJ, Wood SJ, Francis DE, Coleman L, Anderson V, Boneh A. Are neuropsychological impairments in children with early-treated phenylketonuria (PKU) related to white matter abnormalities or elevated phenylalanine levels? Dev Neuropsychol. 2007;32:645–68.

28. Anderson PJ, Wood SJ, Francis DE, Coleman L, Warwick L, Casanelia S, et al. Neuropsychological functioning in children with early-treated phenylketonuria: Impact of white matter abnormalities. Dev Med Child Neurol. 2004;46:230–8.

29. Benton A, Hamsher K, Rey G, Sivan A. Multilingual Aphasia Examination. 3rd ed. Iowa City, IA: AJA Associates; 1994.

30. Anderson VA, Anderson P, Northam E, Jacobs R, Mikiewicz O. Relationships between cognitive and behavioral measures of executive function in children with brain disease. Child Neuropsychol (Neuropsychology, Dev Cogn Sect C) [Internet]. 2002;8:231–40. Available from: http://www.tandfonline.com/doi/abs/10.1076/chin.8.4.231.13509

31. Cattell R. Culture Free Intelligence Test. Champaign, IL: Institute of Personality and Ability Testing; 1949.

32. Couce ML, Vitoria I, Aldámiz-Echevarría L, Fernández-Marmiesse A, Roca I, Llarena M, et al. Lipid profile status and other related factors in patients with Hyperphenylalaninaemia. Orphanet J Rare Dis [Internet]. Orphanet Journal of Rare Diseases; 2016;11:1–12. Available from: http://dx.doi.org/10.1186/s13023-016-0508-x

33. Feldmann R, Denecke J, Pietsch M, Grenzebach M, Weglage J. Phenylketonuria: No specific frontal lobe-dependent neuropsychological deficits of early-treated patients in comparison with diabetics. Pediatr Res. 2002;51:761–5.

34. Weglage J, Grenzebach M, Pietsch M, Feldmann R, Linnenbank R, Denecke J, et al. Behavioural and emotional problems in early-treated adolescents with phenylketonuria in comparison with diabetic patients and healthy controls. J Inherit Metab Dis [Internet]. 2000;23:487–96. Available from: http://www.embase.com/search/results?subaction=viewrecord&from=export&id=L30487880%0Ahttp://dx.doi.org/10.1023/A:1005664231017

35. Frankenburg W, Dodds J, Archer P. Denver II Technical Manual. Denver, CO: Denver Developmental Materials; 1990.

36. da Silva GK, Lamônica DAC. Performance of children with phenylketonuria in the Developmental Screening Test - Denver II. Pró-Fono Rev Atualização Científica. 2010;22:345–50.

37. Association AP. Diagnostic and Statistical Manual of Mental Disorders. 5th ed. Philedelphia, PA: American Psychiatric Association Publishing; 2013.

38. Khemir S, Halayem S, Azzouz H, Siala H, Ferchichi M, Guedria A, et al. Autism in Phenylketonuria Patients: From Clinical Presentation to Molecular Defects. J Child Neurol. 2016;31:843–9.

39. Coplan J. Early Language Milestone Scale. 2nd ed. Austin, TX: PRO-ED, Inc.; 1993.

40. Baron-Cohen S, O’Riordan M, Stone V, Jones R, Plaisted K. Recognition of faux pas by normally developing children and children with asperger syndrome or high-functioning autism. J Autism Dev Disord [Internet]. 1999;29:407–18. Available from: http://ovidsp.ovid.com/ovidweb.cgi?T=JS&PAGE=reference&D=emed4&NEWS=N&AN=1999406651

41. Jahja R, van Spronsen FJ, de Sonneville LMJ, van der Meere JJ, Bosch AM, Hollak CEM, et al. Social-cognitive functioning and social skills in patients with early treated phenylketonuria: a PKU-COBESO study. J Inherit Metab Dis [Internet]. Journal of Inherited Metabolic Disease; 2016;39:355–62. Available from: http://dx.doi.org/10.1007/s10545-016-9918-0

42. Gomez P, Ratcliff R, Perea M. A model of the go/no-go task. J Exp Psychol Gen. 2007;136:389–413.

43. Janos AL, Grange DK, Steiner RD, White DA. Processing speed and executive abilities in children with phenylketonuria. Neuropsychology. 2012;26:735–43.

44. Araujo GC, Christ SE, Steiner RD, Grange DK, Nardos B, McKinstry RC, et al. Response monitoring in children with phenylketonuria. Neuropsychology. 2009;23:130–4.

45. Lambruschini N, Pérez-Dueñas B, Vilaseca MA, Mas A, Artuch R, Gassió R, et al. Clinical and nutritional evaluation of phenylketonuric patients on tetrahydrobiopterin monotherapy. Mol Genet Metab. 2005;86:S54–60.

46. Kaufman AS, Kaufman N. Kaufman Brief Intelligence Test [Internet]. 2nd ed. Bloomington, MN: Pearson; 2004. Available from: https://www.pearsonassessments.com/store/usassessments/en/Store/Professional-Assessments/Cognition-%26-Neuro/Non-Verbal-Ability/Kaufman-Brief-Intelligence-Test-%7C-Second-Edition/p/100000390.html

47. Caprile C, Campistol J, Puigcerver L, Gutiérrez-Mata AP, Alonso-Colmenero I, Colomé R, et al. Subtle visuomotor deficits and reduced benefit from practice in early treated phenylketonuria. J Clin Exp Neuropsychol [Internet]. Routledge; 2017;39:931–40. Available from: http://dx.doi.org/10.1080/13803395.2017.1281381

48. McCarthy D. McCarthy Scales of Children’s Abilities. New York, NY: The Psychological Corporation; 1972.

49. Korkman M, Kirk U, Kemp S. NEPSY [Internet]. 2nd ed. Bloomington, MN: Pearson; 2007. Available from: https://www.pearsonassessments.com/store/usassessments/en/Store/Professional-Assessments/Academic-Learning/Brief/NEPSY-%7C-Second-Edition/p/100000584.html

50. Aman MG, Tasse MJ, Rojahn J, Hammer D. The Nisonger CBRF: A child behavior rating form for children with developmental disabilities. Res Dev Disabil. 1996;17:41–57.

51. Fayyazi A, Salari E, Khajeh A, Gajarpour A. A Comparison of Risperidone and Buspirone for Treatment of Behavior Disorders in Children with Phenylketonuria. Iran J Pediatr. 2014;8:33–8.

52. Michele N. Novotni Social Skills Checklist [Internet]. Weston, FL: ADD WareHouse; 2000. Available from: http://addwarehouse.com/shopsite_sc/store/html/novotni-social-skills-checklist-observer-report.html

53. Folio M, Fewell R. Peabody Developmental Motor Scales: Examiner’s manual. 2nd ed. Austin, TX: PRO-ED; 2000.

54. Nazi S, Rohani F, Sajedi F, Biglarian A, Setoodeh A. Motor development skills of 1- to 4-year-old Iranian children with early treated phenylketonuria. JIMD Rep. 2013;August:85–9.

55. Thurstone LL, Yela M. FACES. Perception of Differences Test. 9th ed. Madrid: TEA ediciones; 2001.

56. Gassió R, Vilaseca MA, Lambruschini N, Boix C, Fusté ME, Campistol J. Cognitive functions in patients with phenylketonuria in long-term treatment with tetrahydrobiopterin. Mol Genet Metab [Internet]. Elsevier Inc.; 2010;99:S75–8. Available from: http://dx.doi.org/10.1016/j.ymgme.2009.10.187

57. Raven J. The Raven’s Progressive Matrices: Change and Stability over Culture and Time. Cogn Psychol. 2000;41:1–48.

58. Azadi B, Seddigh A, Tehrani-Doost M, Alaghband-Rad J, Ashrafi MR. Executive dysfunction in treated phenylketonuric patients. Eur Child Adolesc Psychiatry. 2009;18:360–8.

59. Baron-Cohen S, Wheelwright S, Hill J, Raste Y, Plumb I. The “Reading the Mind in the Eyes” Test revised version: A study with normal adults, and adults with Asperger syndrome or high-functioning autism. J Child Psychol Psychiatry Allied Discip. 2001;42:241–51.

60. Schmidt M. Rey Auditory Verbal Learning Test: A Handbook. Los Angeles, CA: Western Psychological Services; 1996.

61. Strauss E, Sherman EMS, Spreen O. A compendium of neuropsychological tests: Administration, norms and commentary. 3rd ed. New York, NY: Oxford University Press; 2006.

62. Shin M-S, Park S-Y, Park S-R, Seol S-H, Kwon JS. Clinical and empirical applications of the Rey-Osterrieth Complex Figure Test. Nat Protoc. 2006;1:892–9.

63. Van Manen TG, Prins PJM, Emmelkamp PMG. Sociaal cognitieve vaardigheden test: Handleiding. Houten: Bohn Stafleu van Loghum; 2007.

64. Gresham F, Elliott S. Social Skills Rating System: Manual. Circle Pines, MN: American Guidance Service; 1990.

65. Roid GH. Stanford-Binet Intelligence Scales. 5th ed. Itasca, IL: Riverside Publishing;

66. Saad K, Elserogy Y, Abdel rahman AA, Al-Atram AA, Mohamad IL, ElMelegy TTH, et al. ADHD, autism and neuroradiological complications among phenylketonuric children in Upper Egypt. Acta Neurol Belg [Internet]. Springer Milan; 2015;115:657–63. Available from: http://dx.doi.org/10.1007/s13760-014-0422-8

67. Demirkol M, Gizewska M, Giovannini M, Walter J. Follow up of phenylketonuria patients. Mol Genet Metab. 2011;104:S31–9.

68. Stroop JR. Studies of interference in serial verbal reactions. J Exp Psychol. 1935;18:622–43.

69. White DA, Nortz MJ, Mandernach T, Huntington K, Steiner RD. Deficits in memory strategy use related to prefrontal dysfunction during early development: Evidence from children with phenylketonuria. Neuropsychology. 2001;15:221–9.

70. Brickencamp R, Zillmer E. The d2 test of attention. Seattle, WA: Hogrefe & Huber Publishers; 1998.

71. Newcomer P, Hammill D. Test of Language Development-Primary. 5th ed. Torrance, CA: Academic Therapy Publications; 2019.

72. Soleymani Z, Keramati N, Rohani F, Jalaei S. Factors influencing verbal intelligence and spoken language in children with phenylketonuria. Indian Pediatr [Internet]. 2015;52:397–401. Available from: http://www.embase.com/search/results?subaction=viewrecord&from=export&id=L604503508%0Ahttp://dx.doi.org/10.1007/s13312-015-0644-8%0Ahttps://phbibliotek.hosted.exlibrisgroup.com/openurl/45METRO/45METRO_ser vices_page?sid=EMBASE&sid=EMBASE&issn=09747559&id=

73. Manly T, Anderson V, Crawford J, George M, Robertson IH. Test of Everyday Attention for Children. 2nd ed. Bloomington, MN: Pearson; 2016.

74. Toulouse E, Pieron H. Toulouse-Pieron Test. Madrid: TEA ediciones; 1998.

75. Sparrow SS, Cicchetti D V, Saulnier CA. Vineland Adaptive Behavior Scales. 3rd ed. Bloomington, MN: Pearson; 2016.

76. Read CY. The demands of biochemical genetic disorders: A survey of mothers of children with mitochondrial disease or phenylketonuria. J Pediatr Nurs. 2003;18:181–6.

77. Wechsler D. Wechsler Intelligence Scale for Children. 5th ed. Bloomington, MN: Pearson; 2014.

78. García MI, Araya G, Coo S, Waisbren SE, de la Parra A. Treatment adherence during childhood in individuals with phenylketonuria: Early signs of treatment discontinuation. Mol Genet Metab Reports [Internet]. Elsevier; 2017;11:54–8. Available from: http://dx.doi.org/10.1016/j.ymgmr.2017.04.006

79. Nardecchia F, Manti F, Chiarotti F, Carducci C, Carducci C, Leuzzi V. Neurocognitive and neuroimaging outcome of early treated young adult PKU patients: A longitudinal study. Mol Genet Metab [Internet]. Elsevier Inc.; 2015;115:84–90. Available from: http://dx.doi.org/10.1016/j.ymgme.2015.04.003

80. Öztürk Y, Erdur B, Arslansoyu S, Soylar R. Helicobacter pylori infection among children with phenylketonuria. Jpn J Infect Dis. 2013;66:433–5.

81. Viau KS, Wengreen HJ, Ernst SL, Cantor NL, Furtado L V., Longo N. Correlation of age-specific phenylalanine levels with intellectual outcome in patients with phenylketonuria. J Inherit Metab Dis. 2011;34:963–71.

82. Cotugno G, Nicolò R, Cappelletti S, Goffredo BM, Vici CD, Di Ciommo V. Adherence to diet and quality of life in patients with phenylketonuria. Acta Paediatr. 2011;100:1144–9.

83. Pérez-Dueñas B, Pujol J, Soriano-Mas C, Ortiz H, Artuch R, Vilaseca MA, et al. Global and regional volume changes in the brains of patients with phenylketonuria. Neurology. 2006;66:1074–8.

84. Chien YH, Chiang S-C, Huang A, Lin J-M, Chiu Y-N, Chou S-P, et al. Phenylalanine hydroxylase deficiency intelligence of patients after early dietary treatment. Acta Paediatr Taiwanica. 2004;45:320–3.

85. Zhou Z, Yu W, Fukuyama Y, Zheng CN, Wang Z. Clinical analysis of West syndrome associated with phenylketonuria. Brain Dev. 2001;23:552–7.

86. Chang PN, Gray RM, O’Brien LL. Patterns of academic achievement among patients treated early with phenylketonuria. Eur J Pediatr [Internet]. 2000;159:S96–9. Available from: http://www.ncbi.nlm.nih.gov/entrez/query.fcgi?cmd=Retrieve&db=PubMed&dopt=Citation&list_uids=11043153

87. Leuzzi V, Bianchi MC, Tosetti M, Carducci C, Antonozzi I. Clinical significance of brain phenylalanine concentration assessed by in vivo proton magnetic resonance spectroscopy inphenylketonuria. J Inherit Metab Dis. 2000;23:563–70.

88. Greeves LG, Patterson CC, Carson DJ, Thom R, Wolfenden MC, Zschocke J, et al. Effect of genotype on changes in intelligence quotient after dietary relaxation in phenylketonuria and hyperphenylalaninaemia. Arch Dis Child. 2000;82:216–21.

89. Griffiths P V, Demellweek C, Fay N, Robinson PH, Davidson DC. Wechsler subscale IQ and subtest profile in early treated phenylketonuria. Arch Dis Child. 2000;82:209–15.

90. Henderson RM, McCulloch DL, Herbert AM, Robinson PH, Taylor MJ. Visual event-related potentials in children with phenylketonuria. Acta Paediatr [Internet]. 2000;89:52–7. Available from: http://doi.wiley.com/10.1111/j.1651-2227.2000.tb01187.x

91. Manti F, Nardecchia F, Chiarotti F, Carducci C, Carducci C, Leuzzi V. Psychiatric disorders in adolescent and young adult patients with phenylketonuria. Mol Genet Metab [Internet]. Elsevier Inc.; 2016;117:12–8. Available from: http://dx.doi.org/10.1016/j.ymgme.2015.11.006

92. Lundstedt G, Johansson A, Melin L, Alm J. Adjustment and intelligence among children with phenylketonuria in Sweden. Acta Paediatr. 2001;90:1147–52.

93. Yalaz K, Vanli L, Yilmaz E, Tokatli A, Anlar B. Phenylketonuria in pediatric neurology practice: A series of 146 cases. J Child Neurol. 2006;21:987–90.

94. Van Spronsen FJ, Huijbregts SCJ, Bosch AM, Leuzzi V. Cognitive, neurophysiological, neurological and psychosocial outcomes in early-treated PKU-patients: A start toward standardized outcome measurement across development. Mol Genet Metab [Internet]. Elsevier Inc.; 2011;104:S45–51. Available from: http://dx.doi.org/10.1016/j.ymgme.2011.09.036

95. Burton BK, Nowacka M, Hennermann JB, Lipson M, Grange DK, Chakrapani A, et al. Safety of extended treatment with sapropterin dihydrochloride in patients with phenylketonuria: Results of a phase 3b study. Mol Genet Metab [Internet]. Elsevier Inc.; 2011;103:315–22. Available from: http://dx.doi.org/10.1016/j.ymgme.2011.03.020

96. Sharman R, Sullivan KA, Young RM, McGill JJ. Tyrosine monitoring in children with early and continuously treated phenylketonuria: Results of an international practice survey. J Inherit Metab Dis. 2010;33:S417–20.

97. Ipsiroglu OS, Herle M, Spoula E, Möslinger D, Wimmer B, Burgard P, et al. Transcultural pediatrics: Compliance and outcome of PKU patients from families with an immigration background. Wien Klin Wochenschr. 2005;117:541–7.

98. Pérez-Dueñas B, Valls-Solé J, Fernández-Alvarez E, Conill J, Vilaseca MA, Artuch R, et al. Characterization of tremor in phenylketonuric patients. J Neurol. 2005;252:1328–34.

99. Camfield CS, Joseph M, Hurley T, Campbell K, Sanderson S, Camfield PR. Optimal management of phenylketonuria: A centralized expert team is more successful than a decentralized model of care. J Pediatr. 2004;145:53–7.

100. Aoki K. Long term follow-up of patients with inborn errors of metabolism detected by the newborn screening program in Japan. Southeast Asian J Trop Med Public Health. 2003;34 Suppl 3:19–23.

101. Huijbregts SCJ, Van Spronsen FJ, Licht R, De Sonneville LMJ, Berends IE, Verkerk PH, et al. Motor function under lower and higher controlled processing demands in early and continuously treated phenylketonuria. Neuropsychology. 2003;17:369–79.

102. National Institutes of Health Consensus Development Panel. National Institutes of Health Consensus Development Conference statement: Phenylketonuria: Screening and management, October 16-18, 2000. Pediatrics [Internet]. 2001;108:972–82. Available from: http://www.ncbi.nlm.nih.gov/pubmed/11581453

103. Leuzzi V, Seri S, Cerquiglini A, Carducci C, Carducci C, Antonozzi I. Derangement of the dopaminergic system in phenylketonuria: Study of the event-related potential (P300). J Inherit Metab Dis. 2000;23:317–20.

104. Maitusong R, Japaer R, Zheng-yan Z, Ru-lai Y, Xiao-lei H, Hua-qing M. Newborn screening in Zhejiang, China. Chin Med J (Engl). 2012;125:702–4.

105. Yang IL, Mao HQ, Zhang WF, Zhao ZY, Yang RL, Zhou XL, et al. Pitfalls in the management of phenylketonuria in China. Hong Kong J Paediatr. 2012;17:143–7.

106. MacLeod EL, Ney DM. Nutritional management of phenylketonuria. Ann Nestle. 2010;68:58–69.

107. Hinton CF, Homer CJ, Thompson AA, Williams A, Hassell KL, Feuchtbaum L, et al. A framework for assessing outcomes from newborn screening: on the road to measuring its promise. Mol Genet Metab. 2016;118:221–9.

108. Wright EL, Van Hove JLK, Thomas J. Mountain States Genetics Regional Collaborative Center’s metabolic newborn screening long-term follow-up study: A collaborative multi-site approach to newborn screening outcomes research. Genet Med. 2010;12:S228–41.

109. BioMarin Pharmaceutical. A phase 3b open-label study to evaluate the effect of Kuvan® on neurocognitive function, maintenance of blood phenylalanine concentrations, safety, and population pharmacokinetics in young children with phenylketonuria [Internet]. ClinicalTrials.gov. 2009 [cited 2018 May 4]. Available from: https://clinicaltrials.gov/show/NCT00838435%0A

110. BioMarin Pharmaceutical. A phase 4 open-label, single-cohort study of the long-term neurocognitive outcomes in 4 to 5 year-old children with phenylketonuria treated with sapropterin dihydrochloride (Kuvan®) for 7 years [Internet]. ClinicalTrials.gov. 2013 [cited 2018 May 4]. Available from: https://clinicaltrials.gov/show/NCT01965912%0A

111. Koletzko B. Quantitative requirements of docosahexanoic acid for neural function in children with phenylketonuria [Internet]. ClinicalTrials.gov. 2009 [cited 2018 May 4]. Available from: https://clinicaltrials.gov/show/NCT00909012%0A

112. Feuchtbaum L, Dowray S, Lorey F. The context and approach for the California newborn screening short- and long-term follow-up data system: Preliminary findings. Genet Med. 2010;12:242–50.

113. Lindner M, Gramer G, Haege G, Fang-Hoffmann J, Schwab KO, Tacke U, et al. Efficacy and outcome of expanded newborn screening for metabolic diseases - Report of 10 years from South-West Germany. Orphanet J Rare Dis. 2011;6:1–10.

114. van Spronsen FJ, van Wegberg AM, Ahring K, Bélanger-Quintana A, Blau N, Bosch AM, et al. Key European guidelines for the diagnosis and management of patients with phenylketonuria. Lancet Diabetes Endocrinol. 2017;5:743–56.

115. Djordjevic M, Klaasen K, Sarajlija A, Tosic N, Zukic B, Kecman B, et al. Molecular genetics and genotype-based rstimation of BH4-responsiveness in Serbian PKU patients: Spotlight on phenotype implication of p.L48S. JIMD Rep. 2012;October:49–58.

116. Singh RH, Cunningham AC, Mofidi S, Douglas TD, Frazier DM, Hook DG, et al. Updated, web-based nutrition management guideline for PKU: An evidence and consensus based approach. Mol Genet Metab. 2016;118:72–83.

117. Camp KM, Parisi MA, Acosta PB, Berry GT, Bilder DA, Blau N, et al. Phenylketonuria scientific review conference: State of the science and future research needs. Mol Genet Metab. 2014;112:87–122.

118. Gokmen Ozel H, Lammardo AM, Motzfeldt K, Robert M, Rocha JC, van Rijn M, et al. Use of sapropterin in the management of phenylketonuria: Seven case reports. Mol Genet Metab [Internet]. Elsevier Inc.; 2013;108:109–11. Available from: http://dx.doi.org/10.1016/j.ymgme.2012.11.012

119. Harrison P, Oakland T. Adaptive Behavior Assessment System. 3rd ed. Bloomington, MN: Pearson; 2015.

120. Pangkanon S, Charoensiriwatana W, Janejai N, Boonwanich W, Chaisomchit S. Detection of phenylketonuria by the newborn screening program in Thailand. Southeast Asian Jounral Trop Med Public Heal. 2009;40:525–9.

121. Liebl B, Nennstiel-Ratzel U, Roscher A, von Kries R. Data required for the evaluation of newborn screening programmes. Eur J Pediatr. 2003;162:S57–61.

122. Huang X, Yang L, Tong F, Yang R, Zhao Z. Screening for inborn errors of metabolism in high-risk children: A 3-year pilot study in Zhejiang Province, China. BMC Pediatr [Internet]. BioMed Central Ltd; 2012;12:1–7. Available from: http://www.biomedcentral.com/1471-2431/12/18

123. Jahja R, Huijbregts SCJ, De Sonneville LMJ, Van Der Meere JJ, Van Spronsen FJ. Neurocognitive evidence for revision of treatment targets and guidelines for phenylketonuria. J Pediatr [Internet]. Elsevier Ltd; 2014;164:895–9. Available from: http://dx.doi.org/10.1016/j.jpeds.2013.12.015

124. Oswald WD. Zahlen-Verbindungs-Test (ZVT). 3rd ed. Gottingen: Hogrefe; 2016.

125. Kuo HK, Kuo MT, Tiong IS, Wu PC, Chen YJ, Chen CH. Visual acuity as measured with Landolt C chart and Early Treatment of Diabetic Retinopathy Study (ETDRS) chart. Graefe’s Arch Clin Exp Ophthalmol. 2011;249:601–5.

126. De Sonneville LMJ, Huijbregts SCJ, Licht R, Sergeant JA, Van Spronsen FJ. Pre-attentive processing in children with early and continuously-treated PKU. Effects of concurrent Phe level and lifetime dietary control. J Inherit Metab Dis. 2011;34:953–62.

127. Tiffin J, Asher EJ. The Purdue Pegboard: Norms and studies of reliability and validity. J Appl Psychol. 1948;32:234–47.

128. Kurth E. Motometrische Rostock-Oseretzky-Skala. Berlin: Psychodiagnostic Center, Psychology Section of Humbld University; 1985.

129. Koletzko B, Beblo S, Demmelmair H, Müller-Felber W, Hanebutt FL. Does dietary DHA improve neural function in children? Observations in phenylketonuria. Prostaglandins Leukot Essent Fat Acids. 2009;81:159–64.

130. Beblo S, Reinhardt H, Demmelmair H, Muntau AC, Koletzko B. Effect of fish oil supplementation on fatty acid status, coordination, and fine motor skills in children with phenylketonuria. J Pediatr. 2007;150:479–84.

131. Reitan RM. Validity of the trail making test as an indicator of organic brain damage. Percept Mot Skills. 1958;8:271–6.

132. Odom JV, Bach M, Barber C, Brigell M, Marmor MF, Tormene AP, et al. Visual evoked potentials standard (2004). Doc Ophthalmol. 2004;108:115–23.

133. Agostoni C, Verduci E, Massetto N, Fiori L, Radaelli G, Riva E, et al. Long term effects of long chain polyunsaturated fats in hyperphenylalaninemic children. Arch Dis Child [Internet]. 2003;88:582–3. Available from: http://ovidsp.ovid.com/ovidweb.cgi?T=JS&PAGE=reference&D=emed6&NEWS=N&AN=2003262218

134. Beblo S, Reinhardt H, Muntau AC, Mueller-Felber W, Roscher AA, Koletzko B. Fish oil supplementation improves visual evoked potentials in children with phenylketonuria. Neurology. 2001;57:1488–91.

135. White DA, Nortz MJ, Mandernach T, Huntington K, Steiner RD. Age-related working memory impairments in children with prefrontal dysfunction associated with phenylketonuria. J Int Neuropsychol Soc. 2002;8:1–11.

136. Vela-Amieva M, Ibarra-González I, Fernández-Lainez C, Monroy-Santoyo S, Guillén-López S, Belmont-Martínez L, et al. Causes of delay in referral of patients with phenylketonuria to a specialized reference centre in Mexico. J Med Screen. 2011;18:115–20.

137. Brandalize S do RC, Czeresnia D. Evaluation of the program for prevention and health promotion in phenylketonuria patients in Brazil. Rev Saude Publica [Internet]. 2004;38:1–6. Available from: http://ovidsp.ovid.com/ovidweb.cgi?T=JS&PAGE=reference&D=med5&NEWS=N&AN=15122388

138. Mancini PC, Durrant JD, Starling ALP, Iório MCM. Children with phenylketonuria treated early: Basic audiological and electriphysiological evaluation. Ear Hear [Internet]. 2013;34:236–44. Available from: http://content.wkhealth.com/linkback/openurl?sid=WKPTLP:landingpage&an=00003446-201303000-00012

139. Kirchner W. Age differences in short-term retention of rapidly changing information. J Exp Psychol. 1958;55:352–8.

140. Huijbregts SCJ, De Sonneville LMJ, Van Spronsen FJ, Licht R, Sergeant JA. The neuropsychological profile of early and continuously treated phenylketonuria: Orienting, vigilance, and maintenance versus manipulation-functions of working memory. Neurosci Biobehav Rev. 2002;26:697–712.

141. Hallett PE. Primary and secondary saccades to goals defined by instructions. Vision Res. 1978;18:1279–96.

142. Christ SE, Steiner RD, Grange DK, Abrams RA, White DA. Inhibitory control in children With phenylketonuria. Dev Neuropsychol. 2006;30:845–64.

143. Gioia GA, Isquith PK, Guy SC, Kenworthy L. Behavior Rating Inventory of Executive Function. 2nd ed. Lutz, FL: PAR Inc.; 2015.

144. Wyrwich KW, Auguste P, Yu R, Zhang C, Dewees B, Winslow B, et al. Evaluation of neuropsychiatric function in phenylketonuria: Psychometric properties of the ADHD Rating Scale-IV and Adult ADHD Self-Report Scale Inattention Subscale in phenylketonuria. Value Heal [Internet]. Elsevier; 2015;18:404–12. Available from: http://dx.doi.org/10.1016/j.jval.2015.01.008

145. Burton B, Grant M, Feigenbaum A, Singh R, Hendren R, Siriwardena K, et al. A randomized, placebo-controlled, double-blind study of sapropterin to treat ADHD symptoms and executive function impairment in children and adults with sapropterin-responsive phenylketonuria. Mol Genet Metab. 2015;114:415–24.

146. Burton BK, Leviton L, Vespa H, Coon H, Longo N, Lundy BD, et al. A diversified approach for PKU treatment: Routine screening yields high incidence of psychiatric distress in phenylketonuria clinics. Mol Genet Metab [Internet]. Elsevier B.V.; 2013;108:8–12. Available from: http://dx.doi.org/10.1016/j.ymgme.2012.11.003

147. Sharman R, Sullivan K, Young R, Mcgill J. A preliminary investigation of the role of the Phenylalynine:Tyrosine ratio in children with early and continuously treated Phenylketonuria: Toward identification of “Safe” levels. Dev Neuropsychol. 2010;35:57–65.

148. Sharman R, Sullivan K, Young R, Mcgill J. Biochemical markers associated with executive function in adolescents with early and continuously treated phenylketonuria. Clin Genet. 2009;75:169–74.

149. Robbins T, James M, Owen A, Sahakian B, McInnes L, Rabbitt P. Cambridge Neuropsychological Test Automated Battery (CANTAB): A factor analytic study of a large sample of normal elderly volunteers. Dementia. 1994;5:266–81.

150. Cohen M. Children’s Memory Scale. Bloomington, MN: Pearson; 1997.

151. Rosvold HE, Mirsky AF, Sarason I, Bransome Jr. ED, Beck LH. A continuous performance test of brain damage. J Consult Psychol. 1956;20:343–50.

152. Loe BS, Rust J. The Perceptual Maze Test revisited: Evaluating the difficulty of automatically generated mazes. Assessment. 2017;00:1–16.

153. Leuzzi V, Pansini M, Sechi E, Chiarotti F, Carducci C, Levi G, et al. Executive function impairment in early-treated PKU subjects with normal mental development. J Inherit Metab Dis. 2004;27:115–25.

154. Spreen O, Benton AL. Neurosensory center comprehensive examination for aphasia: Manual of directions. Victoria, BC, Canada: Neuropsychology Laboratory, University of Victoria.; 1977.

155. Eriksen BA, Eriksen CE. Effects of noise letters upong the identification of a target letter in a nonsearch task. Percept Psychophys. 1974;16.

156. Regnault A, Burlina A, Cunningham A, Bettiol E, Moreau-Stucker F, Benmedjahed K, et al. Development and psychometric validation of measures to assess the impact of phenylketonuria and its dietary treatment on patients’ and parents’ quality of life: The phenylketonuria - Quality of life (PKU-QOL) questionnaires. Orphanet J Rare Dis [Internet]. ???; 2015;10. Available from: ???

157. Regnault A, Burlina A, Cunningham A, Bettiol E, Moreau-Stucker F, Benmedjahed K, et al. Development and psychometric validation of measures to assess the impact of phenylketonuria and its dietary treatment on patients’ and parents’ quality of life: The phenylketonuria - Quality of life (PKU-QOL) questionnaires. Orphanet J Rare Dis [Internet]. ???; 2015;10:1–18. Available from: ???

158. Satler C, Belham FS, Garcia A, Tomaz C, Tavares MCH. Computerized spatial delayed recognition span task: A specific tool to assess visuospatial working memory. Front Aging Neurosci. 2015;7:1–9.

159. Krikorian R, Bartok J, Gay N. Tower of London Procedure: A standard method and developmental data. J Clin Exp Neuropsychol. 1994;16:840–50.

160. Wolfe JM. Guided Search 2.0 A revised model of visual search. 1994;1:202–38.

161. Hobson P, Meara J, Taylor C. The Weigl Colour-Form Sorting Test: A quick and easily administered bedside screen for dementia and executive dysfunction. Int J Geriatr Psychiatry. 2007;22:909–15.

162. Heaton RK, Chelune GJ, Talley JL, Kay GG, Curtiss G. Wisconsin card sorting test manual: Revised and expanded. Lutz, FL: Psychological Assessment Resources; 1993.

163. HealthActCHQ. CHQ: Child Health Questionnaire. Boston, MA; 2018.

164. Bosch AM, Burlina A, Cunningham A, Bettiol E, Moreau-Stucker F, Koledova E, et al. Assessment of the impact of phenylketonuria and its treatment on quality of life of patients and parents from seven European countries. Orphanet J Rare Dis [Internet]. Orphanet Journal of Rare Diseases; 2015;10:1–14. Available from: http://dx.doi.org/10.1186/s13023-015-0294-x

165. Schmidt S, Debensason D, Muhlan H, Petersen C, Power M, Simeoni MC, et al. The DISABKIDS generic quality of life instrument showed cross-cultural validity. J Clin Epidemiol. 2006;59:587–98.

166. Demirdas S, Maurice-Stam H, Boelen CCA, Hofstede FC, Janssen MCH, Langendonk JG, et al. Evaluation of quality of life in PKU before and after introducing tetrahydrobiopterin (BH4); a prospective multi-center cohort study. Mol Genet Metab [Internet]. Elsevier Inc.; 2013;110:S49–56. Available from: http://dx.doi.org/10.1016/j.ymgme.2013.09.015

167. Ravens-Sieberer U, Bullinger M. KINDLR questionnaire for measuring health-related quality of life in children and adolescents (manual). 2000.

168. Feldmann R, Wolfgart E, Weglage J, Rutsch F. Sapropterin treatment does not enhance the health-related quality of life of patients with phenylketonuria and their parents. Acta Paediatr Int J Paediatr. 2017;106:953–9.

169. Thimm E, Schmidt LE, Heldt K, Spiekerkoetter U. Health-related quality of life in children and adolescents with phenylketonuria: Unimpaired HRQoL in patients but feared school failure in parents. J Inherit Metab Dis. 2013;36:767–72.

170. Ziesch B, Weigel J, Thiele A, Mütze U, Rohde C, Ceglarek U, et al. Tetrahydrobiopterin (BH4) in PKU: Effect on dietary treatment, metabolic control, and quality of life. J Inherit Metab Dis. 2012;35:983–92.

171. Varni JW. The PedsQL Measurement Model for the Pediatric Quality of Life Inventory [Internet]. Web page. [cited 2019 Jul 4]. Available from: https://www.pedsql.org/

172. Cazzorla C, Cegolon L, Burlina AP, Celato A, Massa P, Giordano L, et al. Quality of Life (QoL) assessment in a cohort of patients with phenylketonuria. BMC Public Health [Internet]. 2014;14:1–9. Available from: http://ovidsp.ovid.com/ovidweb.cgi?T=JS&PAGE=reference&D=emed18a&NEWS=N&AN=609246287

173. Fekkes M, Theunissen NCM, Brugman E, Veen S, Verrips EGH, Koopman HM, et al. Development and psychometric evaluation of the TAPQOL: A health-related quality of life instrument for 1-5-year-old children. Qual Life Res. 2000;9:961–72.

174. Landolt MA, Nuoffer JM, Steinmann B, Superti-Furga A. Quality of life and psychologic adjustment in children and adolescents with early treated phenylketonuria can be normal. J Pediatr. 2002;140:516–21.

175. Hatam N, Shirvani S, Javanbakht M, Askarian M, Rastegar M. Cost-utility analysis of neonatal screening program, Shiraz University of Medical Sciences, Shiraz, Iran, 2010. Iran J Pediatr. 2013;23:493–500.

176. Gokmen-Ozel H, Ferguson C, Evans S, Daly A, MacDonald A. Does a lower carbohydrate protein substitute impact on blood phenylalanine control, growth and appetite in children with PKU? Mol Genet Metab [Internet]. Elsevier Inc.; 2011;104:S64–7. Available from: http://dx.doi.org/10.1016/j.ymgme.2011.09.014

177. Rohde C, Thiele AG, Och U, Schönherr K, Meyer U, Rosenbaum-Fabian S, et al. Effect of dietary regime on metabolic control in phenylketonuria: Is exact calculation of phenylalanine intake really necessary? Mol Genet Metab Reports [Internet]. The Authors; 2015;5:36–41. Available from: http://dx.doi.org/10.1016/j.ymgmr.2015.09.006

178. Rolland JS, D M. Anticipatory loss: A family systems developmental framework. Fam Process. 1990;29:229–44.

179. Weber SL, Segal S, Packman W. Inborn errors of metabolism: Psychosocial challenges and proposed family systems model of intervention. Mol Genet Metab [Internet]. Elsevier B.V.; 2012;105:537–41. Available from: http://dx.doi.org/10.1016/j.ymgme.2012.01.014

180. Zwiesele S, Bannick A, Trepanier A. Parental strategies to help children with phenylketonuria (PKU) cope with feeling different. Am J Med Genet Part A. 2015;167A:1787–95.

181. Zimet GD, Dahlem NW, Zimet SG, Farley GK. The Multidimensional Scale of Perceived Social Support. J Pers Assess. 2010;52:30–41.

182. Newcomer P, Barenbaum E, Bryant B. TAD: Test for Anxiety and Depression. Trento: Erickson Ed; 2003.

183. Singh RH, Kable JA, Guerrero N V., Sullivan KM, Elsas II LJ. Impact of a camp experience on phenylalanine levels, knowledge, attitudes, and health beliefs relevant to nutrition management of phenylketonuria in adolescent girls. J. Am. Diet. Assoc. 2000. p. 797–803.

184. Viau KS, Jones JL, Murtaugh MA, Gren LH, Stanford JB, Bilder DA. Phone-based motivational interviewing to increase self-efficacy in individuals with phenylketonuria. Mol Genet Metab Reports [Internet]. The Authors; 2016;6:27–33. Available from: http://dx.doi.org/10.1016/j.ymgmr.2016.01.002

185. Eijgelshoven I, Demirdas S, Smith TA, Van Loon JMT, Latour S, Bosch AM. The time consuming nature of phenylketonuria: A cross-sectional study investigating time burden and costs of phenylketonuria in the Netherlands. Mol Genet Metab [Internet]. Elsevier Inc.; 2013;109:237–42. Available from: http://dx.doi.org/10.1016/j.ymgme.2013.05.003

186. Sojoodi T. Effect of maternal empowerment program on burden of care in mothers of children with PKU [Internet]. Int. Clin. Trials Regist. Platf. 2017 [cited 2018 May 4]. Available from: http://apps.who.int/trialsearch/Trial2.aspx?TrialID=IRCT2016071828975N1

187. Elkiit A. Coping styles questionnaire: A contribution to the validation of a scale for measuring coping strategies. Pers Individ Dif. 1996;21:809–12.

188. Jusiene R, Kucinskas V. Psychological adjustment of children with congenital hypothyroidism and phenylketonuria as related to parental psychological adjustment. Med [Internet]. 2004;40:663–70. Available from: http://www.ncbi.nlm.nih.gov/pubmed/15252232

189. Abidin RR. Parenting Stress Index. 4th ed. Lutz, FL: PAR;

190. Antshel KM, Brewster S, Waisbren SE. Child and parent attributions in chronic pediatric conditions: Phenylketonuria (PKU) as an exemplar. J Child Psychol Psychiatry. 2004;45:622–30.

191. Hibbard JH, Mahoney ER, Stockard J, Tusler M. Development and testing of a short form of the patient activation measure. Heal Res Educ Trust. 2005;40:1918–30.

192. Achenbach TM, Rescorla LA. ASEBA school-age forms and profiles [Internet]. Burlington, VT: ASEBA; 2001. Available from: https://store.aseba.org/MANUAL-FOR-THE-ASEBA-SCHOOL-AGE-FORMS-PROFILES/productinfo/505/

193. Smith M Lou, Saltzman J, Klim P, Hanley WB, Feigenbaum A, Clarke JTR. Neuropsychological function in mild hyperphenylalaninemia. Am J Ment Retard. 2000;105:69–80.

194. Reynolds CR, Kamphaus RW. Behaviour Assessment System for Children [Internet]. 3rd ed. Bloomington, MN: Pearson; 2015. Available from: https://www.pearsonassessments.com/store/usassessments/en/Store/Professional-Assessments/Behavior/Comprehensive/Behavior-Assessment-System-for-Children-%7C-Third-Edition-/p/100001402.html

195. Beck AT, Brown G, Epstein N, Steer RA. An inventory for measuring clinical anxiety: Psychometric properties. J Consult Clin Psychol. 1988;56:893–7.

196. Beck AT, Steer RA, Brown GK. Beck Depression Inventory [Internet]. 2nd ed. Bloomington, MN: Pearson; 1996. Available from: https://www.pearsonassessments.com/store/usassessments/en/Store/Professional-Assessments/Personality-%26-Biopsychosocial/Beck-Depression-Inventory-II/p/100000159.html

197. Busner J, Targum SD. The Global Impressions Scale: Applying a research tool in clinical practice. Psychiatry. 2007;4:29–37.

198. Kaufman J, Birmaher B, Brent D, Rao U, Flynn C, Moreci P, et al. Schedule for affective disorders and schizophrenia for school-age children-present and lifetime version (K-SADS-PL): Initial reliability and validity data. J Am Acad Child Adolesc Psychiatry [Internet]. The American Academy of Child and Adolescent Psychiatry; 1997;36:980–8. Available from: http://dx.doi.org/10.1097/00004583-199707000-00021

199. DuPaul GJ, Power TJ, Anastopoulos AD, Reid R. ADHD Rating Scale-5 for Children and Adolescents: Checklists, norms, and clinical interpretation [Internet]. New York, NY: Guilford Press; 2016. Available from: https://psycnet.apa.org/record/2016-17532-000

200. Antshel KM, Waisbren SE. Developmental timing of exposure to elevated levels of phenylalanine is associated with ADHD symptom expression. J Abnorm Child Psychol. 2003;31:565–74.

201. Wiersema JR, van der Meere JJ, Roeyers H. State regulation and response inhibition in children with ADHD and children with early- and continuously treated phenylketonuria: An event-related potential comparison. J Inherit Metab Dis. 2005;28:831–43.

202. Karam PE, Daher RT, Moller LB, Mikati MA. Experience with hyperphenylalaninemia in a developing country: Unusual clinical manifestations and a novel gene mutation. J Child Neurol. 2011;26:142–6.

203. Gunduz M, Arslan N, Unal O, Cakar S, Kuyum P, Bulbul SF. Depression and anxiety among parents of phenylketonuria children. Neurosciences. 2015;20:350–6.

204. Chen AW, Resurreccion AVA, Paguio LP. Age appropriate hedonic scales to measure food preferences of young children. J Sens Stud. 2007;11:2007.

205. Evans S, Daly A, Chahal S, MacDonald J, MacDonald A. Food acceptance and neophobia in children with phenylketonuria: A prospective controlled study. J Hum Nutr Diet. 2016;29:427–33.

206. Jellinek MS, Murphy JM, Little M, Pagano ME, Comer DM, Kelleher KJ. Use of the pediatric symptom checklist to screen for behaviour problems in children: A national feasibility study. Arch Pediatr Adolesc Med. 1999;153:254–60.

207. Spielberger CD. State-Trait Anxiety Inventory. Corsini Encycl Psychol. Hoboken, NY: Wiley; 2010.

208. Cunningham A, Bausell H, Brown M, Chapman M, DeFouw K, Ernst S, et al. Recommendations for the use of sapropterin in phenylketonuria. Mol Genet Metab [Internet]. Elsevier Inc.; 2012;106:269–76. Available from: http://dx.doi.org/10.1016/j.ymgme.2012.04.004

209. Longo N, Arnold GL, Pridjian G, Enns GM, Ficicioglu C, Parker S, et al. Long-term safety and efficacy of sapropterin: The PKUDOS registry experience. Mol Genet Metab [Internet]. Elsevier B.V.; 2015;114:557–63. Available from: http://dx.doi.org/10.1016/j.ymgme.2015.02.003

210. Krug DA, Arick J, Almond P. Behavior checklist for identifying severely handicapped individuals with high levels of autistic behavior. J Child Psychol Psychiatry. 1980;21:221–9.

211. Carver CS, White TL. Behavioral inhibition, behavioral activation, and affective responses to impending reward and punishment: The BIS/BAS Scales. J Pers Soc Psychol. 1994;67:319–33.

212. Kalverboer AF, Visser J. Manual to GBS, Groningen Behaviour Checklist School situation. Amsterdam/Lisse: Swets & Zeitlinger; 2000.

213. Stemerdink BA, Kalverboer AF, Van Der Meere JJ, Van Der Molen MW, Huisman J, De Jong LWA, et al. Behaviour and school achievement in patients with early and continuously treated phenylketonuria. J Inherit Metab Dis. 2000;23:548–62.

214. Torrubia R, Avila C, Molto J, Caseras X. The Sensitivity to Punishment and Sensitivity to Reward Questionnaire (SPSRQ) as a measure of Gray’s anxiety and impulsivity dimensions. Pers Individ Dif. 2001;31:837–62.

215. Fullard W, McDevitt SC, Carey WB. Assessing temperament in one- to three-year-old children. J Pediatr Psychol. 1984;9:205–17.
